# Identifying treatment options for *BRAF*^V600^ wild-type metastatic melanoma: A SU2C/MRA genomics-enabled clinical trial

**DOI:** 10.1371/journal.pone.0248097

**Published:** 2021-04-07

**Authors:** Patricia M. LoRusso, Aleksandar Sekulic, Jeffrey A. Sosman, Winnie S. Liang, John Carpten, David W. Craig, David B. Solit, Alan H. Bryce, Jeffrey A. Kiefer, Jessica Aldrich, Sara Nasser, Rebecca Halperin, Sara A. Byron, Mary Jo Pilat, Scott A. Boerner, Diane Durecki, William P. D. Hendricks, Daniel Enriquez, Tyler Izatt, Jonathan Keats, Christophe Legendre, Svetomir N. Markovic, Amy Weise, Fatima Naveed, Jessica Schmidt, Gargi D. Basu, Shobana Sekar, Jonathan Adkins, Erica Tassone, Karthigayini Sivaprakasam, Victoria Zismann, Valerie S. Calvert, Emanuel F. Petricoin, Leslie Anne Fecher, Christopher Lao, J. Paul Eder, Nicholas J. Vogelzang, Jane Perlmutter, Mark Gorman, Barbara Manica, Lisa Fox, Nicholas Schork, Daniel Zelterman, Michelle DeVeaux, Richard W. Joseph, C. Lance Cowey, Jeffrey M. Trent

**Affiliations:** 1 Yale Cancer Center, Yale University, New Haven, CT, United States of America; 2 Mayo Clinic, Scottsdale, AZ, United States of America; 3 Translational Genomics Research Institute, Phoenix, AZ, United States of America; 4 Robert H. Lurie Comprehensive Cancer Center, Northwestern University, Evanston, IL, United States of America; 5 Keck School of Medicine, University of Southern California, Los Angeles, CA, United States of America; 6 Memorial Sloan Kettering Cancer Center, New York, NY, United States of America; 7 Eugene Applebaum College of Pharmacy and Health Sciences, Wayne State University, Detroit, MI, United States of America; 8 Mayo Clinic, Rochester, MN, United States of America; 9 Barbara Ann Karmanos Cancer Institute, Wayne State University, Detroit, MI, United States of America; 10 Center for Applied Proteomics and Molecular Medicine, George Mason University, Manassas, VA, United States of America; 11 University of Michigan Comprehensive Cancer Center, University of Michigan, Ann Arbor, MI, United States of America; 12 US Oncology, United States of America; 13 Gemini Group, Ann Arbor, MI, United States of America; 14 Silver Spring, MD, United States of America; 15 Regeneron Pharmaceuticals, Tarrytown, NY, United States of America; 16 Mayo Clinic, Jacksonville, FL, United States of America; 17 Charles A. Sammons Cancer Center/Baylor University Medical Center, Dallas, TX, United States of America; Universidade do Minho, PORTUGAL

## Abstract

Although combination BRAF and MEK inhibitors are highly effective for the 40–50% of cutaneous metastatic melanomas harboring *BRAF*^V600^ mutations, targeted agents have been ineffective for *BRAF*^V600^wild-type (wt) metastatic melanomas. The SU2C Genomics-Enabled Medicine for Melanoma Trial utilized a Simon two-stage optimal design to assess whether comprehensive genomic profiling improves selection of molecular-based therapies for *BRAF*^V600^wt metastatic melanoma patients who had progressed on standard-of-care therapy, which may include immunotherapy. Of the response-evaluable patients, binimetinib was selected for 20 patients randomized to the genomics-enabled arm, and nine were treated on the alternate treatment arm. Response rates for 27 patients treated with targeted recommendations included one (4%) partial response, 18 (67%) with stable disease, and eight (30%) with progressive disease. Post-trial genomic and protein pathway activation mapping identified additional drug classes that may be considered for future studies. Our results highlight the complexity and heterogeneity of metastatic melanomas, as well as how the lack of response in this trial may be associated with limitations including monotherapy drug selection and the dearth of available single and combination molecularly-driven therapies to treat *BRAF*^V600^wt metastatic melanomas.

## Introduction

Historically, patients with advanced metastatic melanoma (MM) have had a poor prognosis with a median survival of about six to nine months and a five-year survival of approximately ten percent [1, 2]. Immune checkpoint inhibitors and combination targeted therapy for *BRAF*^V600^ mutant melanoma in large clinical trials have demonstrated a significant therapeutic advance with an increased progression free survival (PFS) to 20–30%, and an overall survival of approximately 50% at five years. This improvement led to the United States Food and Drug Administration (FDA) approval of nivolumab, pembrolizumab, or the combination of ipilimumab/nivolumab, for all MM [3–6].

With respect to molecularly targeted approaches, BRAF (proto-oncogene B-raf) inhibitors, alone or in combination with MEK inhibitors, have demonstrated clinical efficacy in the majority of patients whose tumors harbor oncogenic *BRAF*^V600E/K^ mutations [7–10]. However, little progress has been made in identifying effective therapeutic options for targeted treatment of patients with wild-type *BRAF* (*BRAF*^V600^wt) tumors, which comprise at least 50% of all MMs [11]. In addition to *BRAF*, activating *NRAS* mutations in cutaneous melanomas (CMs) occur at a rate of 15 to 25% [12–15], and ten to 15% of melanomas have alterations leading to loss of function of *NF1*. The remaining five to 15% of melanomas includes a variety of genetic alterations (e.g. cKIT, *BRAF*^non-V600^, H-RAS, K-RAS, MEK, etc), all leading to MAPK pathway activation. In general, melanomas originating from sites with chronically sun-damaged skin that are frequently found in a more elderly population have increased overall mutational load compared to MMs originating from areas of skin that had intermittent sun-damage [12, 16, 17]. This highlights the importance of identifying effective therapeutic approaches to identify molecular targets for this large subset of melanoma patients.

To address the unmet clinical need for novel treatments for *BRAF* ^V600^wt MM, the G.E.M.M. (Genomics-Enabled Medicine for Melanoma) Trial was initially designed to test whether comprehensive molecular interrogation of a patient’s tumor to select therapy improves patient outcome compared to using an alternate available treatment (AAT), which may include physician’s choice, to select therapy for MM. This trial was open to MM patients of any histological subtype, including cutaneous (CM), mucosal (MU), uveal (UM), and acral (AM), as well as patients for whom the melanoma’s primary site was unknown (MUP), as the trial was aimed at testing genomically-selected treatments for *BRAF*^V600^wt disease. We previously performed a pilot feasibility study [18] in five melanoma patients to benchmark all procedures surrounding sample processing, sequencing, data analysis, report generation, and the Molecular Tumor Board (MTB) to select a treatment plan. From this pilot, a Simon two-stage optimal design, sequencing approach, and analytical workflow were implemented within the context of the Phase II, prospective, multi-center, open-label trial described here, for which 37 *BRAF* ^V600^wt MM patients were enrolled (FDA IND#115,393; ClinicalTrials.gov Identifier NCT02094872). Tumor/normal analyses using whole exome and RNA sequencing were used to identify somatic alterations with the intention of formulating a treatment plan. Patients were initially randomized to either AAT or targeted therapy determined based on genomic profiling, with a subsequent protocol amendment removing the AAT arm. Targeted therapy was determined by the MTB after review of the patient’s genomics data and the available targeted therapeutics in the drug pharmacopeia we had secured. Herein, we describe results from our trial, as well as findings from post-trial genomics and protein pathway activation mapping analyses.

## Results

**Fig 1:**
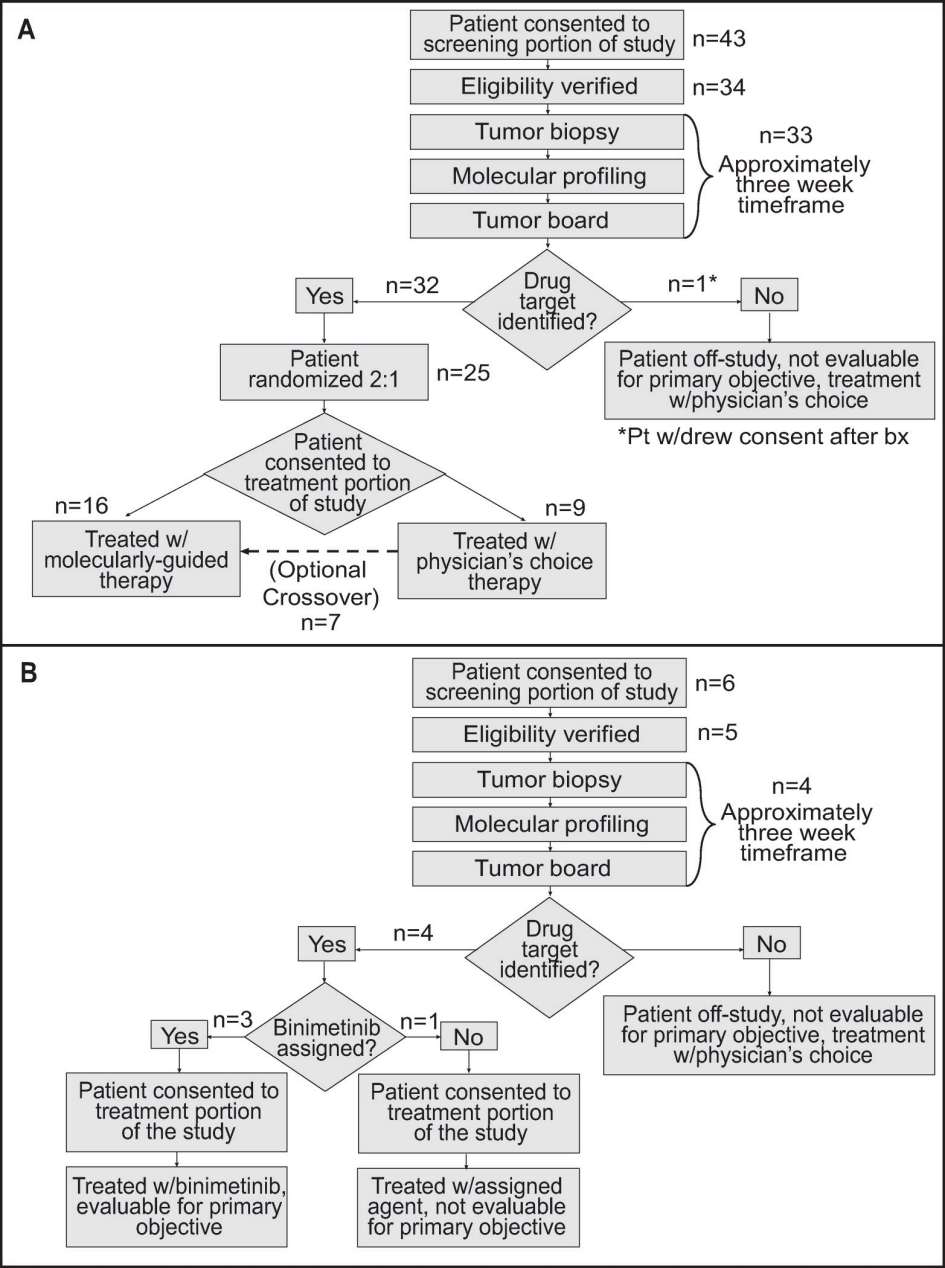
Clinical trial design (CONSORT flow diagrams). A. Original trial design. B. Updated design after the trial was amended midway to allow patients with *NRAS* mutations to be treated with binimetinib.

Forty-nine patients with *BRAF*^V600^wt MM were consented between June 2014 and December 2016. All patients who had received prior treatments experienced disease progression prior to enrolling onto this trial. Of the 49 MM patients, 37 patients (CM, n = 14 [38%]; MU, n = 7 [19%]; UM, n = 9 [24%]; AM, n = 5 [14%]; MUP, n = 2 [5%]) advanced to undergoing biopsies for genomic analysis (Fig 1). Biopsy sampling on two patients resulted in inadequate tissue, while eight patients did not meet eligibility to continue to biopsy. Two patients withdrew consent prior to any procedures being performed. One patient withdrew consent after the tumor biopsy was performed, leaving 36 patients for whom genomically-determined treatment was recommended by the MTB. Core needle or surgical biopsies were collected from each patient and paired tumor/normal whole exome and tumor RNA sequencing were performed. Variant calling and drug rule matching were performed for each patient to generate a personalized molecular report, which was then reviewed by both a molecular and clinical tumor board. These boards identified a monotherapy (no combination regimens were available) strategy based on a pre-defined study pharmacopeia of FDA-approved or investigational drugs [18] (S1 Table). Although drug combination therapy was not allowed due to the absence of safety data of combination therapies at the time of protocol recruitment, the MTB discussed genomic changes that identified multiple drugs and the theoretical utilization of drug combinations for 25 patients. Phenotypic and clinical information for each patient is shown in Table 1 (see S2 Table for additional clinical data). The median time between the performance of tumor biopsy and convening of the MTB, which took place only when the completion of genomic analyses was complete, was 22 days (range 15–30). The median time between performance of biopsy and treatment was 35 days (range 22–72).

**Table 1:** Phenotypic patient data.

Patient	Gender	Age	Race	Site of disease	Breslow Depth (mm)	Clarks Level	Mitoses (mm2)	Stage M	Stage N	Stage T	BRAF Mutation Status	Ulceration	Previous Cancers	Genomics Report Generation Date (months from date of dx)
SM0001	M	44	Caucasian	Cutaneous	1.7	IV	1	M0	N1	T2	N/A	Yes	None	32
SM0002	F	79	Caucasian	Acral	1.7	Unknown	1–5	Unknown	Unknown	T2	Unknown	Yes	None	5
SM0003	F	77	Caucasian	Cutaneous	0.65	III	None	M0	N0	T3	V600E/K/D negative	No	None	35
SM0004	M	62	Caucasian	Uveal	N/A	N/A	None	M1	N1	T1	V600E/K negative	N/A	None	37
SM0005	M	69	Caucasian	Uveal	N/A	N/A	Yes	N/A	N/A	N/A	V600E/K negative	N/A	Early stage prostate cancer (2009)	38
SM0006	M	58	Caucasian	Uveal	Unknown	Unknown	None	M1	Unknown	Unknown	Wild-Type	N/A	Seminoma	15
SM0007	M	70	Caucasian	Cutaneous	12	IV	None	N/A	N/A	N/A	Wild-Type	Yes	None	94
SM0008	F	74	Caucasian	Cutaneous	6.0	Unknown	None	Unknown	Unknown	Unknown	Wild-Type	No	Infiltrating ductal carcinoma (Right breast, 2007)	93
SM0009	F	49	Caucasian	Mucosal	1.1	IV	1–5	Unknown	Unknown	T2	Wild-Type	Yes	None	12
SM0010	F	55	Caucasian	Uveal	Unknown	N/A	None	N/A	N/A	N/A	Wild-Type	N/A	NA	70
SM0011	F	79	Caucasian	Uveal	Unknown	Unknown	None	Unknown	Unknown	Unknown	Wild-Type	N/A	None	N/A
SM0012	F	56	Caucasian	Cutaneous	1.6	IV	None	M0	N1	T2	Wild-Type	No	None	77
SM0013	M	51	Caucasian	Uveal	N/A	N/A	None	N/A	N/A	N/A	Wild-Type	N/A	None	26
SM0014	M	29	Caucasian	Acral	7.0	V	1–5	M0	N3	T4	Wild-Type	No	None	12
SM0015	F	73	Caucasian	Mucosal	1.55	IV	None	M0	N2	T4	Wild-Type	Yes	Uterine cancer (1995), Squamous cell carcinoma (in situ, 2014)	16
SM0016	M	71	Caucasian	Uveal	11.3	N/A	None	Unknown	Unknown	N/A	Wild-Type	N/A	None	32
SM0017	M	62	Caucasian	Uveal	5.80	N/A	None	N/A	N/A	Unknown	Wild-Type	N/A	None	30
SM0018	M	63	Caucasian	Acral	6	V	None	M0	N0	T4	Wild-Type	No	None	44
SM0019	F	68	African American	Mucosal	N/A	N/A	None	M0	N0	T4	Wild-Type	N/A	None	26
SM0020	F	62	Caucasian	Cutaneous	2	II	<1	M1	N0	T1	Wild-Type	No	None	62
SM0021	F	61	Caucasian	Mucosal	3.2	IV	<1	N/A	N/A	N/A	Wild-Type	Yes	N/A	65
SM0022	F	62	Caucasian	Cutaneous	N/A	N/A	None	N/A	N/A	N/A	Wild-Type	N/A	None	5
SM0023	M	48	Caucasian	Cutaneous	N/A	Unknown	None	M1	N3	T3	Wild-Type	N/A	None	3
SM0024	F	72	Caucasian	Acral	N/A	N/A	None	N/A	N/A	N/A	Wild-Type	N/A	None	N/A
SM0025	F	65	Caucasian	Mucosal	Unknown	Unknown	None	M0	Unknown	T3	Wild-Type	No	None	25
SM0026	M	77	Caucasian	Cutaneous	Unknown	Unknown	None	M0	N2	T3	Wild-Type	N/A	None	N/A
SM0027	F	69	Caucasian	Unknown	Unknown	Unknown	None	Unknown	N2	Unknown	Wild-Type	N/A	None	18
SM0028	F	73	Caucasian	Cutaneous	13	N/A	None	M0	N0	T4	Wild-Type	Yes	Breast cancer	16
SM0029	F	74	Caucasian	Cutaneous	6	V	<1	N/A	N0	T4	V600E/K/D negative	No	Basal cell carcinoma (right lateral knee)	23
SM0030	M	64	Caucasian	Uveal	N/A	N/A	1–5	N/A	N/A	N/A	Wild-Type	N/A	None	18
SM0031	M	69	Caucasian	Acral	4	V	1	M0	N0	T4	V600E/K negative	Yes	None	33
SM0032	F	42	Caucasian	Mucosal	N/A	Unknown	None	M1	Unknown	Unknown	Wild-Type	N/A	None	27
SM0033	M	49	Caucasian	Mucosal	N/A	N/A	None	N/A	N/A	N/A	Wild-Type	N/A	None	N/A
SM0034	M	66	Caucasian	Cutaneous	0.75	N/A	None	N/A	N/A	N/A	Wild-Type	N/A	None	66
SM0035	M	70	Caucasian	Cutaneous	4	V	None	M0	N0	T4	Wild-Type	N/A	None	63
SM0036	F	48	Caucasian	Uveal	N/A	N/A	None	M0	N0	T3	Wild-Type	N/A	None	83

### Clinical trial results

**Fig 2:**
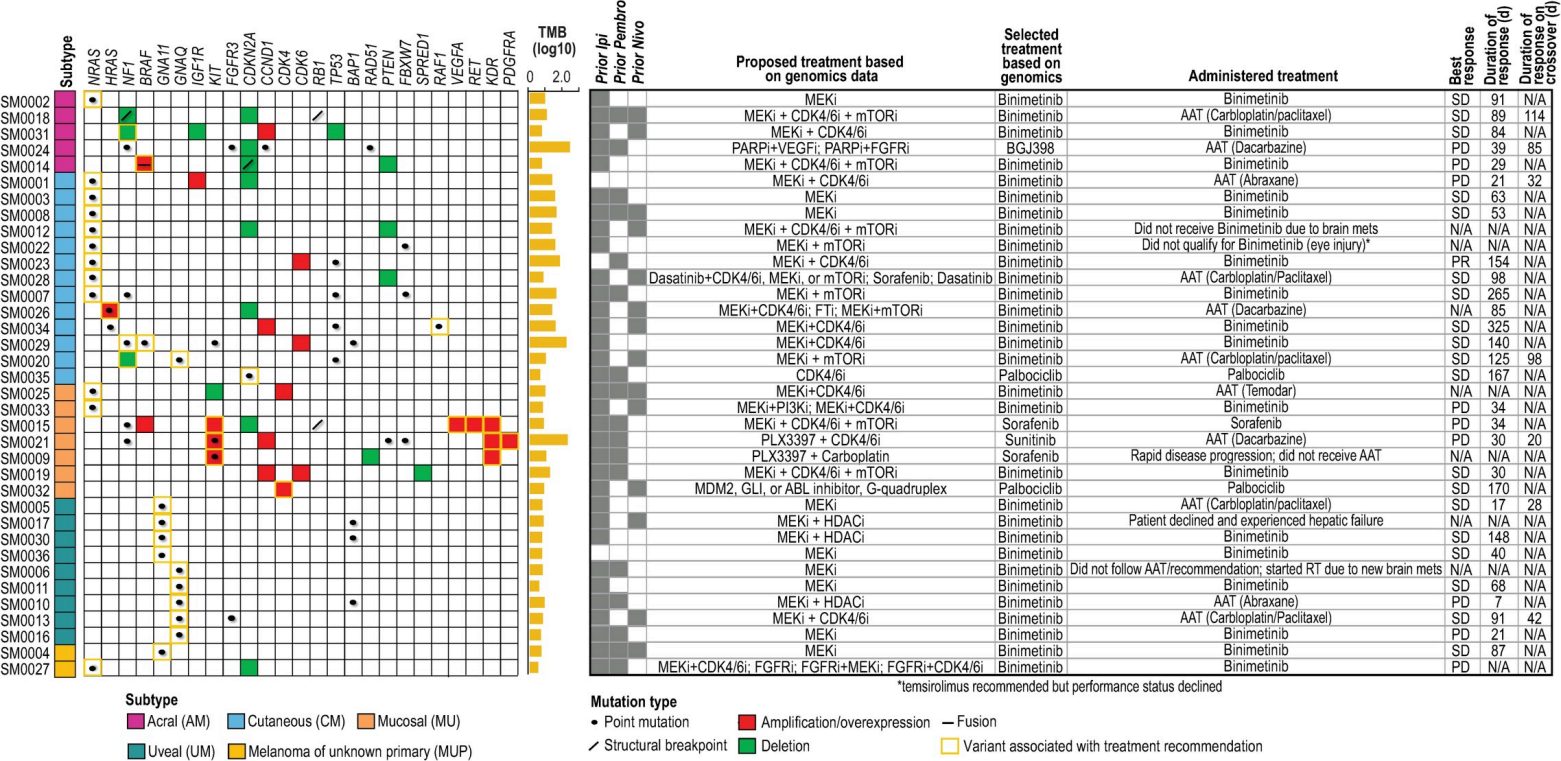
Summary oncoprint and selected treatments. Alterations associated with drug matches are shown along with tumor mutation burden (TMB; number of mutations per Mb, log10), proposed and selected treatments, and responses. Proposed treatments are those that would have been recommended if combinations or if drugs outside the trial’s pharmacopeia were allowed. AAT: alternative available treatment; RT: radiotherapy.

Initially, Oncore Clinical Research software was used to randomize molecular therapy versus AAT (2:1; Fig 1A). However, the trial was amended after consent of patient number 43 to allow all patients with *NRAS* mutations to be treated with binimetinib (MEK162) based on MTB recommendations and preliminary reported data from other clinical trials (Fig 1B) [19–21]. Overall, 69% (25/36) of patients were randomized to targeted therapy as their initial treatment and 31% (11/36) were randomized to AAT. Recommended treatments, which were determined as previously described [18], for each patient are shown in Fig 2. In 83% (30/36) of patients, treatment with the MEK inhibitor, binimetinib, as a single agent was recommended. Recommendations for the remaining 6 patients included palbociclib (2), sorafenib (2), sunitinib (1), and the pan-FGFR kinase inhibitor BGJ398 (infigratinib) (1). Treatment choices for AAT included nab-paclitaxel, carboplatin/paclitaxel, dacarbazine, or temozolomide, since all, except for some of the UM patients, had received prior immune checkpoint therapy. AAT treatment selection for each patient was performed by the patient’s treating physician.

Of the initial 49 patients enrolled, 29 patients completed the biopsy requirement, genomic profiling, and clinical evaluation. Twenty-nine MM patients (CM, n = 10 [34%]; MU, n = 5 [17%]; UM, n = 7 [24%]; AM, n = 5 [17%]; MUP, n = 2 [7%]) were treated on trial. Of the remaining eight patients who consented to study and underwent biopsy but did not receive treatment, two did not have adequate tissue for full analysis, five had intervening disease progression or declining performance status before the recommended treatment could be initiated, and one withdrew consent after the biopsy was performed (genomic analysis was not performed). The median age of those who were treated was 68 years (range 29–79); fourteen (48%) were male, and 15 (52%) were female. Participants identified their race as primarily Caucasian (n = 28, 97%), with one Black participant (MU). Twenty-seven out of 29 patients (93%) had previously been treated with one or more immune checkpoint inhibitors (Fig 2); 83% (24/29) had received ipilimumab, 48% (14/29) pembrolizumab, 31% (9/29) nivolumab, and 7% (2/29) had received combination ipilimumab and nivolumab. Two patients had not received prior immunotherapy due to clinical contra-indication or patient preference. In addition to the above-described immunotherapy treatment for melanoma in the metastatic setting, 48% (14/29) of patients had received additional treatments (including previous investigational agents on clinical trials; Fig 2); 7% (2/29) had not received any prior systemic therapy.

Of the 29 Response Evaluation Criteria In Solid Tumors (RECIST) evaluable patients, 20 patients were initially treated with targeted therapy and nine with AAT. Seven of the nine patients treated with AAT crossed over to the MTB-recommended targeted therapy upon progression. One CM patient (SM0023) out of 20 patients treated with binimetinib demonstrated a partial response (PR; 0.001, 0.25 exact 95% confidence interval) and was taken off study due to a new lesion after 154 days on treatment, for which the dose was reduced due to grade three hypertension. Overall, 19/29 patients (66%; 0.457, 0.821 exact 95% confidence interval) demonstrated stable disease (SD) as the best overall response; of these patients, 17 were treated with the targeted recommendation. Nine of 29 patients (31%; 0.153, 0.508 exact 95% confidence intervals) demonstrated progressive disease (PD) as the best overall response, of which seven of these patients were treated with the targeted recommendation. Of the 19 patients with stable disease, 12 were treated with binimetinib (63%; mean days on treatment: 114; range 30–325 days), two with palbociclib (11%; mean days on treatment: 168; range 167–170 days) and five with AAT carboplatin/paclitaxel (26%; mean days on treatment: 84; range 17–125 days [one patient experienced an anaphylactic reaction during the first treatment of AAT and was switched to targeted therapy on day 18]). Of the 12 patients treated with binimetinib, only four were *NRAS*-mutant, while the remaining patients harbored other alterations including concomitant loss of *NF1*, *IGF1R*, and *TP53* with *CCND1* amplification (one patient), *HRAS* point mutation (one patient), concomitant *NF1* and *BRAF* (non-V600) point mutations (one patient), *CCND1* and *CDK6* amplification (one patient), *GNA11* point mutation (three patients), and *GNAQ* point mutation (one patient). For the eight patients whose tumors demonstrated PD as best response, three (38%) received binimetinib, while the remaining patients received sorafenib (1/8), abraxane (2/8) or dacarbazine (2/8). Seven patients who experienced PD after AAT crossed over to receive recommended targeted therapy. Of those patients, one received sunitinib (best response-PD), one received BGJ398 (best response SD-85 days), and five received binimetinib. Two of five patients who received binimetinib after crossover had SD as a best response (mean days on treatment: 106; range 98–114 days) and three had PD. Fifteen of 29 patients whose tumors were treated with binimetinib achieved stable disease (51.7%; mean days on treatment: 113; range 30–325 days). The best overall response rate (BORR) was a 3.4% partial response rate (PR) on the targeted therapy arm. The majority of patients tolerated treatment well with the most common adverse events and serious adverse events listed in S3 and S4 Tables, respectively. Five patients were taken off treatment due to treatment related adverse events (three patients due to grade 3 or 4 events and two patients due to multiple grade 2 events).

### Overview of genomic alterations

Across sequenced specimens, the median estimated tumor purity was 85%. Molecular events that triggered drug matching rules to agents in the trial’s pharmacopeia are summarized in Fig 2. A high level landscape of genomic alterations for each subtype is also shown. Overall, UMs lacked copy number alterations associated with drug rules and, as previously reported, demonstrated *GNA11* and *GNAQ* point mutations with a subset of UMs also demonstrating *BAP1* missense mutations [22, 23]. A larger diversity of drug-associated alterations was observed across the remaining subtypes with *NRAS* single nucleotide variants (SNVs) and *NF1* aberrations occurring across CM, MU, and AM subtypes. With respect to additional key aberrations, *KIT* alterations were found primarily in MUs, with the exception of a missense mutation in one CM, while *TP53* and *PTEN* aberrations were specific to CMs and AMs.

**Fig 3:**
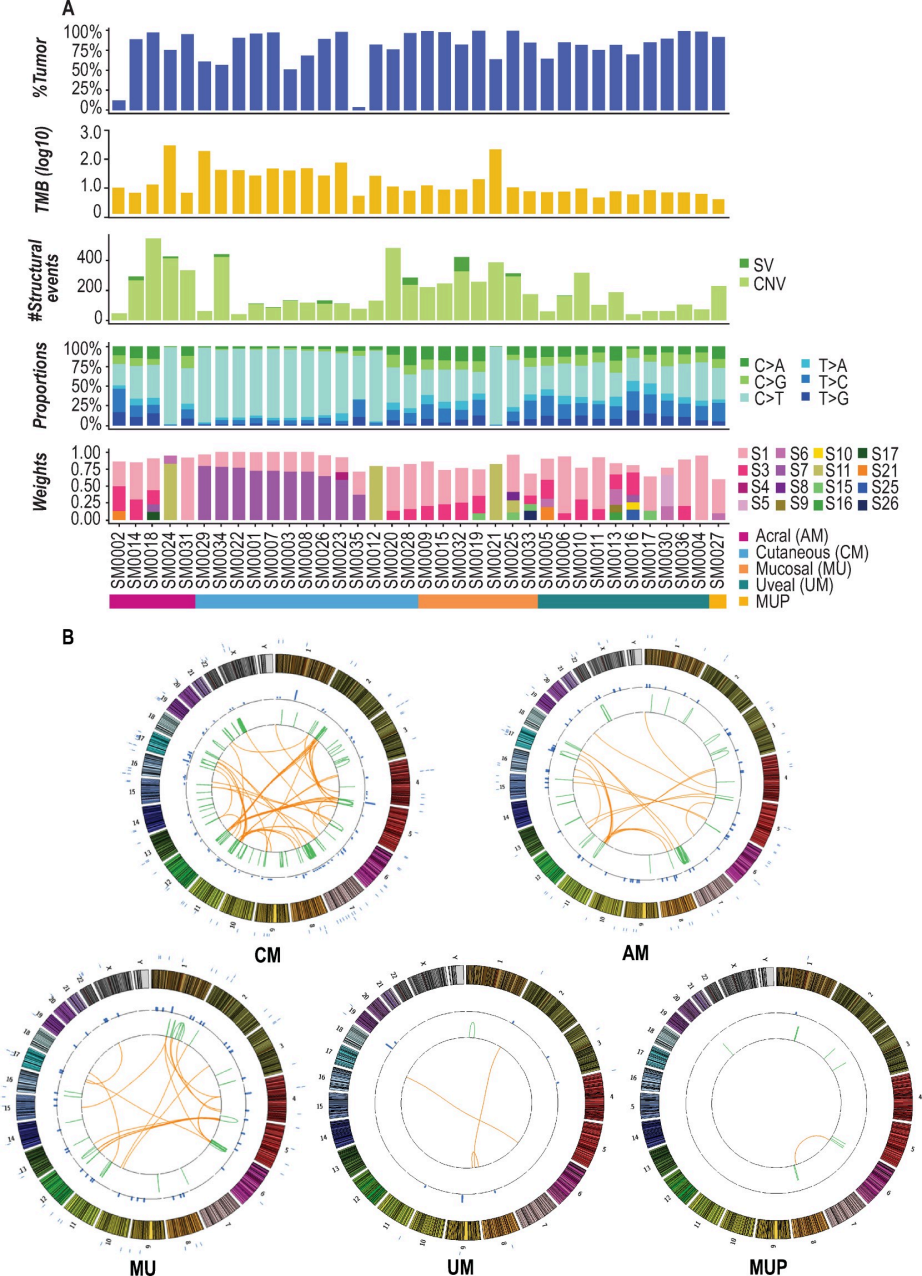
Genomic landscape of trial tumors. A. From top to bottom, estimated tumor purities, TMB (number of mutations per Mb, log10), number of somatic structural variants, breakdown of base substitution changes, and identified Alexandrov somatic signature weights are shown for each patient. B. Subtype-specific Circos plots summarizing identified somatic events are shown. Chromosomes are separated by color and numbered on the outside of each plot. Point mutations are indicated by blue tick marks on the exterior of each plot. Intra-chromosomal SVs are indicated by green lines and inter-chromosomal SVs are indicated by orange lines.

Across the cohort, the median tumor mutational burden was 5.6 mutations/Mb (Figs 2 and 3A; range 3.5–433.3 mutations/Mb). Elevated mutation burdens were observed in three hypermutated tumors of different subtypes, including a MU, although this subtype typically demonstrates low point mutation burden [24] (SM0021 [MU], SM0024 [AM], SM0029 [CM]), with >150 mutations/Mb for each. All these hypermutated tumors demonstrated >14,000 somatic mutations (synonymous and non-synonymous). With respect to structural variants (SVs), 64% of patients did not demonstrate any SVs and a median of 13 SVs were observed across the remaining tumors with the highest number (n = 99) detected in SM0032 (MU). Subtype-specific summaries of identified somatic alterations are shown in Fig 3B.

### Overview of genomic landscape

After completion of the trial, meta-analyses were performed to assess the genomic landscape of patients’ tumors. Across all tumors sequenced, the most prominent base substitution was C>T transitions (Fig 3A), which comprised a median of 41% of all SNVs (synonymous and non-synonymous) in the AMs, 83% in CMs, 39% in MUs, and 37% in UMs. Somatic synonymous and non-synonymous base substitutions for each tumor were further used to identify mutational signatures across samples [25]. The presence of an ultraviolet (UV)-derived signature, as defined by an elevated frequency of C>T transitions in dipyrimidines per Alexandrov signature seven [25], was observed in 33% of patients, encompassing primarily CMs with the exception of one AM and one UM. Additional prominent signatures included Alexandrov signatures one and 11, both of which are governed by a large proportion of C>T substitutions. Signature one, which is associated with increased spontaneous deamination of 5-methylcytosine, has been observed across all cancers, and was present across 92% of patients (33/36). Signature 11, which has been reported to be observed in malignant melanoma [25], was present in 11% of all patients (4/36).

### Functional prediction analysis

Overall, DNA level events demonstrated that recurrently impacted pathways converge on MAPK pathway activation, as expected, and also encompass processes surrounding cell cycle, survival, apoptosis, and telomere maintenance (S1 Fig). To assess the potential cumulative impact of both DNA and RNA level alterations, functional prediction analysis was performed to assess potential loss of function (LOF) or gain of function (GOF) of genes (S2 Fig; see S1 File). *CDKN2A*, for which LOF mutations have been frequently reported in AMs, MUs, and CMs [12, 17], was predicted to have complete LOF in the largest percentage of patients (7/36 [19%]) and partial LOF in 31% of patients (11/36). This finding aligns with consensus copy number variant (CNV) analysis across the entire cohort, which identified *CDKN2A*, as well *CDKN2B*, as falling within significant regions of loss (9p21.3; S3 Fig; S5 Table).

Compared to LOF analysis, GOF analysis revealed a fewer number of impacted genes as governed by CNV gain, SNV or indel, and RNA overexpression. *NRAS* was predicted to have GOF in the greatest percentage of patients (11/36 [31%]) primarily based on activating mutations. Additional genes predicted to demonstrate GOF include *GNAQ* and *GNA11*, which occurred only in UMs. *GAB2* CNV gains have also been reported in non-sun-damaged melanoma, including AMs and MUs, and have been reported to be mutually exclusive of *BRAF*, *NRAS*, and *KIT* aberrations [26]. In our cohort, *GAB2* GOF was predicted based on CNV gains; these patients include one AM (SM0031) and one CM (SM0034), both of which lacked *BRAF*, *NRAS*, and *KIT* alterations, and one MU (SM0021), which demonstrated a *KIT* point mutation with CNV gain.

### Identification of therapeutic vulnerabilities using functional protein signaling pathway activation mapping

To expand upon genomics analyses and identify potential therapeutic vulnerabilities, reverse phase protein array (RPPA) analysis was performed on laser capture microdissected tumor cells from available trial specimens (69%; 25/36) to map the activated signaling pathway architecture of a number of key cancer related signaling pathways including RAF-MEK-ERK signaling. RPPA provided orthogonal validation of genomics analyses with evidence of MAPK activation for 76% (16/21) of patients for whom binimetinib was selected, as well as activation of c-KIT (elevated phosphorylation at Y703) for SM0009 for whom sorafenib was selected as the treatment recommendation based on both a *KIT* point mutation and gain, *KDR* gain, and activation of B-Raf non-V600 (elevated phosphorylation S445) for SM0014 whose tumor demonstrated a *BRAF* fusion and gain and for whom binimetinib was selected as the treatment recommendation. These analyses also revealed activation of pathways, based on elevated levels of phosphorylation events or proteins, associated with tumor features. Significant alterations (P<0.05) were observed in the context of mutation of cell cycle genes, *TERT* gene expression, overall mutational burden, and triple wild-type (WT) status of tumors (Table 2; S6 Table). In the context of cell cycle mutation, affecting 44% of tumors (11/25), Akt-mTor and MEK/ERK signaling, checkpoint signaling, as well as systemic activation of receptor tyrosine kinases were identified. For tumors with elevated *TERT* expression, comprising 24% (6/25) of the cohort, Akt-mTor signaling, and activation of cell cycle and insulin receptor signaling were also identified. Elevated mutation burden, observed in 32% (8/25) of tumors, was associated with systemic activation of HER3 (*ERBB3*) signaling, which may represent a potential therapeutic target for patients who have progressed following immunotherapy and/or whose tumor demonstrates high mutation burden. Lastly, in tumors demonstrating genomic alterations in the MAPK pathway (*NRAS*, *HRAS*, *BRAF*, or *NF1*; 68%; 17/25), activation of Akt-mTor and HER family signaling, RAF activation, STAT signaling, and RTK signaling was also observed. These findings highlight therapeutic vulnerabilities in specific genomic contexts that may span across different histological subtypes.

**Table 2:** Activated pathways and associated drug classes.

Genomic/subtype context	Definition	Associated patients	Activated pathways/proteins (RPPA)	Associated drug classes
Cell cycle mutation	*CDKN2A*, *CCND1*, *CDK4*, and/or *RB1* mutation	SM0001 (CM), SM0012 (CM), SM0014 (AM), SM0015 (MU), SM0018 (AM), SM0019 (MU), SM0021 (MU), SM0023 (CM), SM0024 (AM), SM0025 (MU), SM0027 (MUP)	Akt-mTor signaling	AKT inhibitor; mTor inhibitor; p70S6K inhibitor; PI3K inhibitor
			MEK-ERK signaling	MEK1/2 inhibitor; ERK1/2 inhibitor
			checkpoint signaling (ATM, ATR, CHK1, CHK2 activation)	ATM inhibitor; CHK1 inhibitor; ATR inhibitor
			ALK activation	ALK inhibitor
			HER3 activation	ERBB3 inhibitor; pan-ERBB inhibitor
			EGFR activation	EGFR inhibitor
			RON activation	MET inhibitor; AKT inhibitor; PI3K inhibitor; MEK1/2 inhibitor; ERK1/2 inhibitor
			MET activation	MET inhibitor
*TERT* expression	TPM > 1	SM0007 (CM), SM0014 (AM), SM0019 (MU), SM0021 (MU), SM0023 (CM), SM0024 (AM)	Akt-mTor signaling	AKT inhibitor; mTor inhibitor; p70S6K inhibitor; PI3K inhibitor
			cell cycle signaling	CDK inhibitor
			insulin receptor signaling	IGF1R inhibitor
High TMB	> 2000 somatic mutations/tumor	SM0003 (CM), SM0007 (CM), SM0008 (CM), SM0021 (MU), SM0022 (CM), SM0023 (CM), SM0024 (AM), AM0034 (CM)	ERBB3 signaling	ERBB3 inhibitor; pan-ERBB inhibitor
Non-triple wild-type	*N/HRAS*, *BRAF*, and/or *NF1* mutation	SM0001 (CM), SM0002 (AM), SM0003 (CM), SM0007 (CM), SM0008 (CM), SM0012 (CM), SM0014 (AM), SM0015 (MU), SM0018 (AM), SM0020 (CM), SM0021 (MU), SM0022 (CM), SM0023 (CM), SM0024 (AM), SM0025 (MU), SM0027 (MUP)	HER family signaling	pan-ERBB inhibitor
			Akt-mTor signaling	AKT inhibitor; mTor inhibitor; p70S6K inhibitor; PI3K inhibitor
			RAF	RAF kinase inhibitor; MEK1/2 inhibitor; ERK1/2 inhibitor
			STAT signaling	JAK inhibitor
			RTK (ALK, FMS, ERBB3, EGFR, RON, MET) signaling	ALK inhibitor; FMS inhibitor; ERBB3 inhibitor; pan-ERBB inhibitor; EGFR inhibitor; ERK1/2 inhibitor; JAK inhibitor; mTor inhibitor; MET inhibitor; multi-kinase inhibitor
MU		SM0009, SM0015, SM0019, SM0021, SM0025, SM0032, SM0033	HER family signaling	EGFR inhibitor; ERBB2/3 inhibitor; pan-ERBB inhibitor
			ALK activation	ALK inhibitor
			FAK activation	FAK inhibitor
			Aurora kinase signaling	AURKA inhibitor
			Akt-mTor signaling	AKT inhibitor; mTor inhibitor; p70S6K inhibitor; PI3K inhibitor
			cell cycle signaling	CDK inhibitor^a^
AM		SM0002, SM0014, SM0018, SM0024, SM0031	Akt-mTor signaling	AKT inhibitor; mTor inhibitor; p70S6K inhibitor; PI3K inhibitor
			HER family signaling	EGFR inhibitor; ERBB2/3 inhibitor; pan-ERBB inhibitor
			Heregulin ligand	EGFR inhibitor; ERBB2/3 inhibitor; pan-ERBB inhibitor
			SRC signaling	SRC inhibitor
			DDR signaling	DDR1/2 inhibitor
CM		SM0001, SM0003, SM0007, SM0008, SM0012, SM0020, SM0022, SM0023, SM0026, SM0028, SM0029, SM0034, SM0035	Akt-mTor signaling	AKT inhibitor; mTor inhibitor; p70S6K inhibitor; PI3K inhibitor
			HER family signaling	EGFR inhibitor; ERBB2/3 inhibitor; pan-ERBB inhibitor
UM		SM0005, SM0006, SM0010, SM0011, SM0013, SM0016, SM0017, SM0030, SM0036	^b^	N/A

Genomic contexts and histological subtypes associated with activated pathways identified using RPPA are shown. Drug classes associated with activated pathways are listed.

^a^CDK4/6 inhibitors are contraindicated in the context of RB1 disruption (e.g. SM0015).

^b^UMs were characterized by reduced levels of signaling activation.

Stratification of samples based on histological subtype also highlighted characteristic features (S6 Table). Overall, UMs demonstrated the lowest levels of activation across nearly all analyzed signaling networks. Interestingly, a trend was observed whereby UMs demonstrated lower protein expression of MSH6 (P = 0.04), compared to other subtypes, and this trend occurred concurrently with PD-L1 expression, aligning with the low efficacy of PD-1 blockade that has been described in UMs [27]. In general, MUs were characterized by high levels of signaling activation across multiple pathways, AMs were characterized by activation of AKT-mTor, HER family, heregulin, SRC (SRC proto-oncogene, non-receptor tyrosine kinase) and DNA damage response signaling, and CMs demonstrated modest AKT-mTor and HER family signaling.

Assessment of individual tumors also led to insight into potential therapeutic options. Significantly elevated protein, or phospho-protein, signals (> mean + 2 standard deviations) were observed in specific tumors and point to putative drug targets for pathway inhibition (S7 Table). These include high levels of Alk (SM0025 [MU]), EGFR Y1068 phosphorylation (SM0003 [CM]), ErbB4 (SM0019 [MU]), GSK-3a/b S21/9 phosphorylation (SM0014 [AM], SM0025 [MU]), and PDGFRA Y754 phosphorylation (SM0019 [MU], SM0018 [AM]). In one patient (SM0010 [UM]), RPPA revealed significantly elevated levels of EGFR Y1173 phosphorylation, but genomics analyses of this patient’s tumor did not identify a genomic basis for EGFR activation. Furthermore, within tumors, activation across multiple pathways was also observed. SM0014 (AM), whose tumor demonstrated a *BRAF* fusion, harbored evidence of both MAPK and PI3K pathway activation based on multiple phosphorylation events (BRAF S445, MEK1/2 S217/221, ERK1/2 T202/Y204, GSK3a/b S21/9, p70S6 kinase S371, P70S6 kinase T389, Akt T308). SM0025 (MU) also demonstrated activation of multiple pathways including PI3K/Akt (phosphorylation of Akt S473 and RSK3 T356/S360), Alk, cell cycle (cyclin D1), GSK3A/B (GSK3a/b S21/9 phosphorylation), PTEN (protein level and S380 phosphorylation), and insulin signaling (IRS-1 S612 phosphorylation). Identification of pathway activation in only RPPA data in one patient suggests the utility of identifying novel features using protein pathway activation analysis. Further, activation of multiple pathways within the same tumors highlights the need to consider combination therapies for treatment of *BRAF* ^V600^wt MM.

### Neo-antigen and associated analyses

The utility of using immune checkpoint inhibitors to treat melanoma has been demonstrated to varying degrees in CMs [28, 29], and for AM and MU at a lower frequency [30–32], but extremely limited efficacy has been demonstrated for UMs [33, 34]. As 93% of patients in the G.E.M.M. trial previously received and progressed on immunotherapy (Fig 1; S2 Table), clarifying the utility of immunotherapy for treatment of *BRAF* ^V600^wt MMs is needed. Notably, RPPA revealed detectable expression, as well as elevated expression, of PD-L1, in trial tumors to support reports that PD-L1 expression is not the sole predictor of durable responses to checkpoint inhibition [35, 36]. In CMs, previous studies have also reported an association between mutation and neo-antigen burden and response to immune checkpoint inhibitors [28, 29, 37]. In this trial’s cohort, a strong correlation between the number of predicted neo-antigens that bind class I major histocompatibility complex (MHC) and tumor mutation burden was observed (Pearson’s correlation > 0.99; S4A and S3B Figs). Despite this correlation, the lack of a response to immune checkpoint inhibitors in this trial’s cohort stresses the need to better understand how a patient’s immune system may influence outcome following treatment with immune checkpoint inhibitors.

To investigate if individual tumors may be better or less able to evade the patient’s immune system, we evaluated neo-antigen binding predictions between somatic mutations in each patient’s tumor and his or her own MHC alleles, as compared against the entire cohort in both trial and available archival specimens. Given that more highly expressed mutations may be more influential as neo-antigens, we also assessed the impact of incorporating RNA expression. Overall, trends toward selection against strong binding neo-antigens, for which mutation selection may have played a role in immune evasion, were observed in eight to 14% of patients (S8 Table; see S1 File). Inclusion of RNA expression data led to the identification of ten patients (28%), whose tumors spanned histological subtypes (3/5 AMs, 3/10 UMs, 3/13 CMs, 1/7 MUs), and who demonstrated significant evidence of selection (S8 Table). As all patients who received immunotherapy prior to the trial progressed, results from this analysis indicate that other factors besides neo-antigens, such as patients’ microbiomes, are contributing to response to treatment, as well as immune surveillance.

Studies have also reported specific genomic alterations that may be associated with immune recognition escape or immunotherapy response [38–40]. We thus interrogated trial data to assess implicated genes, including *JAK1* (Janus kinase 1), *JAK2*, *B2M*, *PBRM1*, *IFNGR1*, and *STAT1* (S9 Table) [38–40]. Seven patients (19%) demonstrated somatic events in at least one of these genes. Deficiencies in mismatch repair (MMR) have also been reported to be correlated with response to immune checkpoint inhibitors [41–43]. Two tumors with elevated mutation burden (>150 mutations/Mb), and which were hypermutated, harbored somatic *MSH6* missense mutations (S9 Table). The functional impact of the variants is not known but one (G409E) falls within the MutS DNA binding domain and may thus affect DNA repair. The remaining tumors did not harbor any *MSH6* mutations. Missense mutations in other MMR genes (*MLH1*, *MSH2*, *PMS2*) were identified across four tumors, however these may represent passenger events. Acknowledging limitations in sample size for this study, different factors including potential DNA repair deficiencies, specific genomic events, and low mutation selection, may be associated with lack of response to immune checkpoint inhibitors.

## Discussion

Identifying strategies to optimize treatment selection and efficacy for patients with *BRAF* ^V600^wt MM remains an unmet need. In the SU2C G.E.M.M. trial, comprehensive interrogation of the whole exome and transcriptome, in combination with a specific pharmacopeia, did not outperform physician’s choice for treatment of *BRAF* ^V600^wt MM patients. However, the trial only allowed monotherapy drug selection from the limited pharmacopeia accessible at the time of the study. Furthermore, a number of other drugs which more effectively target genomic alterations seen in this trial were early in development and not approved. Based on genomic profiling and due to activating *NRAS* mutations, binimetinib was predominantly selected as the treatment strategy. As all patients who previously received immunotherapy had progressed prior to the trial and as only one patient demonstrated a PR following the trial, results from this study highlights opportunities to identify improved treatment options for *BRAF* ^V600^wt patients. Following completion of patient enrollment for this trial, Dummer et al. [44] reported that binimetinib improves PFS, compared to dacarbazine, in *NRAS*-mutant melanoma patients who previously failed immunotherapy. This finding provides evidence of the utility of binimetinib for this specific patient population, noting that in our G.E.M.M. trial, 58% (7/12) of *NRAS*-mutant patients received binimetinib, which included the study’s one PR, as well as SD (n = 4) and PD (n = 2) responses.

Post-hoc analyses were performed to assess the genomic and proteomic landscape of the trial’s cohort with the goal of gaining insight into how future trials and treatments may be improved for *BRAF* ^V600^wt MM patients. As expected, meta-analyses align with previously reported findings across the various histological subtypes. However, by utilizing a multi-omic based approach to further characterize tumor biology and to uncover additional treatment options, potential therapeutic vulnerabilities were identified. Activation of key cancer pathways was found to be associated with specific genomic contexts including mutation of cell cycle genes, *TERT* expression, tumor mutation burden, and non-triple wt status of tumors and thus points to the need for development of novel drug classes to potentially inhibit activated pathways. Activation of multiple pathways was also observed in individual tumors to further highlight the need for combination therapies, which, for example, has been effective for inhibiting MAPK and mTor signaling through the use of the combination of trametinib and ceritinib in *in vitro* and *in vivo* analyses [45]. RPPA also identified one patient who demonstrated EGFR activation, a feature that was not apparent based on genomics analyses, to illustrate the possible utility of integrating phosphoprotein-based RPPA analyses to help guide treatment selection.

In an effort to shed light into potential resistance mechanisms to immunotherapy in patients prior to the trial, we further assessed neo-antigens and genes that have been reported to be associated with response to immunotherapy. While these analyses are not sufficiently powered, only a subset of patients demonstrated evidence of tumor selection and thereby illustrates that additional factors are contributing to response, as others have described [35, 36]. While further investigations are needed to better understand the molecular context associated with effective use of immunotherapy, we highlight additional opportunities for developing novel targeted therapies, as well as combination treatments, for *BRAF* ^V600^wt MM patients.

A number of caveats associated with the trial and post-trial analyses were apparent. Firstly, patient referral patterns for the trial were influenced by the availability of targeted treatment studies at the enrolling institutions. Furthermore, the trial enriched for CMs that progressed on previous treatments. As a result, this trial population is a subset of the real world melanoma population, a deficiency common to many other clinical trials. Based on the patient’s tumor molecular profile, combination treatment recommendations were discussed in many if not all cases. The majority of MTB recommendations involved treatment combinations. However, due to lack of sufficient safety data, no combination therapies were allowed to be administered and, as such, only the primary single agent recommendation was used. A recurrent theme during MTBs was also an interest in recommending the combination of MEK and CDK4/6 inhibitors, however, lack of sufficient safety data during the trial led to utilization of monotherapy binimetinib, which previously demonstrated a 15–20% PR rate in patients with *NRAS* disease [19]. Of note, while the effectiveness of combined MEK and CDK4/6 inhibitors has been demonstrated to some extent [46–49] in both pre-clinically and clinically in *NRAS* codon 61 mutant melanoma, significant toxicities from this regimen have clinically been described [46]. Development of resistance to the combination has also been reported, but strategies to overcome resistance are being explored [50, 51].

Results from this trial emphasize the critical need for drug development of novel targeted agents and drug combinations in order to target specific pathways within specific genomic contexts. Although all patients in this trial who previously received immunotherapy progressed, the limited selection observed in this trial’s cohort based on neo-epitope analyses highlights the need to better understand how immunotherapies may be personalized for individual patients or subsets of patients. Further, given the short turnaround times between biopsy and the MTB, as well as biopsy and treatment, along with the outcomes from the trial, performing testing at initial diagnosis may also be considered for future trials. Our findings thus illustrate the heterogeneity of MM and the complexity of optimizing treatment selection, and highlight opportunities to include combination treatment strategies and specify treatment options associated with defined genomic contexts in future trials. As drug development evolves, we believe this model of G.E.M.M. will ultimately demonstrate its value. Taking advantage of the opportunities this molecular approach provides will be necessary to identify and design more effective and durable treatment options for *BRAF* ^V600^wt MM patients.

## Materials and methods

Methods for the pilot clinical trial were previously described [18]. Please see additional details in the S1 File. The full trial protocol is available under Supporting information.

### Patient enrollment/consent

Forty-nine MM patients were consented between June 2014 and December 2016 and enrolled onto this Phase II, prospective, multi-center, open-label study (ClinicalTrials.gov Identifier NCT02094872) from across seven sites including the Karmanos Cancer Institute, Mayo Clinic (Scottsdale, Rochester, and Jacksonville), University of Michigan Comprehensive Cancer Center, Charles A. Sammon Cancer Center/Baylor University Medical Center, and Yale Cancer Center. All patients who had received prior treatments experienced disease progression prior to enrolling onto this trial. Ethics review boards at all participating institutions approved the study, which was conducted in accordance with the Declaration of Helsinki and Good Clinical Practice guidelines. The Western Institutional Review Board (Puyallup, Washington) was the overall IRB of record for this study and approved all protocol and consent forms. All patients provided written informed consent. All data and information were de-identified such that individual patients could not be identified during or after data collection.

Inclusion criteria included patients aged ≥18 years with metastatic or locally advanced and unresectable *BRAF*wt melanoma who had either progressed following previous treatment with radiation therapy (RT), investigational agents, and/or immunotherapy including ipilimumab, nivolumab, pembrolizumab, and/or interleukin-2 were eligible to participate in this study. A 28 day or 5 half-life washout, whichever was shorter was required with recovery to ≤grade 1 toxicity from prior therapy. Prior therapy with any MEKi was not allowed. Other criteria included life expectancy ≥3 months; tumor accessible by interventional radiology or surgical intervention; measurable disease as defined by RECIST (Response Evaluation Criteria in Solid Tumors) v1.1 criteria; Eastern Cooperative Oncology Group performance status of ≤2; ability to tolerate oral medication; and adequate organ and marrow function, Aspartate aminotransferase (AST), alanine aminotransferase (ALT) and alkaline phosphatase ≤5 x upper limit of normal (ULN) was allowed if liver metastases were present, Alanine aminotransferase (ALT) ≤ 2.5 x upper limit of normal (ULN) or ≤ 5 x ULN if liver metastases were present. Creatinine ≤ 1.5 x ULN or creatinine clearance ≥ 50 mL/min was required. Patients were excluded if they had prior cytotoxic chemotherapy treatment for metastatic melanoma. Brain metastases were allowed if stable for >1 month after treatment. Additional exclusion criteria were typical of a Phase II trial in this patient population.

### Trial study design

This Simon two-stage optimal design trial [52] enrolled patients with relapsed BRAF^V600^*wt* MM. Pre-treatment tumor specimens underwent next generation sequencing and gene expression profiling identifying mutations, inserts and deletions, and copy number variations. Based on this data, a treatment plan was formulated by both a molecular and clinical tumor board and reviewed by an independent medical overseer. Initial disease assessment was done at 30–35 days, with subsequent assessment done at eight or nine-week intervals (depending on length of cycle) with treatment end dates ranging from July 2014 through August 2017. The Simon two-stage optimal design enrolled 20 response-evaluable binimetinib patients and was to terminate early if one or fewer patients responded (1/20 = 5%). The trial was powered for a 20% response rate. If there were two or more binimetinib patients who would have responded, then an additional 25 patients (total 47) would have been enrolled in the second stage of the Simon two-stage design.

### Sample collection

For each biopsy, two 1–2 centimeter 16- or 18-gauge core needle specimens from accessible tumor were collected and flash frozen in liquid nitrogen. Fresh frozen samples were stored at -80°C until shipping. Frozen samples were shipped on dry ice for DNA and RNA extraction. Ten to 20 mL of whole blood was collected at the time of initial biopsy in EDTA tubes for constitutional DNA extraction. Tumor DNA and RNA were respectively extracted from each of the two frozen cores using the Qiagen AllPrep Kit. Constitutional DNA was extracted using the Qiagen Gentra Puregene Blood Kit. DNA and RNA quantitation and purity was assessed by spectrophotometry. RNA integrity was evaluated using the Agilent TapeStation.

### Treatment outcomes

Outcomes are defined as–(1) complete response (CR): disappearance of all target lesions. Any pathological lymph nodes (whether target or non-target) must have reduction in short axis to less than ten mm; (2) PR: At least a 30% decrease in the sum of the diameters of target lesions, using the baseline sum diameters as reference; (3) PD: At least a 20% increase in the sum of the diameters of target lesions, using the smallest sum on study as reference (this includes the baseline sum if that is the smallest on study). In addition to the relative increase of 20%, the sum must also demonstrate an absolute increase of at least five mm (the appearance of one or more new lesions is also considered progressions); (4) SD: Neither sufficient shrinkage to qualify for PR nor sufficient increase to qualify for PD, using the smallest sum diameters while on study as reference.

### Statistical analyses

Statistical analysis was conducted in R [53] and the binom.test package was used to determine exact binomial confidence intervals. The primary endpoint is BORR to therapy. For overall response, patients were followed to disease progression or until the patient came off study. Only patients receiving binimetinib were included in the primary analysis. The primary statistical hypothesis was to compare BORR in patients receiving binimetinib to a historical control response rate using a Simon two-stage design. The historical response rate is <10% [54–58] and a historical response rate of 7% was used as the null hypothesis. The statistical significance level of this trial’s design is 0.1. If the response rate was > = 20%, this design has a power of 90%. Overall survival, PFS, and response rates of all patients, and separately for those treated with binimetinib, were also examined. The trial was not powered for these endpoints and no corrections were made for the multiplicity of these tests.

### Clinical trial data processing

For the clinical trial, the definition of the pharmacopeia utilized for the study, drug-gene matching rules, and procedures for data analysis were previously described [18].

### Next generation sequencing

In summary, tumor and constitutional DNA samples (A260/A280 ratio of 1.8–2.0) were subjected to library construction using > = 150ng inputs, Kapa Biosystems’ Kapa Hyper Prep Kit, and utilizing a custom Agilent SureSelect target enrichment system that contains probes targeting the whole exome, along with genome-wide copy number probes, and probes targeting known cancer translocations. For RNAseq (RNA sequencing) library construction, 500ng inputs were used with an RNA RIN (RNA Integrity Number) of > = 7. Illumina’s TruSeq RNA Sample Preparation V2 Kit was used to construct libraries. All libraries were sequenced on the Illumina HiSeq2500 for 2x100 reads. Constitutional data was used solely for the identification of true somatic alterations and no data was returned back to patients and patients’ families. The median sequencing coverage was 410X for tumors and 205X for the normal samples, and a median of 235 million paired and mapped RNA reads were generated across all patients.

### Molecular and clinical tumor boards

Separate molecular and clinical tumor boards were held for each patient as previously described [18]. For the molecular boards, the minimum quorum included at least one genomics expert or bioinformatician, one pharmacy representative, one patient advocate, and three clinical investigators. For each patient, data and information discussed at each molecular tumor board was passed on to a clinical tumor board to outline a personalized treatment plan. The minimum quorum for the clinical board included at least three clinical investigators, one patient advocate, and a pharmacy representative. Each molecular report was comprised of sequencing statistics, a summary Circos plot, and two levels of alphabetically ordered molecular alterations according to the following definitions: Level 1, a molecular aberration associated with a specific drug per published literature; and Level 2, a molecular aberration thought to be associated with cancer per available information (TCGA [The Cancer Genome Atlas], Sanger Cosmic) although published literature did not link the specific alteration with a drug. Each report conveyed the predicted efficacy of the drugs identified by each of the analytical methods and also highlighted evidence that supported or refuted the use of the predicted drug in the context of the patient’s disease state.

## Supporting information

S1 Checklist CONSORT 2010 checklist of information to include when reporting a randomised trial*. (DOC)

S1 File (DOCX)

S1 Table Study pharmacopeia. (XLSX)

S2 Table Additional patient clinical data. (XLSX)

S3 Table Most common adverse events. (XLSX)

S4 Table Reported serious adverse events. (XLSX)

S5 Table Consensus regions of copy gains and losses. (XLSX)

S6 Table Reverse phase protein array (RPPA) analysis. (XLSX)

S7 Table Patient-specific RPPA data. (XLSX)

S8 Table Neo-antigen binding prediction analyses. (XLSX)

S9 Table Selected somatic alterations. (XLSX)

S1 Fig Recurrently impacted pathways in trial patients. Recurrently impacted pathways in melanoma are summarized here in the context of somatic alterations observed across trial patients. The type of alteration is shown (amplification, deletion, mutation) and the percentage of patients of each subtype that demonstrate alterations in specific genes are shown and color-coded. The legend on the bottom right lists the total number of patients for each subtype.
(PNG)

S2 Fig Gene functional status prediction analysis. A: Loss of function (LOF) analysis—Predicted genes experiencing LOF are shown based on integration of DNA and RNA data. Each column represents an individual patient tumor. The central plot lists genes (Y-axis) demonstrating partial or complete LOF in each patient. The Loss of Function score plot indicates the proportion of all patients demonstrating a predicted partial or complete LOF. The right-most plot shows the breakdown of variants supporting a complete LOF only. B: Gain of function (GOF) analysis—Predicted genes experiencing GOF are shown based on integration of DNA and RNA data. Each column represents an individual patient tumor. The central plot lists genes (Y-axis) demonstrating GOF in each patient. The variant type plot indicates the proportion of all patients demonstrating a predicted GOF. Unlike the LOF predictions, a GOF prediction does not differentiate between partial and complete gain (see S1 File).
(ZIP)

S3 Fig Consensus copy number alterations. Consensus regions of gains (left, red) and losses (right, blue) across the entire cohort are shown. Green arrows mark significant regions (Q<0.05). Q-values are shown on the lower x-axis (Benjamini & Hochberg FDR), G-scores are shown on the upper x-axis, and chromosome numbers are labeled along the y-axis.
(PDF)

S4 Fig Neo-antigen analysis of tumors. A: HLA-A, B, and C expression for each patient is shown on the left plot. Predicted neo-antigen expression, along with the predicted binding of HLA-A, B, or C to the neo-antigen, is shown on the right plot. B: Neo-antigen counts plotted against mutation burden (mutations per Mb) revealed a trend towards significance based on a Pearson’s correlation.
(ZIP)

S1 Protocol (PDF)
