# Supplementary material for: Identifying treatment options for BRAFV600 wild-type metastatic melanoma: A SU2C/MRA genomics-enabled clinical trial
Source: PLoS One. 2021 Apr 7;16(4):e0248097. doi: 10.1371/journal.pone.0248097 (PMC8026051; doi:10.1371/journal.pone.0248097)
Supplement: S1 File — (DOCX) [file pone.0248097.s002.docx]

S1 File.

*Post-trial meta-analyses*

After completion of the trial, data was interrogated against a knowledgebase of predictive genomic oncology biomarkers developed at Ashion which enabled a comprehensive clinically oriented analysis of actionable cancer specific alterations. Clinical trials were restricted to trials that were recruiting patients in the US and excluded drug classes that were included in the original trial and agents that were in Phase I trials and thus did not have Phase II dosing.

For cohort meta-analyses, data processing and analysis was performed using a computational framework on our high-performance computing system that enables integrated genomic and transcriptomic analysis of paired tumor/normal exome and tumor RNAseq data (*1*) using TGen’s Jetstream pipeline. Raw BCL files were converted to FASTQ files using Illumina’s BCLConverter tool. DNA FASTQs were aligned to the human reference genome (build 37) using BWA-MEM (bwa v0.7.8) (*2*) and RNA FASTQs were aligned using STAR 2.3.1z (*3*). For DNA data, base quality scores were recalibrated using GATK v3.1-1 (*4*), and duplicates were marked using Picard v1.111 (http://broadinstitute.github.io/picard/) to generate BAMs, on which joint indel realignment was performed using GATK v3.1-1. Final BAMs were used for identification of somatic mutations (point mutations, insertions, deletions), structural changes, and CNVs. Variant calling was performed using Seurat (*5*) (quality score>30), Strelka (*6*), and MuTect (*7*), and annotated using GENCODE version 3 by Ensembl and build 37.1. No quality scores were generated by MuTect and Strelka. Final somatic SNVs were called by at least 2/3 callers.

DELLY v0.7.6 (*8*) was used to identify structural variants in tumor samples. The DELLY filter module was run with the following options: altFrac=0.1, ratioGeno=0.75, coverage=5, controlContamination=0, minSize=500, maxSize=500000000. For specific structural variant types we set alternate options as follows: minSize= 2000 for DEL, minSize=100 for DUP, minSize=5 and maxSize=87 for INS, and minSize=100 for INV. DELLY’s filtered BCF files were converted to VCF files using BCFTOOLS from the SAMTOOLS v1.2-172-g06ce5a1 distribution. Calls annotated as PASS are retained.

CNV and LOH (loss of heterozygosity) detection was performed using TGen’s internally developed copy number tool tCoNut (<https://github.com/tgen/tCoNuT>). tCoNuT is a read-depth based CN tool that uses heterozygous SNPs from germline samples to identify regions of diploidy in tumor samples for centering log2 fold change values.

DeconstructSig (*9*) was used to identify mutational processes in each tumor using all SNVs (synonymous and non-synonymous) and as well as those occurring within each melanoma subtype. Figure 2A was constructed using signatures with weights > 0.1. Consensus somatic CNVs were identified using GISTIC2.0 (Genomic Identification of Significant Targets in Cancer) (*10*).

Gene functional status analysis was performed using both DNA and RNA sequencing data to predict the cumulative effect of identified somatic and germline alterations as genes impacted by LOF or GOF. For LOF analysis, these events were integrated to predict partial, or complete, loss of function of a gene; complete loss is defined as predicted loss of all functional copies of a gene, while partial loss is defined as the presence of evidence of loss, however lacking evidence of complete loss of function. For GOF analysis, a prediction is made with respect to activation, or GOF, of a gene. This analysis considered somatic aberrations including SNVs and indels, CNVs, structural variants (SVs; inversions, deletions, translocations), loss of heterozygosity (LOH), germline variants, and RNA expression of DNA changes. For GOF analysis, requirements include: (1) gene must have a recurrent, clustered, or nominated mutation and a TPM>1, (2) gene must demonstrate at least a four copy gain with a log2 fold >= 1.5, and (3) to be considered overexpressed, at least one other patient in the cohort must demonstrate a somatic SV impacting the gene (if the gene falls directly 3’ or 5’ to an SV, this also fulfills the requirement). Recurrent mutations are defined as non-synonymous SNVs or indels that occur within the same gene at the same amino acid position and that are present in more than one patient in the cohort. Clustered mutations are those that require the presence of greater than one recurrent mutation and at least five total SNVs that fall within 10% of CDS space across the entire cohort. Nominated genes are those that contain a recurrent mutation; other mutations in the gene that do not cluster or are not recurrent are passed as GOF if the gene is nominated by >1% of the cohort through alternative events, including other recurrent mutations in the gene, copy number gain, a structural variant with over-expression, or genomically validated RNA fusions. Overexpression of genes is evaluated separately for each tumor—TPMs of all genes in each tumor were normalized to the median absolute deviation and genes whose TPM falls within the upper quartile are considered overexpressed. For presentation, the GOF plot lists genes that are impacted across a minimum of three patients, while the LOF plot lists genes that are impacted across a minimum of four patients.

*Neo-antigen analysis*

For neo-antigen analyses, we compared: (1) binding of each patient’s mutations to his/her own MHC alleles compared to the other patients’ MHC alleles (swapped HLA analysis); as well as (2) binding of each patient’s own mutations to the mutations of the other patients in the cohort to that patient’s MHC alleles (swapped mutation analysis).

We first predicted the number of neo-antigens generated based on somatic mutations. BWA’s HLA caller (bwa-0.7.11), seq2HLA 2.2, and phlat (*11*) were used to generate six digit HLA calls for the HLA-A, B, and C genes. Analysis of HLA expression was also performed using seq2HLA 2.2 (*12*). Mutated protein sequences were generated using varcode (*13*) based on somatic non-synonymous mutations, frameshifts, codon insertions, and codon deletions called by two of three callers. NetMHCpan 4.0 (*14*) was used as the prediction method, with peptide lengths of eight to eleven and tiling across every somatic mutation identified, and as well as across the corresponding wildtype peptide against every HLA genotype in the cohort. In order to collapse binding predictions to one number per mutation/patient, PHBR (patient harmonic mean best rank) scores were calculated (*15*). For PHBR, the lowest percent rank was found for each mutation/HLA pair out of all tiling peptides and lengths, and for each patient, the harmonic mean of the six HLA genotypes best percent rank were determined for each calculation. If there were discordant HLA genotype calls across samples/callers, the percent rank was weighted by the number of times the genotype was called. For swapped HLA analyses, the proportion of PHBR scores better than defined cutoffs (strong binders: PHBR<0.5, weak binders: PHBR<2 (*15*)) was compared for each patient’s mutation for the patient’s own HLA to all other patients’ HLA. For swapped mutation analyses, the proportion of PHBR or DAI scores better than the cutoff described above were compared for each patient’s mutation against all other patients’ mutations to the patient’s own HLA. Receiver operator curve (ROC) analysis was used to determine if the matched predicted binding was more likely to be worse than the swapped predicted binding, indicating selection against neo-antigen generating mutations. In the ROC analysis, the PHBR scores were used as the predictor and whether the score came from the matched or swapped analysis was used as the class label. In the swapped HLA analysis, the area under the curve (AUC) indicates the probability that the patient’s mutations are worse binders to their own MHC alleles than to other patients’ MHC alleles. In the swapped mutation analysis, the AUC indicates the probability that the patient’s mutation generates worse binding peptides than another patient’s mutation to that patient’s MHC alleles. In both analyses, bootstrapping was used to calculate confidence intervals on the AUC using the percentile method and 100 bootstrapping iterations (Matlab 2018a). In order to take into account that more highly expressed mutations may be more influential as neo-antigens, we also repeated the swapped HLA and swapped mutation analyses, but weighted the binding scores by RNA alternate allele counts. We used the square root of the RNA alternate allele count as was used in the PGV-001 trial (*13*) after adding a pseudocount of 0.1 to include mutations with RNA alternative allele count was observed to be zero. In all four analyses, some patients showed trends toward selection against strong neo-antigens (AUC [area under curve] > 0.5). Significant evidence of selection is indicated with the lower boundary of the 95% confidence interval on the AUC exceeding 0.5 (Supplementary Table 4).

*RNAseq analysis*

For identified somatic mutations, RNA allele frequencies were computed using Varscan (*16*). For fusion detection, TopHat2 v2.0.8b (*17*) was used to align data and TopHat-Fusion (*18*) was used to identify events. For single sample gene set enrichment analysis (ssGSEA), RNA data was quantified using Salmon v0.7.2 in the form of TPMs (*19*). ssGSEA (*20, 21*) is a dimensionality reduction method that computes gene enrichments scores of individual sample and gene sets, as defined by pathways, to provide a meaningful interpretation of the data. We utilized ssGSEA to project gene expression values into the Broad hallmark gene set (*22*). Using genes predicted to demonstrate LOF or GOF, predicted pathways were generated through the use of IPA v01-13 (QIAGEN Inc., <https://www.qiagenbioinformatics.com/products/ingenuity-pathway-analysis>) (*23*).

*RPPA analysis*

Protein pathway activation mapping of clinical tissue samples was performed using Laser Capture Microdissection (LCM) coupled to Reverse Phase Protein Array (LCM-RPPA) analysis as previously described (*24*). The use of LCM to obtain enriched tumor epithelium prior to RPPA based protein and phosphoprotein measurements was found to be essential for producing accurate data from clinical tissue specimens (*25*). Using this workflow, quantitative measurement of the levels and activation state of 109 key signaling proteins and phosphoprotein drug targets known to be involved in key cancer pathways such as motility, growth, apoptosis, autophagy, inflammation, and survival, were measured. Category/population based statistical comparisons were performed using parametric (t-test) or non-parametric (Wilcoxon rank sum) based testing depending on data distribution. P-values were unadjusted and set for significance at p<0.05. All antibodies used for RPPA were extensively pre-validated as previously described (*25*). Evidence of MAPK activation is defined as having at least one measurement associated with MAPK activation (protein or phospho-protein level) whose value is greater than the mean across all 25 analyzed tumors. Feature analysis parameters are as follows: cell cycle mutation is defined as mutation of cell cycle genes including *CDKN2A, CCND1, CDK4, CDK6*, and *RB1*; high mutation burden is defined as >2,000 somatic mutations (synonymous and non-synonymous); elevated *TERT* gene expression is defined as >1 TPM; triple wild-type status is defined as mutation in *NRAS* or *HRAS*, *BRAF*, and *NF1* genes. No *KRAS* mutations were identified.

1. S. Nasser *et al.*, An integrated framework for reporting clinically relevant biomarkers from paired tumor/normal genomic and transcriptomic sequencing data in support of clinical trials in personalized medicine. *Pac Symp Biocomput*, 56-67 (2015).

2. H. Li, R. Durbin, Fast and accurate short read alignment with Burrows-Wheeler transform. *Bioinformatics.* **25**, 1754-1760. doi: 1710.1093/bioinformatics/btp1324. Epub 2009 May 1718. (2009).

3. A. Dobin *et al.*, STAR: ultrafast universal RNA-seq aligner. *Bioinformatics.* **29**, 15-21. doi: 10.1093/bioinformatics/bts1635. Epub 2012 Oct 1025. (2013).

4. A. McKenna *et al.*, The Genome Analysis Toolkit: a MapReduce framework for analyzing next-generation DNA sequencing data. *Genome Res.* **20**, 1297-1303. doi: 1210.1101/gr.107524.107110. Epub 102010 Jul 107519. (2010).

5. A. Christoforides *et al.*, Identification of somatic mutations in cancer through Bayesian-based analysis of sequenced genome pairs. *BMC Genomics.* **14:302.**, 10.1186/1471-2164-1114-1302. (2013).

6. C. T. Saunders *et al.*, Strelka: accurate somatic small-variant calling from sequenced tumor-normal sample pairs. *Bioinformatics.* **28**, 1811-1817. doi: 1810.1093/bioinformatics/bts1271. Epub 2012 May 1810. (2012).

7. K. Cibulskis *et al.*, Sensitive detection of somatic point mutations in impure and heterogeneous cancer samples. *Nat Biotechnol.* **31**, 213-219. doi: 210.1038/nbt.2514. Epub 2013 Feb 1010. (2013).

8. T. Rausch *et al.*, DELLY: structural variant discovery by integrated paired-end and split-read analysis. *Bioinformatics.* **28**, i333-i339. doi: 310.1093/bioinformatics/bts1378. (2012).

9. R. Rosenthal, N. McGranahan, J. Herrero, B. S. Taylor, C. Swanton, DeconstructSigs: delineating mutational processes in single tumors distinguishes DNA repair deficiencies and patterns of carcinoma evolution. *Genome Biol.* **17:31.**, 10.1186/s13059-13016-10893-13054. (2016).

10. C. H. Mermel *et al.*, GISTIC2.0 facilitates sensitive and confident localization of the targets of focal somatic copy-number alteration in human cancers. *Genome Biol.* **12**, R41. doi: 10.1186/gb-2011-1112-1184-r1141. Epub 2011 Apr 1128. (2011).

11. Y. Bai, M. Ni, B. Cooper, Y. Wei, W. Fury, Inference of high resolution HLA types using genome-wide RNA or DNA sequencing reads. *BMC Genomics.* **15:325.**, 10.1186/1471-2164-1115-1325. (2014).

12. S. Boegel *et al.*, HLA typing from RNA-Seq sequence reads. *Genome Med.* **4**, 102. doi: 110.1186/gm1403. eCollection 2012. (2012).

13. A. Rubinsteyn *et al.*, Computational Pipeline for the PGV-001 Neoantigen Vaccine Trial. *Front Immunol.* **8:1807.**, 10.3389/fimmu.2017.01807. eCollection 02017. (2018).

14. M. Nielsen, M. Andreatta, NetMHCpan-3.0; improved prediction of binding to MHC class I molecules integrating information from multiple receptor and peptide length datasets. *Genome Med.* **8**, 33. doi: 10.1186/s13073-13016-10288-x. (2016).

15. R. Marty *et al.*, MHC-I Genotype Restricts the Oncogenic Mutational Landscape. *Cell.* **171**, 1272-1283.e1215. doi: 1210.1016/j.cell.2017.1209.1050. Epub 2017 Oct 1226. (2017).

16. D. C. Koboldt *et al.*, VarScan: variant detection in massively parallel sequencing of individual and pooled samples. *Bioinformatics.* **25**, 2283-2285. doi: 2210.1093/bioinformatics/btp2373. Epub 2009 Jun 2219. (2009).

17. D. Kim *et al.*, TopHat2: accurate alignment of transcriptomes in the presence of insertions, deletions and gene fusions. *Genome Biol.* **14**, R36. doi: 10.1186/gb-2013-1114-1184-r1136. (2013).

18. D. Kim, S. L. Salzberg, TopHat-Fusion: an algorithm for discovery of novel fusion transcripts. *Genome Biol.* **12**, R72. doi: 10.1186/gb-2011-1112-1188-r1172. (2011).

19. R. Patro, G. Duggal, M. I. Love, R. A. Irizarry, C. Kingsford, Salmon provides fast and bias-aware quantification of transcript expression. *Nat Methods.* **14**, 417-419. doi: 410.1038/nmeth.4197. Epub 2017 Mar 1036. (2017).

20. D. A. Barbie *et al.*, Systematic RNA interference reveals that oncogenic KRAS-driven cancers require TBK1. *Nature.* **462**, 108-112. doi: 110.1038/nature08460. Epub 02009 Oct 08421. (2009).

21. A. Subramanian *et al.*, Gene set enrichment analysis: a knowledge-based approach for interpreting genome-wide expression profiles. *Proc Natl Acad Sci U S A.* **102**, 15545-15550. Epub 12005 Sep 15530. (2005).

22. A. Liberzon *et al.*, The Molecular Signatures Database (MSigDB) hallmark gene set collection. *Cell Syst.* **1**, 417-425. (2015).

23. A. Kramer, J. Green, J. Pollard, Jr., S. Tugendreich, Causal analysis approaches in Ingenuity Pathway Analysis. *Bioinformatics.* **30**, 523-530. doi: 510.1093/bioinformatics/btt1703. Epub 2013 Dec 1013. (2014).

24. J. D. Wulfkuhle *et al.*, Evaluation of the HER/PI3K/AKT Family Signaling Network as a Predictive Biomarker of Pathologic Complete Response for Patients With Breast Cancer Treated With Neratinib in the I-SPY 2 TRIAL. *JCO Precision Oncology*, 1-20 (2018).

25. E. Baldelli *et al.*, Impact of upfront cellular enrichment by laser capture microdissection on protein and phosphoprotein drug target signaling activation measurements in human lung cancer: Implications for personalized medicine. *Proteomics Clin Appl.* **9**, 928-937. doi: 910.1002/prca.201400056. Epub 201402015 Mar 201400024. (2015).
