# Supplementary figures and images for: Identifying treatment options for BRAFV600 wild-type metastatic melanoma: A SU2C/MRA genomics-enabled clinical trial
Source: PLoS One. 2021 Apr 7;16(4):e0248097. doi: 10.1371/journal.pone.0248097 (PMC8026051; doi:10.1371/journal.pone.0248097)

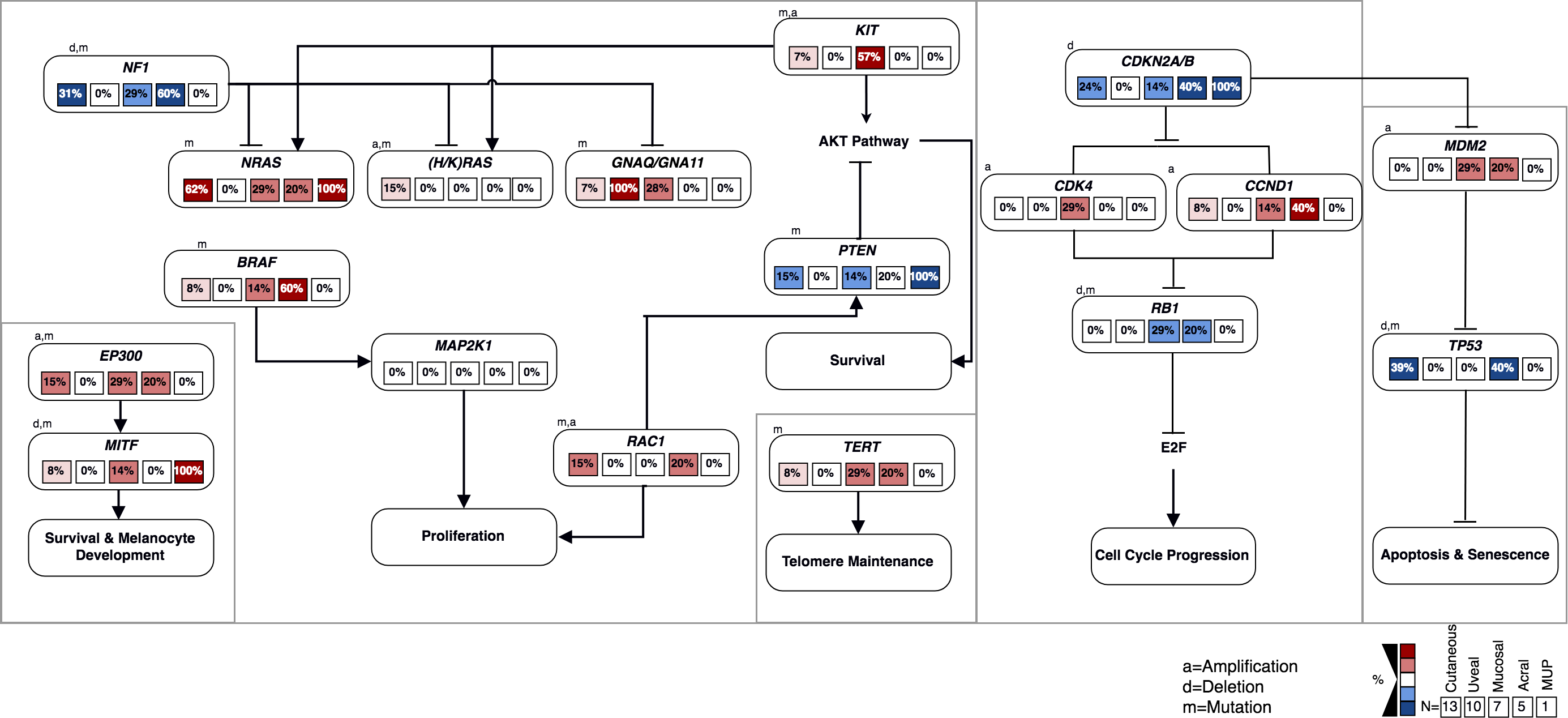

Supplement: S1 Fig — Recurrently impacted pathways in melanoma are summarized here in the context of somatic alterations observed across trial patients. The type of alteration is shown (amplification, deletion, mutation) and the percentage of patients of each subtype that demonstrate alterations in specific genes are shown and color-coded. The legend on the bottom right lists the total number of patients for each subtype. (PNG) [file pone.0248097.s012.png]

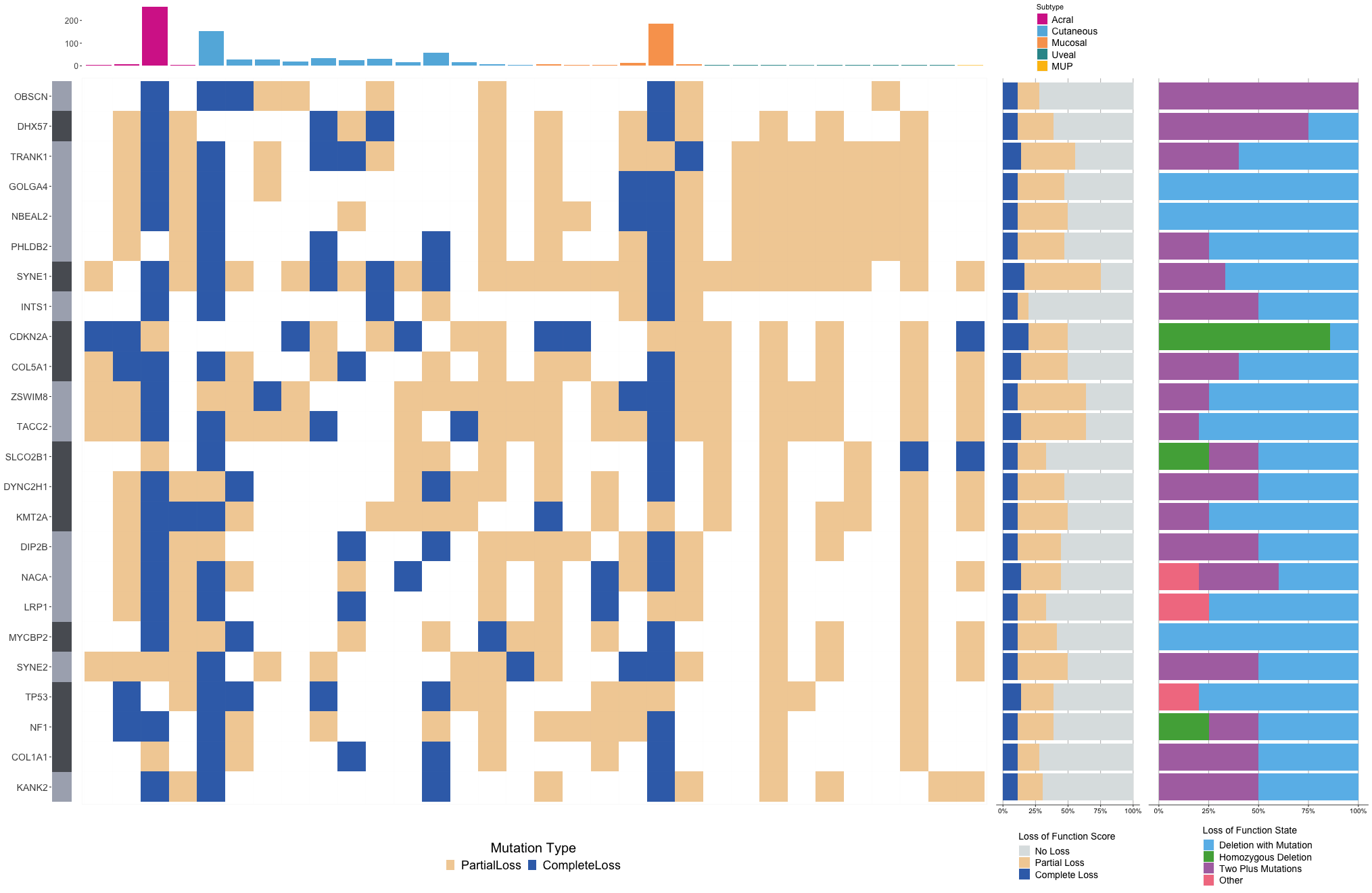

Supplement: S2 Fig — A: Loss of function (LOF) analysis—Predicted genes experiencing LOF are shown based on integration of DNA and RNA data. Each column represents an individual patient tumor. The central plot lists genes (Y-axis) demonstrating partial or complete LOF in each patient. The Loss of Function score plot indicates the proportion of all patients demonstrating a predicted partial or complete LOF. The right-most plot shows the breakdown of variants supporting a complete LOF only. B: Gain of function (GOF) analysis—Predicted genes experiencing GOF are shown based on integration of DNA and RNA data. Each column represents an individual patient tumor. The central plot lists genes (Y-axis) demonstrating GOF in each patient. The variant type plot indicates the proportion of all patients demonstrating a predicted GOF. Unlike the LOF predictions, a GOF prediction does not differentiate between partial and complete gain (see S1 File). (ZIP) [file pone.0248097.s013.zip › S2A_Fig.png]

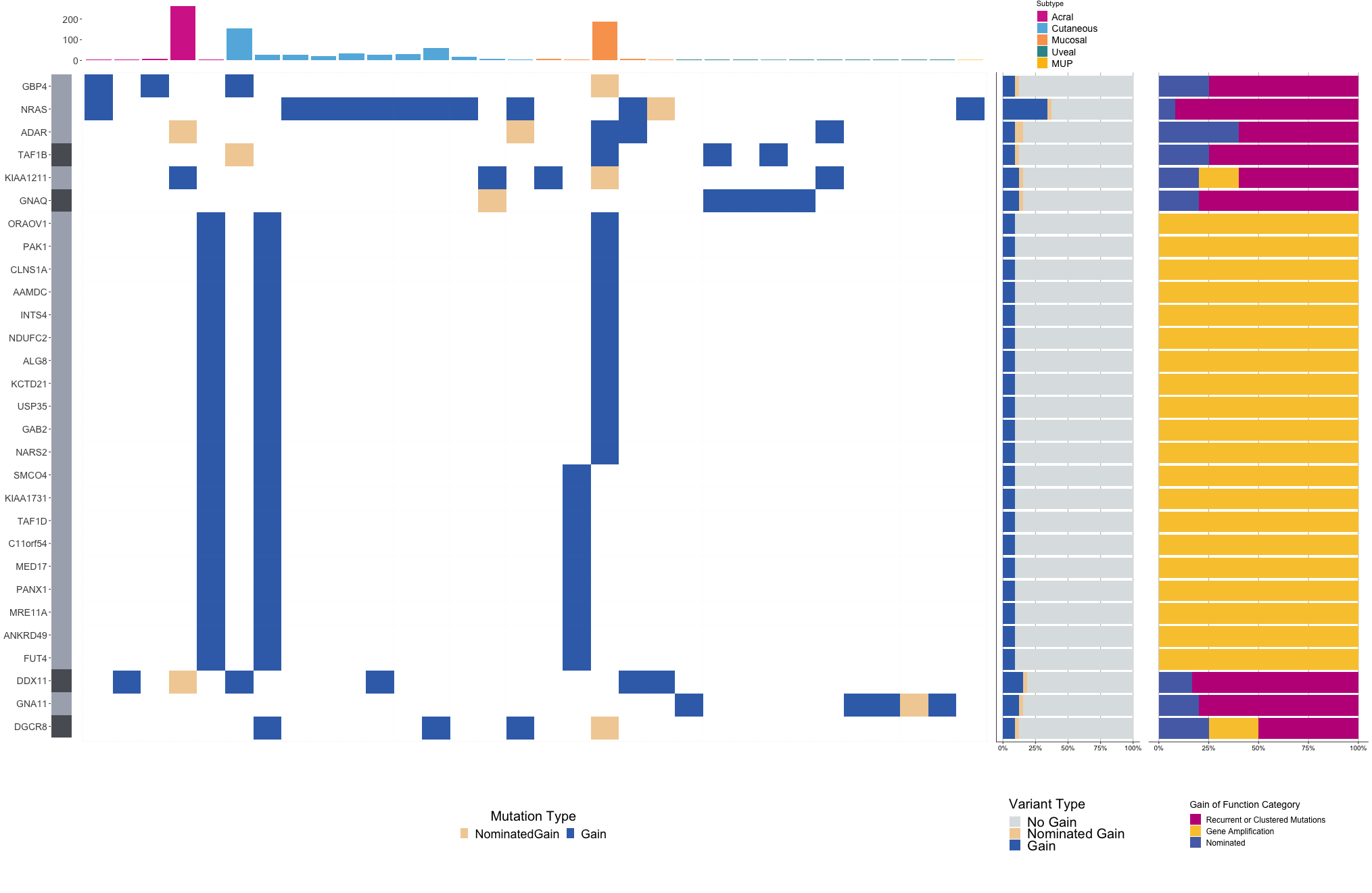

Supplement: S2 Fig — A: Loss of function (LOF) analysis—Predicted genes experiencing LOF are shown based on integration of DNA and RNA data. Each column represents an individual patient tumor. The central plot lists genes (Y-axis) demonstrating partial or complete LOF in each patient. The Loss of Function score plot indicates the proportion of all patients demonstrating a predicted partial or complete LOF. The right-most plot shows the breakdown of variants supporting a complete LOF only. B: Gain of function (GOF) analysis—Predicted genes experiencing GOF are shown based on integration of DNA and RNA data. Each column represents an individual patient tumor. The central plot lists genes (Y-axis) demonstrating GOF in each patient. The variant type plot indicates the proportion of all patients demonstrating a predicted GOF. Unlike the LOF predictions, a GOF prediction does not differentiate between partial and complete gain (see S1 File). (ZIP) [file pone.0248097.s013.zip › S2B_Fig.png]

# GAINS

G-score

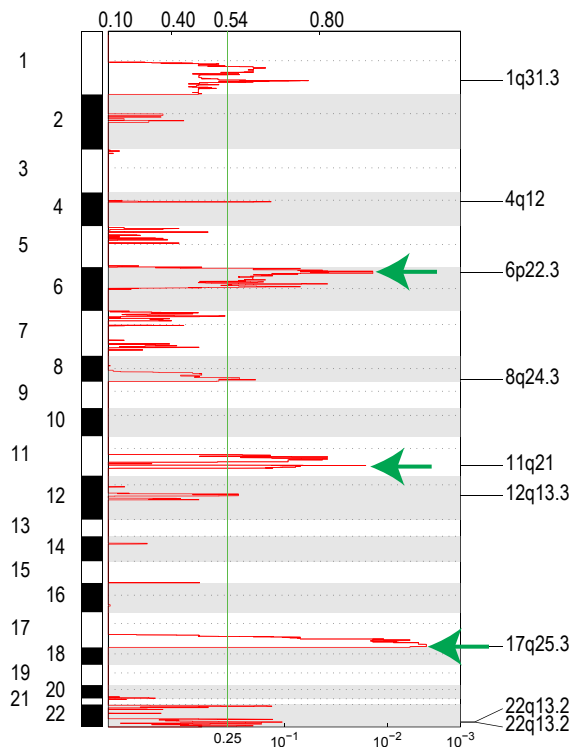

Significance (Q-value)

# LOSSES

G-score

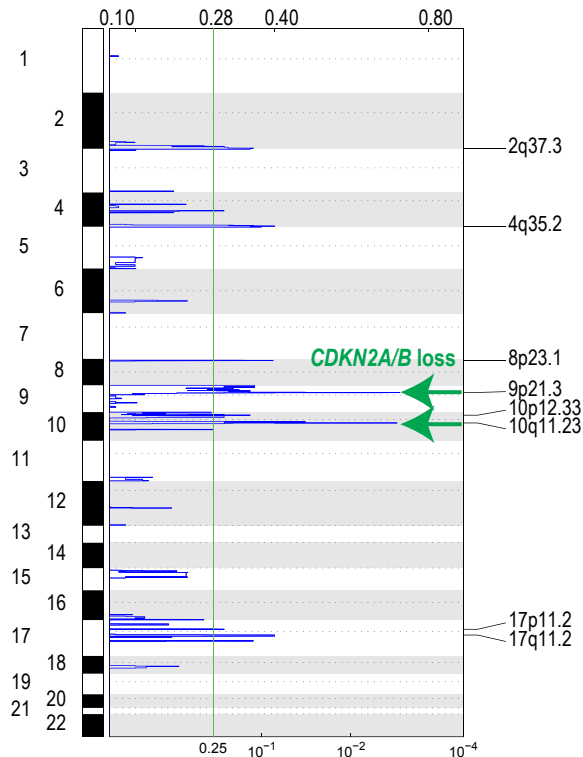

Significance (Q-value)

Supplement: S3 Fig — Consensus regions of gains (left, red) and losses (right, blue) across the entire cohort are shown. Green arrows mark significant regions (Q<0.05). Q-values are shown on the lower x-axis (Benjamini & Hochberg FDR), G-scores are shown on the upper x-axis, and chromosome numbers are labeled along the y-axis. (PDF) [file pone.0248097.s014.pdf]

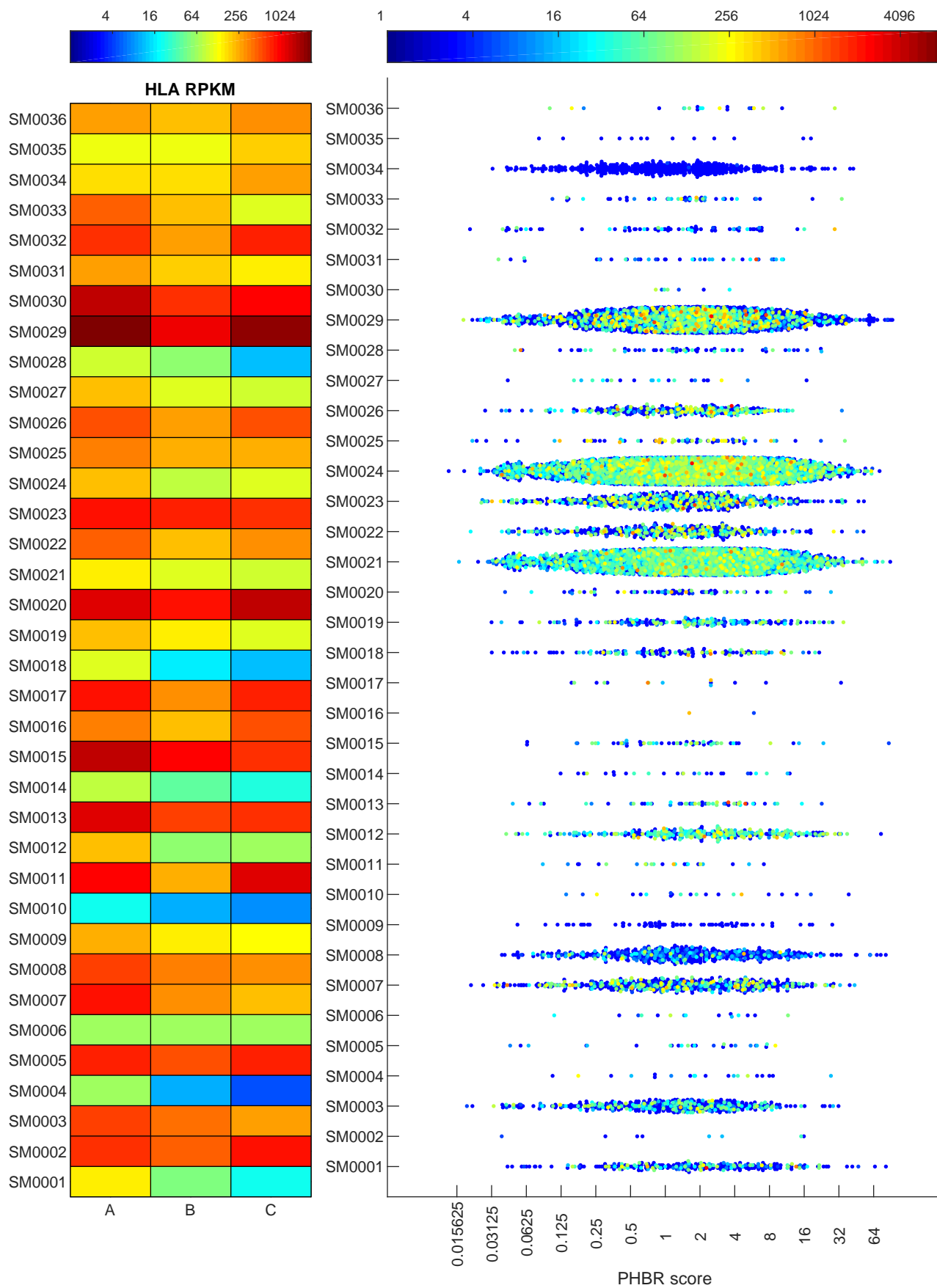

Supplement: S4 Fig — A: HLA-A, B, and C expression for each patient is shown on the left plot. Predicted neo-antigen expression, along with the predicted binding of HLA-A, B, or C to the neo-antigen, is shown on the right plot. B: Neo-antigen counts plotted against mutation burden (mutations per Mb) revealed a trend towards significance based on a Pearson’s correlation. (ZIP) [file pone.0248097.s015.zip › S4A_Fig.pdf]

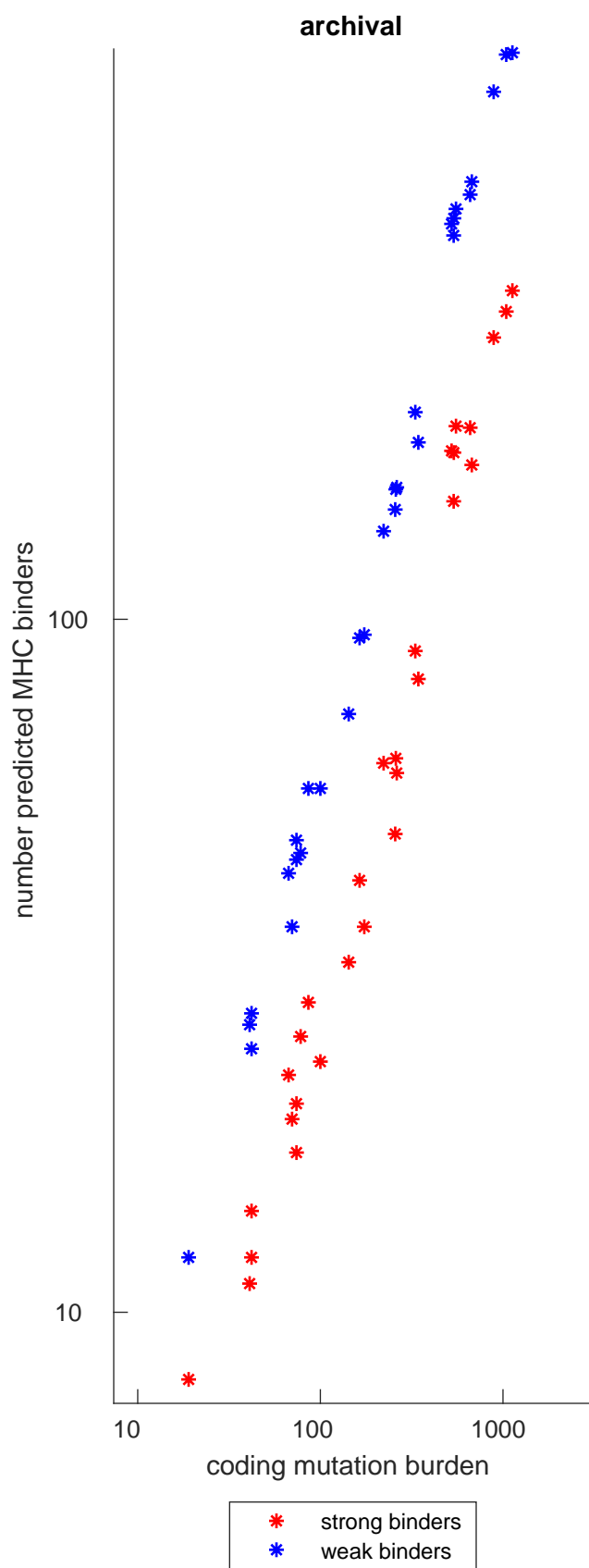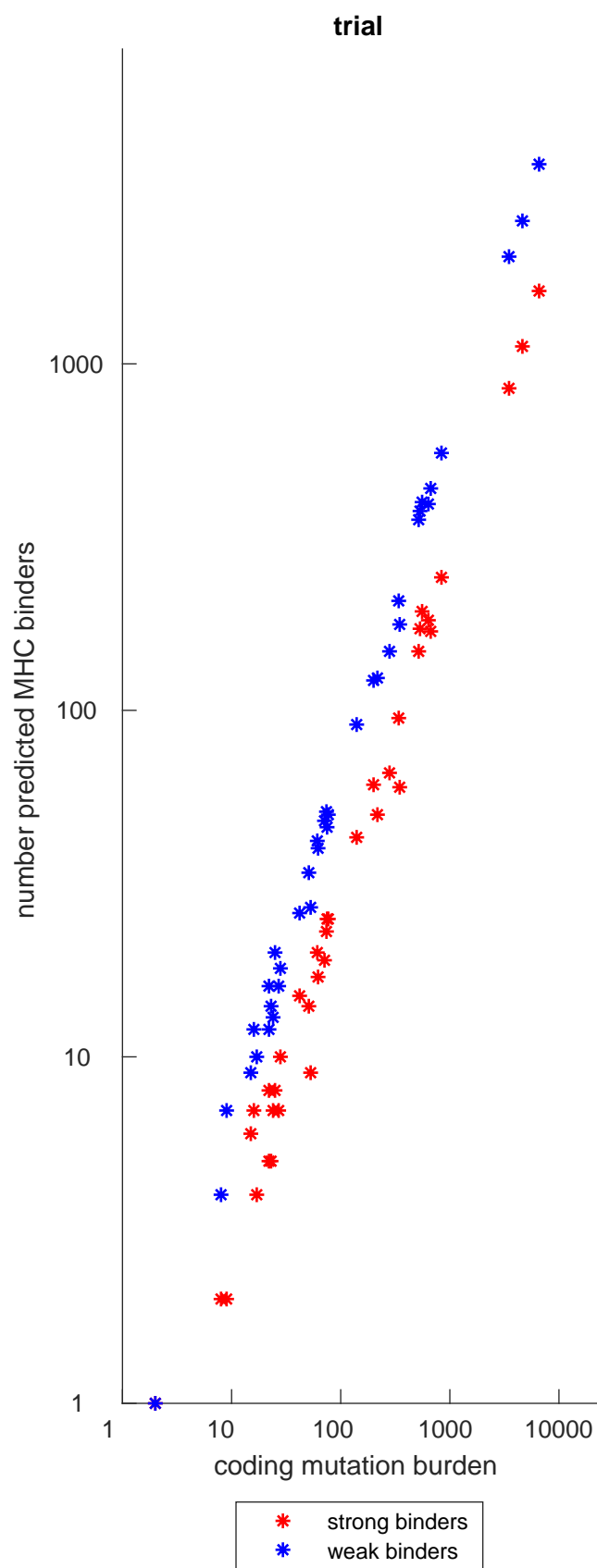

Supplement: S4 Fig — A: HLA-A, B, and C expression for each patient is shown on the left plot. Predicted neo-antigen expression, along with the predicted binding of HLA-A, B, or C to the neo-antigen, is shown on the right plot. B: Neo-antigen counts plotted against mutation burden (mutations per Mb) revealed a trend towards significance based on a Pearson’s correlation. (ZIP) [file pone.0248097.s015.zip › S4B_Fig.pdf]
