# Supplementary material for: Identifying treatment options for BRAFV600 wild-type metastatic melanoma: A SU2C/MRA genomics-enabled clinical trial
Source: PLoS One. 2021 Apr 7;16(4):e0248097. doi: 10.1371/journal.pone.0248097 (PMC8026051; doi:10.1371/journal.pone.0248097)
Supplement: S1 Protocol — (PDF) [file pone.0248097.s016.pdf]

Stand Up To Cancer Consortium
Genomics-Enabled Medicine for Melanoma (G.E.M.M.): Using Molecularly-Guided Therapy for Patients with BRAF wild-type (BRAFwt) Metastatic Melanoma

AMENDMENT #7: SUMMARY OF CHANGES

#	Section	Page (s)	Change
1.	All	All	Updated Version date to 10/21/2016 in Footer
2.	Face Page	4	Removed Indiana University from list of Participating Institutions
3.	Face Page	5	Added Daniel Zelterman and Michelle Deveaux as statisticians
4.	Face Page	5-6	Removed Lisa Fox and added Manuel Avedissian as Coordinating Site Project Manager and updated contact information
5.	Face Page	6	Updated version number and date
6.	2	13	Removed Indiana University from list of clinical sites
7.	7.1	25	Revised general guideline registration procedures section to remove Lisa Fox and add Manuel Avedissian as Coordinating Site Project Manager and updated contact information
8.	7.2	26	Revised registration process section to remove Lisa Fox and add Manuel Avedissian as Coordinating Site Project Manager and updated contact information
9.	Appendix XIII, 1.1.2	129	Added reference to package insert for andriamycin (doxorubicin)
10.	Appendix XIII, 1.1.3	133	Added reference to package insert for bortezomib
11.	Appendix XIII, 1.1.4	135	Revised Expected Adverse Events for carboplatin based on updated package insert
12.	Appendix XIII, 1.1.4	135-136	Added reference to package insert for carboplatin
13.	Appendix XIII, 1.1.5	138	Added reference to package insert for dacarbazine
14.	Appendix XIII, 1.1.6	143	Added reference to package insert for dasatinib
15.	Appendix XIII, 1.1.6	144-145	Revised dasatinib diaries to correct formatting issue
16.	Appendix XIII, 1.1.7	149	Added reference to package insert for erlotinib
17.	Appendix XIII, 1.1.7	149-151	Revised erlotinib diaries to correct formatting issue
18.	Appendix XIII, 1.1.8	152-153	Revised Expected Adverse Events for etoposide based on updated package insert
19.	Appendix XIII, 1.1.8	154	Added reference to package insert for etoposide
20.	Appendix XIII, 1.1.8	155	Revised etoposide diary to correct formatting issue
21.	Appendix XIII, 1.1.9	157	Revised Expected Adverse Events for gemcitabine based on updated package insert
22.	Appendix XIII, 1.1.9	158	Added reference to package insert for gemcitabine
23.	Appendix XIII, 1.1.10	164	Added reference to package insert for imatinib
24.	Appendix XIII, 1.1.10	165-166	Revised imatinib diaries to correct formatting issue
25.	Appendix XIII,	173	Added reference to package insert for interferon

	1.1.11		
26.	Appendix XIII, 1.1.11	174-175	Revised interferon diary to correct formatting issue
27.	Appendix XIII, 1.1.12	179	Added reference to package insert for paclitaxel
28.	Appendix XIII, 1.1.13	183	Added reference to package insert for pemetrexed
29.	Appendix XIII, 1.1.14	187	Added reference to package insert for sorafenib
30.	Appendix XIII, 1.1.14	188-189	Revised sorafenib diaries to correct formatting issue
31.	Appendix XIII, 1.1.15	193	Added reference to package insert for temozolomide
32.	Appendix XIII, 1.1.15	194	Revised temozolomide diary to correct formatting issue
33.	Appendix XIII, 1.1.16	197	Added reference to package insert for vorinostat
34.	Appendix XIII, 1.1.16	198-199	Revised vorinostat diaries to correct formatting issue
35.	Appendix XIII, 1.2.1	216	Revised alisertib (MLN8237) diary to correct formatting issue
36.	Appendix XIII, 1.2.2	237-238	Revised MLN9708 diaries to correct formatting issue
37.	Appendix XIII, 1.3.1.1	244	Revised Interaction with Other Medicinal Products and Other Forms of Interaction section for axitinib based on updated Investigator's Brochure
38.	Appendix XIII, 1.3.1.1	249-252	Revised Expected Adverse Events section for axitinib based on updated Investigator's Brochure and corrected typos in section
39.	Appendix XIII, 1.3.1.1	252	Updated pharmaceutical section for axitinib
40.	Appendix XIII, 1.3.1.1	257-258	Revised axitinib diaries to correct formatting issue
41.	Appendix XIII, 1.3.1.1	259	Revised Reference section for new axitinib Investigator's Brochure
42.	Appendix XIII, 1.3.1.2	262	Revised exclusion criteria for bosutunib to require Hepatitis B testing and exclude patients with a positive Hepatitis B surface antigen (HBsAg) test due to reports of Hepatitis B reactivation in patients receiving this class of drugs per updated Investigator's Brochure
43.	Appendix XIII, 1.3.1.2	267-269	Revised bosutinib expected adverse events based on updated Investigator's Brochure and corrected formatting
44.	Appendix XIII, 1.3.1.2	270	Minor changes to bosutinib Pharmaceutical section based on updated Investigator's Brochure
45.	Appendix XIII, 1.3.1.2	271	Added Hepatitis B panel at screening in the bosutinib calendar
46.	Appendix XIII, 1.3.1.2	274-275	Revised bosutinib diaries to correct formatting issue
47.	Appendix XIII, 1.3.1.2	276	Revised Reference section with updated bosutinib Investigator's Brochure version
48.	Appendix XIII, 1.3.1.3	296-297	Revised sunitinib diaries to correct formatting issue
49.	Appendix XIII, 1.3.1.5	329-330	Revised crizotinib diaries to correct formatting issue
50.	Appendix XIII, 1.3.1.6	332-349	Added commercial drug name IBRANCE throughout palbociclib (PD-0332991) appendix as Pfizer will be supplying the commercial product
51.	Appendix XIII, 1.3.1.6	332	Added reference to the IBRANCE package insert in the Background section for palbociclib
52.	Appendix XIII, 1.3.1.6	342-343	Added fetal harm risk based on IBRANCE (palbociclib) Investigator's Brochure and package insert and corrected typo in palbociclib Expected Advere Event section
53.	Appendix XIII, 1.3.1.6	344-345	Revised palbociclib (PD-0332991, IBRANCE) study calendar to include CBC w/diff on day 15 of the first 2 cycles based on IBRANCE package insert recommendations; corrected typo in footnote #12
54.	Appendix XIII, 1.3.1.6	346-348	Added additional drug names for PD-0332991 (palbociclib, IBRANCE) in patient diaries and reformatted diaries
55.	Appendix XIII,	349	Added IBRANCE (palbociclib) package insert information to Reference section

	1.3.1.6		
56.	Appendix XIII, 2.3.2.1	365-366	Revised dacomitinib diaries to correct formatting issue
57.	Appendix XIII, 1.4.1	377-378	Revised pexidartinib (PLX3397) expected adverse events based on safety letter and guidance from pharmaceutical company
58.	Appendix XIII, 1.4.1	382-383	Revised pexidartinib (PLX3397) diaries to correct formatting issue
59.	Appendix XIII, 1.5.1	406-408	Revised expected adverse events based on updated Investigator's Brochure for BGJ398
60.	Appendix XIII, 1.5.1	408-409	Minor revisions to the Pharmaceutical section based on updated BGJ398 Investigator's Brochure
61.	Appendix XIII, 1.5.1	414-415	Revised BGJ398 diaries to correct formatting issue
62.	Appendix XIII, 1.5.1	416	Revised Reference section with updated BGJ398 Investigator's Brochure version
63.	Appendix XIII, 1.6.1	435-437	Revised expected adverse events based on updated MEK162 Investigator's Brochures
64.	Appendix XIII, 1.6.1	438	Minor changes to the MEK162 pharmaceutical text based on updated Investigator's Brochure
65.	Appendix XIII, 1.6.1	439	Adjusted formatting before MEK162 study calendar to remove blank page
66.	Appendix XIII, 1.6.1	441	Revised Laboratory Assessment table for MEK162 to match Study Calendar
67.	Appendix XIII, 1.6.1	443-444	Revised MEK162 diaries to correct formatting issue
68.	Appendix XIII, 1.6.1	445	Revised Reference section with updated MEK162 Investigator's Brochure versions
69.	Appendix XIII, 1.7.1	455-456	Revised Potential Drug Interaction section for cabozantinib (XL184) based on updated Investigator's Brochure
70.	Appendix XIII, 1.7.1	459-470	Revised language in Warnings and Precautions and Guidelines for Management of Adverse Events section for cabozantinib (XL184)
71.	Appendix XIII, 1.7.1	477-480	Revised Expected Adverse Events section for cabozantinib (XL184) based on updated Investigator's Brochure and guidance from pharmaceutical company
72.	Appendix XIII, 1.7.1	481	Revised pharmaceutical section for cabozantinib (XL184) to reflect available strengths as per updated Investigator's Brochure and updated drug product section
73.	Appendix XIII, 1.7.1	488-489	Revised cabozantinib (XL184) patient diaries to allow water (drug to be taken in fasted state but water is allowed) per Investigator's Brochure and revised formatting
74.	Appendix XIII, 1.7.1	490	Revised Reference section to update Investigator's Brochure version information

Stand Up To Cancer Consortium Genomics-Enabled Medicine for Melanoma (G.E.M.M.): Using Molecularly-Guided Therapy for Patients with BRAF wild-type (BRAFwt) Metastatic Melanoma

**Coordinating Center:**	Yale University<br>New Haven, CT
**Protocol Principal Investigator:**	Patricia M. LoRusso, D.O.<br>Associate Director of Innovative Medicine<br>Yale Cancer Center<br>333 Cedar Street, WWW217<br>P.O. Box 208028<br>New Haven, CT 06520-8028<br>Phone: (203) 785-5944<br>Fax: (203) 785-4116<br>E-mail: patricia.lorusso@yale.edu
**Participating Institutions:**	Barbara Ann Karmanos Cancer Institute (KCI)<br>Detroit, MI
	The Translational Genomics Research Institute (TGen)<br>Phoenix, Arizona (nonclinical)
	University of Michigan Comprehensive Cancer Center (UMCCC)<br>Ann Arbor, MI
	Mayo Clinic<br>Rochester, MN; Scottsdale, AZ; Jacksonville, FL
	J. Craig Venter Institute<br>La Jolla, CA (nonclinical)
	Sanford-Burnham Medical Research Institute<br>La Jolla, CA (nonclinical)
	Charles A. Sammons Cancer Center/Baylor University Medical Center<br>Dallas, TX
	Vanderbilt University<br>Nashville, TN
	Columbia University<br>New York, NY
	Yale Cancer Center<br>New Haven, CT
	George Mason University<br>Manassas, VA (nonclinical)
	Smilow Cancer Hospital at Yale-New Haven Hospital<br>New Haven, CT

**Medical Overseers:**	Nicholas J Vogelzang MD<br>US Oncology Research<br>Comprehensive Cancer Centers of Nevada, Las Vegas, NV<br>3430 S Eastern Ave<br>Las Vegas, NV 89169<br>Phone (o): 702 952-3400<br>Phone (c): 702 343-4397<br>Fax: 702 952-3453<br>Nicholas.Vogelzang@USONCOLOGY.COM<br><br>Joseph Paul Eder, M.D.<br>Yale Cancer Center<br>333 Cedar Street<br>WWW 211, PO Box 208028<br>New Haven, CT 06520-8028<br>Ph: 203-737-1906<br>Fax: 203-785-4116<br>Email: joseph.eder@yale.edu<br><br>Samuel Ejadi, M.D.<br>Email: sejadi@oncologyscience.onmicrosoft.com
**Study Statisticians**	Daniel Zelterman, Ph.D.<br>Yale University Professor of Biostatistics<br>60 College St<br>New Haven, CT 06510-3210<br>Email: daniel.zelterman@yale.edu<br><br>Michelle DeVeaux, M.Phil.<br>Yale University Graduate School of Arts & Science<br>Email: michelle.deveaux@yale.edu<br><br>M. Anthony Schork, Ph.D.<br>Emeritus Professor of Biostatistics<br>School of Public Health<br>University of Michigan<br>Address: 217 Buena Vista Ave, Ann Arbor, MI 48103<br>Phone: 734-660-9002<br>Email: tandlschork@gmail.com<br>(Note: Dr. Schork is not participating in patient care for this study)
**Coordinating Site Project Manager:**	Manuel Avedissian, M.D.<br>Project Manager, SU2C<br>2 Church Street South, Suite 414<br>New Haven, CT 06519<br>Phone: 203-737-3669

Fax: 203- 785-3500
Email : manuel.avedissian@yale.edu

Coordinating Site Pharmacist
Thomas M. Ferencz R.Ph., BCOP
Smilow Cancer Hospital at Yale-New Haven
Oncology Pharmacy, 8th floor room 8-110
266 South Frontage Rd, New Haven, CT 06510
Phone: 203-200-4455
Fax: 203-200-4445
Email: Thomas.Ferencz@ynhh.org

Lead Clinical Project Manager :
Mary Jo Pilat Ph.D, MS, PA-C, CCRP
Assistant Professor-Clinical
259 Mack Avenue
Suite 2590
Detroit, MI 48201
Office: (313) 577-4635
Fax: (313) 577-5467
Email: mjpilat@wayne.edu

Yale Protocol#: HIC#1408014446

IND/IDE #: IND #115393

Protocol Version:
11/22/2013 (original)
01/13/2014 Amendment #1
04/10/2014 Amendment #2
08/04/2014 Amendment #3
11/11/2014 Amendment #4
04/30/2015 Amendment #5
03/15/2016 Amendment #6
10/21/2016 Amendment #7

TABLE OF CONTENTS

1 LIST OF ABBREVIATIONS 10
2 PROTOCOL SUMMARY 12
3 STUDY SCHEMA 14
4 OBJECTIVES 15
4.1 PRIMARY OBJECTIVE 15
4.2 SECONDARY OBJECTIVES 15
4.3 EXPLORATORY OBJECTIVES 15
5 BACKGROUND 15
6 ELIGIBILITY TO PARTICIPATE 21
6.1 INCLUSION CRITERIA 21
6.2 EXCLUSION CRITERIA 23
6.3 INCLUSION OF WOMEN AND MINORITIES 25
7 REGISTRATION PROCEDURES 25
7.1 GENERAL GUIDELINES 25
7.2 REGISTRATION PROCESS 25
8 STUDY DESIGN 26
8.1 STUDY WORKFLOW 27
8.2 GENERAL CONCOMITANT MEDICATION AND SUPPORTIVE CARE GUIDELINES 39
8.3 DURATION OF STUDY 40
8.4 DURATION OF FOLLOW-UP 41
8.5 CRITERIA FOR REMOVAL FROM STUDY: 41
8.6 SAFETY EVALUATIONS 41
9 ADVERSE EVENTS: LIST AND REPORTING REQUIREMENTS 42
9.1 DEFINITIONS 42
9.2 GRADING AND RELATEDNESS OF ADVERSE EVENTS 44
9.3 DOCUMENTING ADVERSE EVENTS 45
9.4 REPORTING OF ADVERSE EVENTS 45
10 SCHEDULE OF ASSESSMENTS AND PROCEDURES 47
10.1 PRE-STUDY ASSESSMENTS AND PROCEDURES 47
10.2 FOLLOWING VERIFICATION OF ELIGIBILITY 48
10.3 FOLLOWING MOLECULAR PROFILING AND TUMOR BOARD/MEDICAL OVERSEER EVALUATION: 48
10.4 ADDITIONAL TREATMENT CYCLES (REGULAR MAINTENANCE MONITORING): 49
10.5 OFF TREATMENT VISIT: 50
10.6 30 DAY FOLLOW UP (END OF STUDY) VISIT 50
10.7 STUDY CALENDAR (PRE-TREATMENT) 52
11 PROTOCOL DRUGS 54
11.1 DRUG ACCOUNTABILITY 54
11.2 MOLECULARLY GUIDED THERAPY 54
12 LABORATORY EVALUATIONS 55
12.1 DNA AND RNA ANALYSIS (ASHION ANALYTICS A CENTRAL CLIA-CERTIFIED LABORATORY AFFILIATED WITH TGEN) 55
12.2 ANALYSIS OF CIRCULATING TUMOR NUCLEIC ACIDS (CTNA) AT TGEN 55
12.3 ESTABLISHMENT OF PRIMARY BRAFWT MM TUMORGRAFT MODELS 56

12.4 REVERSE PHASE PROTEIN MICROARRAY 57
12.5 T CELL 58
12.6 QUALITY OF LIFE. 58
12.7 MERCK SHARP & DOHME (RNA GENE EXPRESSION PROFILING) 59

13 MEASUREMENT OF EFFECT 59

13.1 ANTITUMOR EFFECT – SOLID TUMORS 59

14 STATISTICS 65

14.1 PRIMARY ANALYSIS 65
14.2 SECONDARY ANALYSES 66
14.3 ACCRUAL RATE AND TRIAL DURATION 66
14.4 FOLLOW-UP TIMES 66

15 TISSUE DONOR PRIVACY AND CONFIDENTIALITY 67

15.1 ACCESS TO BIOSPECIMENS AND DONOR INFORMATION 68
15.2 RESEARCH WITH DE-IDENTIFIED SPECIMENS 68
15.3 BIOSPECIMEN BANKING 68
15.4 FUTURE USE OF BANKED SAMPLES: 69
15.5 RESTRICTIONS ON SAMPLE USAGE 69
15.6 INCIDENTAL FINDINGS. 69

16 STUDY DATA HANDLING AND RECORD KEEPING 69

16.1 DEPOSITION OF GENOTYPE RESULTS TO PUBLIC DATABASES 70

17 PLAN FOR COMMUNICATION OF COLLABORATING SITES 71

18 ETHICAL CONSIDERATIONS 71

19 PRE-STUDY DOCUMENTATION 72

20 NEW PROTOCOL DISTRIBUTION 73

21 SITE INITIATION AND MONITORING 74

22 IRB REQUIREMENTS 74

23 DATA AND SAFETY MONITORING 75

24 REFERENCES: 77

25 LIST OF APPENDICES 80

APPENDIX I: PERFORMANCE STATUS CRITERIA 81

APPENDIX II: STUDY PHARMACOPEIA 82

APPENDIX III: BLOOD COLLECTION AND PROCESSING FOR EXTRACTION OF CONSTITUTIONAL ANALYTES AT ASHION ANALYTIS A CENTRAL CLIA-CERTIFIED LABORATORY AFFILIATED WITH TGEN 83

APPENDIX IV: FRESH FROZEN TUMOR COLLECTION AND SHIPMENT 86

APPENDIX V: COLLECTION/HANDLING/SHIPPING OF FRESH TISSUE FOR TUMORGRAFT IMPLANTING 90

APPENDIX VI: SHIPPING OF PARAFFIN BLOCKS AND SLIDES 93

APPENDIX VII: COLLECTION AND SHIPPING OF PLASMA FOR CIRCULATING TUMOR NUCLEIC ACIDS (CTNA) 94

APPENDIX VIII: CRYOPRESERVATION OF TISSUE IN OCT (PROTEOMICS) 97
APPENDIX IX: MERCK SHARP & DOHME 99
APPENDIX X: SAMPLE DISTRIBUTION FROM CLINICAL SITES 107
APPENDIX XI: ACKNOWLEDGMENT OF THE INVESTIGATORS 108
APPENDIX XII: DESCRIPTION OF TUMOR BOARDS 109
APPENDIX XIII: DRUG SPECIFIC APPENDICES ....ERROR! BOOKMARK NOT DEFINED.
1.1 COMMERCIALLY AVAILABLE AGENTS ....ERROR! BOOKMARK NOT DEFINED.
1.1.2 ADRIAMYCIN (DOXORUBICIN) SPECIFIC INFORMATION............ERROR! BOOKMARK NOT DEFINED.
1.1.3 BORTEZOMIB-SPECIFIC INFORMATION ....ERROR! BOOKMARK NOT DEFINED.
1.1.4 CARBOPLATIN-SPECIFIC INFORMATION............ERROR! BOOKMARK NOT DEFINED.
1.1.5: DARCABAZINE-SPECIFIC INFORMATION ....ERROR! BOOKMARK NOT DEFINED.
1.1.6: DASATINIB-SPECIFIC INFORMATION............ERROR! BOOKMARK NOT DEFINED.
1.1.7 ERLOTINIB-SPECIFIC INFORMATION............ERROR! BOOKMARK NOT DEFINED.
1.1.8 ETOPOSIDE-SPECIFIC INFORMATION ....ERROR! BOOKMARK NOT DEFINED.
1.1.9 GEMCITABINE-SPECIFIC INFORMATION ....ERROR! BOOKMARK NOT DEFINED.
1.1.10 IMATINIB-SPECIFIC INFORMATION............ERROR! BOOKMARK NOT DEFINED.
1.1.11 INTERFERON ALPHA 2 BETA-SPECIFIC INFORMATION............ERROR! BOOKMARK NOT DEFINED.
1.1.12 PACLITAXEL-SPECIFIC INFORMATION............ERROR! BOOKMARK NOT DEFINED.
1.1.13 PEMETREXED-SPECIFIC INFORMATION ....ERROR! BOOKMARK NOT DEFINED.
1.1.14 SORAFENIB- SPECIFIC INFORMATION............ERROR! BOOKMARK NOT DEFINED.
1.1.15 TEMOZOLOMIDE- SPECIFIC INFORMATION............ERROR! BOOKMARK NOT DEFINED.
1.1.16 VORINOSTAT- SPECIFIC INFORMATION ....ERROR! BOOKMARK NOT DEFINED.
1.2 MILLENNIUM AGENTS ....ERROR! BOOKMARK NOT DEFINED.
1.2.1 MLN8237 ....ERROR! BOOKMARK NOT DEFINED.
1.2.2 MLN9708 ....ERROR! BOOKMARK NOT DEFINED.
1.3 PFIZER AGENTS............ERROR! BOOKMARK NOT DEFINED.
1.3.1 COMMERCIALLY AVAILABLE PFIZER AGENTS............ERROR! BOOKMARK NOT DEFINED.
1.3.1.1 INLYTA (AXITINIB)............ERROR! BOOKMARK NOT DEFINED.
1.3.1.2 BOSULIF (BOSUTINIB)............ERROR! BOOKMARK NOT DEFINED.
1.3.1.3 SUTENT (SUNITINIB)............ERROR! BOOKMARK NOT DEFINED.
1.3.1.4 TORISEL (TEMSIROLIMUS)............ERROR! BOOKMARK NOT DEFINED.
1.3.1.5 XALKORI (CRIZOTINIB)............ERROR! BOOKMARK NOT DEFINED.
1.3.1.6 PALBOCICLIB (PD-0332991, IBRANCE®)............ERROR! BOOKMARK NOT DEFINED.
2.3.2 INVESTIGATIONAL PFIZER AGENTS ....ERROR! BOOKMARK NOT DEFINED.
2.3.2.1 DACOMITINIB (PF-00299804)............ERROR! BOOKMARK NOT DEFINED.
1.4 PLEXXIKON AGENTS............ERROR! BOOKMARK NOT DEFINED.
1.4.1 PEXIDARTINIB (PLX3397) ....ERROR! BOOKMARK NOT DEFINED.
1.5 NOVARTIS AGENTS ....ERROR! BOOKMARK NOT DEFINED.
1.5.1 BGJ398............ERROR! BOOKMARK NOT DEFINED.
1.6 ARRAY BIOPHARMA AGENTS............ERROR! BOOKMARK NOT DEFINED.
1.6.1 MEK162 (BINIMETINIB) ....ERROR! BOOKMARK NOT DEFINED.
1.7 EXELIXIS AGENTS............ERROR! BOOKMARK NOT DEFINED.
1.7.1 XL184 (CABOZANTINIB)............ERROR! BOOKMARK NOT DEFINED.

1 LIST OF ABBREVIATIONS

Abbreviation	Full text
AALAC	Association for Assessment and Accreditation of Laboratory Animal Care International
ACP	AmpliSeq Cancer Panel
ALT	alanine transaminase
BRAFwt	BRAF wild-type
C	Celsius
CBC	complete blood count
CCCN	Comprehensive Cancer Centers of Nevada
CLIA	Clinical Laboratory Improvement Amendments
CR	complete response
CRL	Clinical Reference Laboratories
CRF	case report form
CSPM	Coordinating Site Project Manager
CT	computed tomography
CTCAE	Common Terminology Criteria for Adverse Events
ctNA	circulating tumor nucleic acids
D	day
dbGaP	database of Genotypes and Phenotypes
DLT	dose limiting toxicity
DNA	deoxyribonucleic acid
DTIC	dacarbazine
ECOG	Eastern Cooperative Oncology Group
FFPE	formalin-fixed paraffin-embedded
FDA	Food and Drug Administration
GEMM	Genomics Enabled Medicine for Melanoma
GWAS	genome-wide association studies
H & E	hematoxylin and eosin
HHS	Health and Human Services
HIV	Human Immunodeficiency Virus
HIPAA	Health Insurance Portability and Accountability Act
HPLC	high performance liquid chromatography
HRPP	Human Subjects Research Protections Program
HWCRC	Hudson-Webber Cancer Research Center
IACUC	Institutional Animal Care and Use Committee
IDE	Investigational Device Exemption
IHC	immunohistochemistry
IND	Investigational New Drug
IRB	Institutional Review Board
KCI	Karmanos Cancer Institute
Kg	kilogram
LIMS	Laboratory Information Management System
mcg	microgram
mcL	microliter
MM	metastatic melanoma
Mg	milligram
mL	milliliter

MRA	Melanoma Research Alliance
MS	mass spectrometry
MTB	Molecular tumor board
NCI	National Cancer Institute
NGS	Next Generation Sequencing
NIH	National Institutes of Health
BORR	Best overall response rate
PGSEA	Parametric Gene Set Enrichment Analysis
PCR	polymerase chain reaction
PHI	protected health information
PrD	progressive disease
PET	positron emission tomography
PFS	progression free survival
PI	principal investigator
PR	partial response
PRMC	Protocol Review and Monitoring Committee
PLT	Platelet
PPE	personal protective equipment
PTT	partial thromboplastin time
QC	quality control
RECIST	Response Evaluation Criteria In Solid Tumors
RNA	ribonucleic acid
RNAt	total ribonucleic acid
RR	response rate
SAE	serious adverse event
SCID	severe combined immunodeficient
SD	stable disease
SGOT	serum glutamic-oxaloacetic transaminase
SGPT	serum glutamate pyruvate transaminase
SoC	standard of care
SOP	standard operating procedure
SU2C	Stand Up To Cancer
TBD	to be determined
TCGA	The Cancer Genome Atlas
TGen	The Translational Genomics Research Institute
TGT	Target intensity
TTP	Time to tumor progression
ULN	upper limit of normal
ULRR	upper institutional limit reference range
UMCCC	University of Michigan Comprehensive Cancer Center
VARI	Van Andel Research Institute
WGS	whole-genome sequencing
Wk	Week
WSU	Wayne State University
Wt	wild-type
XB-BIS	XenoBase-BioIntegration Suite
YCC	Yale Cancer Center
YCCI	Yale Center for Clinical Investigation

2 PROTOCOL SUMMARY

**Title of Study:**	Stand Up To Cancer Consortium Genomics Enabled Medicine for Melanoma (G.E.M.M.): Using Molecularly-Guided Therapy for Patients with BRAF wild-type (BRAFwt) Metastatic Melanoma
**Study PI**	Patricia M. LoRusso, D.O.
**Coordinating Site**	Yale University, New Haven CT USA
**Study Objectives**	Primary:<br>• To determine the difference in Best Overall Response Rate (BORR) between patients treated with MEK162 following personalized molecularly guided assignment vs. a historical BORR of 7% in this patient population.<br><br>Secondary<br>• To evaluate the safety of performing individualized drug therapy (including novel agents and commercially-available agents) in the context of a personalized medicine clinical trial<br>• To define the difference in progression free survival (PFS) between patients treated with MEK162 following personalized molecularly guided assignment vs. a historical PFS rate of 2 months in this patient population<br>• To continually assess data obtained in real time so as to iteratively refine and standardize a set of statistical and informatics methodologies for matching treatments to the patient's tumor, based on their molecular profile.
**Study Design**	This is a Phase 2, prospective, multi-center, open-label study with a Simon 2-Stage optimal design.<br><br>Patients with BRAFwt metastatic melanoma who are refractory or relapsed on (or decide against) conventional therapy will undergo a biopsy procedure. Specimens will be sent to TGen for next-generation sequencing and gene expression profiling. A list of mutations, inserts and deletions, and copy number variations will be identified by deep molecular sequencing for each patient on a case by case basis, depending on the mutations found in each patient's cancer. Based on the molecular signature, a suggested treatment plan will be formulated by a molecular and clinical tumor board and reviewed by an independent medical overseer. Based on the initial 23 patients enrolled on this study who underwent molecular profiling and tumor board evaluation, 20 were assigned MEK162. Patients who are assigned and receive MEK162 will be evaluable in the primary endpoint of BORR compared to a historical response rate of 7% in this patient population. Patients not assigned MEK162 will receive their assigned treatment, but will not be evaluable for the primary endpoint. Extent of disease will be initially measured at 30-35 days, and at 8 or 9 week intervals (depending on length of cycle) following initial assessment.<br><br>The Simon two-stage optimal design will enroll 22 MEK162 patients and terminate early if one or fewer patients respond (1/22 = 4.5%) If there are 2 or more MEK162 patients responding then we will enroll an additional 25 for a total of 47 receiving MEK162. If 6 or more of these 47 patients respond then we will reject the lower response rate of 7% in favor of a higher rate.

**No. of Patients / Clinical Sites**	Up to 47 evaluable BRAFwt metastatic melanoma patients with adequate tissue for molecular analysis (patients found to be ineligible or with inadequate tissue for molecular analysis will be replaced) who are assigned MEK162 following molecular analysis.<br><br>Patients will enroll at the Karmanos Cancer Institute (KCI), Mayo Clinic (Scottsdale, Rochester, and Jacksonville), University of Michigan Comprehensive Cancer Center (UMCCC), Charles A. Sammons Cancer Center/Baylor University Medical Center, Vanderbilt University, Columbia University and Yale Cancer Center
**Inclusion/ Exclusion Criteria:**	See Section 6 for detailed eligibility criteria.
**Medication and Doses:**	Treatment medication will vary for individual patients based on their particular molecular signature. Patients will be administered the assigned treatment according to the specific dose and schedule described in the Investigator's Brochure (for investigational agents) or package insert (for commercial agents). Drug combination therapeutic treatments will be allowed, providing the specific drugs are included in the study pharmacopeia, the combination has already been tested in at least one Phase 1 clinical trial, and a recommended Phase 2 dose and schedule has been determined. Only patients assigned MEK162 will be evaluable for the primary endpoint of BORR vs. historical control.
**Estimated Duration:**	Accrual to this study is estimated to be approximately 18 months, with follow-up for up to one year after last patient enrollment.
**Study Procedures:**	Refer to Study Calendar for timing of study procedures.
**Criteria For Evaluation:**	Primary:<br>• Overall response rate will be assessed by the presence of radiologically and/or clinically assessable disease before treatment and after at least 30 days of treatment.<br><br>Secondary<br>• Safety analysis will be conducted on all patients who have received at least one dose of therapy, and will include the frequency of all adverse events and laboratory abnormalities as well as frequency of dose interruptions, dose reductions, and treatment discontinuation<br>• Progression Free Survival (PFS) is defined as the duration of time from start of treatment to time of progression or death, whichever occurs first.

3 Study Schema

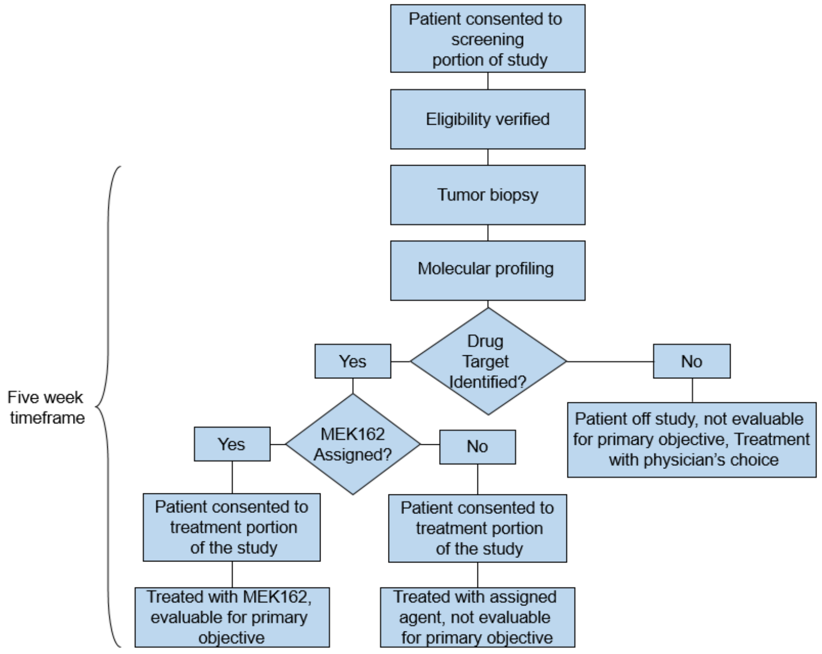

This is a Phase 2, prospective, multi-center, open-label study with a Simon 2-Stage optimal design. Patients with metastatic BRAFwt MM whose tumors have progressed following previous treatment with standard therapy (e.g.immunotherapy) or for which immunotherapy was not given due to co-morbidities, patient refusal, etc. will undergo initial evaluation for eligibility. Following consent, they will undergo screening tests as well as a tumor biopsy and blood draw. Specimens from all sites will be managed and sent to Ashion Analytics, LLC (Ashion) a central CLIA-certified laboratory affiliated with TGen, for study pathology evaluation and molecular analyte generation. The tumor sample will be retained under a "chain of custody" within Ashion. Separate tumor samples from a subset of patients will be sent to the Mayo Clinic for the establishment of primary patient tumorgrafts in immune compromised mice.

For each qualified tumor (DNA/RNA) and blood (DNA) sample, extracted analytes will be sent from Ashion to an adjacent laboratory at TGen for Next Generation Sequencing (NGS). Data obtained will be processed by the Genome Processing and Knowledge Recovery Teams, and a list of mutations will be generated. The molecular data will be presented to a Molecular Tumor Board who will assess the molecular profile generated for the individual patient and analyze the weight of evidence in support of a proposed target/drug match. This information will then be discussed by a Clinical Tumor Board who will determine an individualized treatment plan based on the available data. If a druggable target/pathway is found, the results will be verified by Ashion as needed. The formulated treatment plan will be reviewed by an independent medical overseer and additional screening will occur to ensure the patient is eligible for the selected treatment. Based upon our group's participation in similar trials, we anticipate the time from patient biopsy to initiation of treatment to be approximately 5 weeks. Attempts will be made to re-biopsy patients should they come off study (for disease progression, unacceptable toxicity, etc.), and their mutational status compared with the original genomic analysis.

Patients who are assigned and receive MEK162 will be evaluable in the primary endpoint of Best Overall Response Rate (BORR) compared to a historical response rate of 7% in this

patient population. Patients not assigned MEK162 will receive their assigned treatment, but will not be evaluable for the primary endpoint. Extent of disease will be initially measured at 30-35 days, and at 8 or 9 week intervals (depending on length of cycle) following initial assessment.

The Simon two-stage optimal design will enroll 22 MEK162 patients and terminate early if one or fewer patients respond (1/22 = 4.5%) If there are 2 or more MEK162 patients responding then we will enroll an additional 25 for a total of 47 receiving MEK162. If 6 or more of these 47 patients respond then we will reject the lower response rate of 7% in favor of a higher rate.

4 Objectives

4.1 *Primary Objective*

To determine the difference in Best Overall Response Rate (BORR) between patients treated with MEK162 following personalized molecularly guided assignment vs a historical BORR of 7% in this patient population.

4.2 *Secondary Objectives*

4.2.1 To evaluate the safety of performing individualized drug therapy (including novel agents and commercially-available agents) in the context of a personalized medicine clinical trial

4.2.2 To define the difference in progression free survival (PFS) between patients treated with MEK162 following personalized molecularly guided assignment vs. a historical PFS rate of 2 months in this patient population

4.2.3 To continually assess data in real time so as to iteratively refine and standardize a set of statistical and informatics methodologies for matching treatments to the patient's tumor, based on their molecular profile.

4.3 Exploratory Objectives

4.3.1 Quality of Life (QoL) Alteration. See section 12.6 for description.

5 Background

Up until recently, patients with advanced metastatic melanoma (MM) have had a poor prognosis, with a median survival of only 6–9 months and a 3-year survival rate of only 10–15% (2). For decades, no single drug or drug combination has demonstrated any appreciable impact on survival (3). Standard-of-care chemotherapy such as dacarbazine or temozolomide has a low overall response (OR) rate in MM patients, ranging from 5-10% and a low progression-free survival (PFS) rate of approximately 2 months (4-8). Nevertheless, the past few years have shown encouraging advances in the treatment of MM. Notably, recently developed BRAF inhibitors have demonstrated clinical efficacy in patients harboring oncogenic BRAF mutations (9, 10). They have extended the median survival to 13-16 months (HR=0.70). These inhibitors represent a major shift in the way we think about and treat melanoma. However, as further improvement of this promising therapy continues, little progress has been made in identifying therapeutic targets to treat patients with wild-type BRAF (BRAFwt) tumors, which comprise ~50% of all MMs (11). While we have an available immunologic agent that extends patients survival, the checkpoint inhibitor, ipilimumab (anti-CTLA4), its response rate is 10% or below and can be associated with life threatening toxicity.

Importantly, a higher frequency of BRAFwt melanomas among the elderly (12), combined with the aging population trend, predicts a future shift in the prevalence of BRAFwt melanoma patients and highlights the importance of identifying better therapeutic approaches for them.

Many common cancers are difficult to treat, in part, because they are heterogeneous, with each tumor subset having different molecular abnormalities (13). Identifying relevant molecular aberrations within heterogeneous cancers is crucial to future progress in targeted therapeutics (13-15). Such mutations occur in genes that encode signaling proteins critical for cellular proliferation and survival. Melanoma has been particularly difficult to subdivide histopathologically; however, by molecular dissection, we are beginning to recognize molecular subtypes defined by specific 'driver' mutations with an increased likelihood of responding to the targeted therapy (Figure 1) (1, 16). Thus, while treatments like vemurafenib are indeed promising for MM patients with an oncogenic BRAF mutation, targeted therapies are clearly needed for patients presenting with BRAFwt MM. Without knowing the genomic make-up of BRAFwt MM, it will be extremely difficult to define appropriate and meaningful drug therapies for which to treat this disease. Very recently, groups including our own (17, 18), have begun to publish data from exome and limited whole-genome sequencing of melanomas. However, none of these studies were focused specifically on BRAFwt, and while data from these sources will help broadly define alterations in all subtypes of MMs, none of these data will provide meaningful information allowing the correlation between specific molecular aberrations and treatment outcome.

Personalized Medicine – An Overview

A challenge in the area of targeted cancer treatment is identifying optimal therapies to treat tumors that are both highly adaptive and exhibit significant inter- and intra-patient variation (19-21). "Personalized medicine" refers to the tailoring of medical treatment to the individual characteristics of each patient. Recent advances in research, including the sequencing of the human genome, are generating unprecedented opportunities for progress toward the vision of personalized medicine — tailoring risk reduction, diagnosis, and treatment strategies to individual patients to improve health, prevent disease, and potentially reduce healthcare costs (22, 23). A necessary component of such efforts is the development of key translational research tools (24, 25). Human specimens that serve as analytes for the new and developing technology platforms have emerged as a critical resource for basic and translational research in cancer, as they are a direct source of molecular data from which targets for therapy, detection, and prevention are identified and molecular toxonomies of cancer are derived (26).

In 2009, Stewart and Kurzrock published an article that challenged the current paradigm of drug development (15). Instead of spending millions of dollars on large randomized trials to identify miniscule survival gains in large populations, they argued, limited patient and financial resources would be better utilized defining molecular predictors that could identify a small number of patients with a relatively high probability of benefit that would permit patient pre-selection for a drug (15). If we properly select patients for sensitive mutations, a high proportion may benefit. Using theoretical modeling, they showed that restricting trials to tumors expressing a drug target could substantially reduce patient numbers and financial resources required to demonstrate efficacy (27). Further, this approach is likely to lead to more effective and durable therapeutic interventions over an unselective standard approach, thus improving patient survival and potentially sparing them from unnecessary toxicity, expense, and time wasted on ineffective therapies.

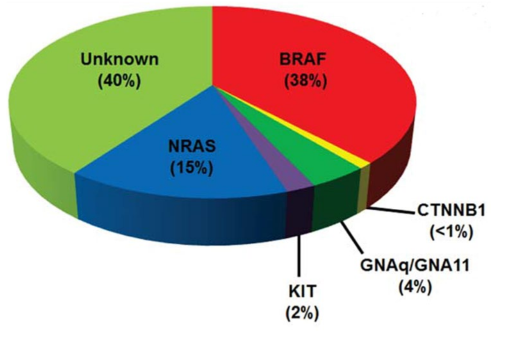

Figure 1: Distribution of mutations in 150 genotyped melanoma tumors from sites throughout the body(1).

It should be noted that the current clinical standard of detecting mutations for BRAF mutant melanoma using basic PCR-based assays is suboptimal for many patients (1, 28). The Cobas® 4800 BRAF V600 Mutation Test (Roche Molecular Diagnostics), a PCR-based assay, is the only test currently FDA-approved to aid in selecting patients for vemurafenib therapy. The Cobas® test primarily detects V600E mutations, but has been reported to detect V600K, which is also associated with vemurafenib response. In a comparison between Cobas® and Sanger sequencing in 125 melanoma patients, overall agreement between the methods was only 83.2% (28). The Sanger method had higher analytic sensitivity, resulting in nine additional V600 mutations found in the 57 samples that were negative by the Cobas® test. Thus, 16% (9/57) more patients would be potentially identified as candidates for vemurafenib therapy using the Sanger method. This illustrates that the use of more reliable and robust mutation detection methodology may direct patients to more appropriate therapies and clinical trials, which will ultimately lead to improved outcomes for patients with melanoma.

Molecular Networks as the Drug Target.

It is now firmly established that cancer results from perturbations in the molecular networks within cellular systems that disturb the normal homeostatic state (19, 29, 30). Fluctuations in these networks can result from genetic or epigenetic cellular events and/or changes in the molecular constitution of the tumor microenvironment, which collectively dictate the phenotype of the biological system. The molecular networks engaged during tumor development and/or progression are complex and have evolved to provide the ultimate level of cellular plasticity, allowing cells to adapt to or exploit extracellular cues within the local milieu (31). The complexity of a tumor system is further exaggerated by the inherent genomic instability seen in many neoplasms, which leads to an accelerated micro-evolutionary process that results in further cellular and tumor sub-system heterogeneity (32, 33). This variability combined with the adaptability of many molecular pathways provides a somewhat predictable and highly probable path to resistance for any given agent that targets a subset of cellular systems within a tumor's molecular and genetic repertoire (34). A fundamental challenge in the area of targeted cancer treatment is how to identify optimal therapeutic regimens that can treat heterogeneous tumors that are both highly adaptive and that exhibit significant inter- and intra-patient variation (19-21, 35).

Previous Experience with Personalized Medicine

While the concept of using molecular profiling to direct targeted therapy is in its infancy, studies are beginning to show its impact. A pilot prospective study utilizing molecular profiling of tumor by IHC, FISH, and DNA microarray was completed by our group at the Translational Genomics Research Institute (TGen) (36). This was the first published study utilizing molecular profiling to find potential targets and select treatments accordingly. The study was conducted at 9 sites across the United States and included patients with metastatic cancer who had clear-cut progression of their tumors on their last prior systemic therapy. The primary objective of the study was to compare progression free survival (PFS) using a treatment regimen selected by molecular profiling, versus the time to progression (TTP) observed for the patients' most recent prior treatment regimen, and calculate the PFS ratio (PFS on study/previous TTP). Prior to inclusion in the study, the investigators considered that these patients had no standard treatment available for use. Eighty-six patients were profiled, with a molecular target detected in 84 (98%). Sixty-six patients were treated according to profiling results (breast cancer, n=18; colorectal, n=11; ovarian, n=5; and other miscellaneous types of tumors, n=30). Of the 66 patients treated according to profiling results, 18/66 (27%) patients had a PFS ratio ≥ 1.3 (95% CT, 17-38% one-sided one-sample p=0.007). Broken down by tumor types the PFS ratio was ≥ 1.3 for 44% of the breast cancer patients, 36% of the colorectal cancer patients, 20% of the ovarian cancer patients, and 16% of the patients with miscellaneous types of cancer. In addition to the PFS endpoint, there was 1 complete

response by RECIST, 5 partial responses and 14 patients without progression at 4 months (secondary endpoints for the study). Of particular note was that for the 18 patients with a PFS ratio of ≥1.3, there were no matches for what treatment the patient received based on suggestions from the profiling results and the treatment the physician said she/he would use if profiling results were not available. In most patients with successful tumor molecular profiling, the profiling supported the indication of a new treatment not contemplated initially by the investigator, in a patient population that was heavily pretreated and refractory to previous treatments.

A comparable study conducted at the Van Andel Research Institute (VARI) investigated the utility of gene expression profiling to identify drug targets and associated agents using a range of informatics approaches. A key component of this study was the development of the reporting tools required to convey actionable information back to the tumor board and treating physician (37, 38). Importantly, this approach was granted an Investigational Device Exemption by the FDA in support of a recently launched pediatric neuroblastoma trial (NCT01355679)(39).

Finally, TGen in partnership with US Oncology and the Life Technologies Foundation, undertook a pilot study of NGS (whole genome/transcriptome sequencing) in support of identifying therapeutically actionable targets from 14 patients with triple negative breast cancer (TNBC). This study has helped inform the SOPs selected for this clinical trial.

Data presented at the 2011 ASCO annual meeting by a group at MD Anderson demonstrated that response can be significantly increased when treatment is focused on an identified target (clinical benefit 50% with target vs. 15% without target) (40). Although preliminary, these studies demonstrate that using tumor molecular analysis can successfully direct targeted therapy.

The Stand Up To Cancer/Melanoma Research Alliance Project:
In December 2011, our team was awarded support from the Stand Up To Cancer (SU2C) and Melanoma Research Alliance (MRA) foundations to study whether therapy selection based on systematic integration of large-scale genomic and pharmacopeia information will improve upon the current practice of the physician's empiric choice for treatment of BRAFwt MM. We have been performing deep genomic evaluation of BRAFwt MM to more appropriately define drug therapies and to identify patients for which a target aligns with an existing agent in our portfolio. This analysis will be used in this phase II, statistically-powered clinical trial that will will molecularly characterize the tumors of MM patients and match them with an appropriate agent. Based on the initial 23 patients enrolled on this study who underwent molecular profiling and tumor board evaluation, 20 were assigned MEK162. Therefore, patients who are assigned and receive MEK162 will be evaluable for the primary endpoint of BORR compared to a historical response rate of 7% in this patient population while patients not assigned MEK162 will receive their assigned treatment, but will not be evaluable for the primary endpoint. This approach has the potential to significantly improve clinical outcome for patients with BRAFwt disease, and provide an early example of personalized medicine that can be generally applied to other malignancies. This project represents a fully integrated, clinically defined, and statistically defensible approach to meet head-on the critical challenge of *rapidly* defining molecular alterations in a patient with MM and *rationally* matching this information to experimental clinical treatment. Our cross-disciplinary team includes individuals who are experts in the medical management of patients with MM, drug development, genomics research, biostatistics, bioinformatics, and patient advocacy. This team is uniquely positioned to test our overall hypothesis that a personalized medicine approach to BRAFwt MM will be more efficacious than the historical response rate in empirically selected standard-of-care therapy for this subset of patients who currently have

limited hope for clinical benefit.

The SU2C project outlines an approach by which we can utilize our expanding knowledge of molecular networks and the mechanisms of action of a growing pharmacopeia (41, 42) in conjunction with standardized biomarker assessments to deliver targeted combinations of effective therapies to patients with BRAFwt MM. The emerging high-resolution molecular profiling that defines druggable targets represents a new paradigm in drug development. For our overall SU2C/MRA project, the current standard of investigating drug efficacy based solely on histopathologic classification will be challenged. Instead, we will investigate drug efficacy against tumors defined by the tumors' molecular/genetic profiles. BRAFwt MM patients have an enormous unmet need for adequate treatment options and represent an ideal population for investigating the utility of personalized target/therapy identification. Even with the prospect of additional genomic information from large-scale retrospective studies of melanoma (e.g. The Cancer Genome Atlas, or TCGA), remarkably little information will be available to correlate the genomic make-up of mutationally-defined subtypes of disease with clinical outcome. Although not the primary aim of our SU2C/MRA project, we will unveil significant genomic information relative to BRAFwt MM; we anticipate these data will define new targets for future drug discovery efforts. The studies outlined herein represent a step in the development of personalized oncology, in which each patient is truly treated individually based upon the systematic molecular analysis of their disease. We believe the solid foundation built through the conduct of the SU2C/MRA project will begin to not only define appropriate therapeutic interventions for the BRAFwt MM patient population, but will also make significant strides in changing the treatment paradigm for all cancer patients.

To test our hypothesis, we will test the novel strategy of matching BRAFwt MM patients with the most appropriate molecularly guided therapy in a large-scale, statistically-powered, phase II clinical trial. For future clinical trials, outcomes from the phase II study will feed back into the statistical and informatics methodologies, to subsequently test and refine the predictive models.

SU2C Pilot Study:

Discussions with the United Stated Food and Drug Administration (FDA) regarding regulatory guidance for the Stand Up To Cancer project were held in a pre-IND, "Type B" teleconference on July 20, 2012. These discussions led to the decision to conduct an initial small-scale, non-treatment pilot study of the Stand Up To Cancer project. This study began enrolling patients in November 2012, and using a small sample set of five BRAFwt MM patients, has assisted us in identifying potential issues and refining the standard operating procedures (SOPs) prior to initiation of the subsequent large scale phase II statistically-powered efficacy trial. Most aspects of the pilot trial mirror the design of the subsequent large-scale, statistically powered study. However, the 5-patient pilot study was not randomized and did not involve treatment of patients. It did, however, allow us to evaluate every step in real time, alert us to potential adverse issues, and design the powered study as efficiently as possible. Of particular importance, this pilot study assisted us in developing a framework for therapeutic decision-making.

Feasibility for the pilot study was assessed based upon the completion of the following within a 5-week period: tumor biopsy; tissue pathologic evaluation, DNA/RNA extraction and quality control, molecular profiling, identifying DNA mutations and copy number alterations, RNA sequence and expression level alterations; integration of DNA and RNA information; knowledge mining and report generation; tumor board review with formulated treatment plan; and independent medical overseer review.

Clinical tissue custody, pathology evaluation, analyte generation, validation of prioritized biomarkers, and Next Generation Sequencing (NGS) of DNA/RNA occurred at TGen. This information was analyzed and a report of identified drug targets and matching therapeutics provided to a multi-disciplinary tumor board to construct a mock treatment plan for each patient from the same pre-assembled portfolio of targeted therapeutic agents (both FDA approved and investigational agents) that have been selected for consideration for treatment of patients in the large-scale trial (Appendix II). Enrollment to this pilot study has concluded.

Rationale for the Phase II SU2C study:
The vemurafenib experience demonstrates that the efficacy of specific treatment modalities is dependent upon the molecular constitution of the tumor, and that the observed variations in tumor response to current therapies are largely attributable to disease heterogeneity at the molecular level.

A different approach to the management of patients with advanced BRAF wild-type metastatic melanoma is needed in order to identify effective treatments from the catalog of available agents. Advances in informatics and molecular technologies, coupled with our expanding knowledge of molecular networks and mechanism of action of the existing pharmacopeia, provide a great opportunity in translational medicine to develop a model that more accurately predicts tumor response. The clinical evaluations in the SU2C/MRA project, beginning with the pilot study and moving into the Phase II clinical trial, represent a step in the development of personalized oncology, in which each patient is truly treated individually based upon the systematic molecular analysis of their disease.

This large-scale study will use a specific data-driven predictive method through which any number of drugs in our current knowledge base has a chance of being recommended; of these, both relevant FDA-approved and investigational agents will be used. By tailoring therapy with drugs targeting the specific molecular composition of a tumor—irrespective of tumor classification or anatomical origin— we will provide an alternative to the conventional approach that targets specific organs or tissues without consideration of the underlying tumor biology.

Initially, the study was designed to involve a 2:1 randomization of molecularly-guided therapy vs. Physician’s Choice (standard-of-care). However, based on the initial 23 patients enrolled on this study who underwent molecular profiling and tumor board evaluation, 20 were assigned MEK162. Therefore, to reduce the necessary patient and financial resources needed to carry out this clinical trial, the study has been redesigned to statistically evaluate the primary endpoint of BORR in patients assigned MEK162 compared to a historical response rate of 7%. Patients not assigned MEK162 will receive their assigned treatment, but will not be evaluable for the primary endpoint.

We propose that the sequencing of a patient’s cancer versus sequencing of their normal cells could enable a “deep dive” analysis of the patient’s cancer genomes. Coupled with innovations in knowledge engineering, such an analysis could provide sufficient depth and dimensionality of genomic measurements to properly interpret the molecular context of vulnerability to the point where we may be able to understand the key oncogenic dependencies of an individual’s disease. It’s this understanding, at an individual level, irrespective of other cases, that forms the foundation of our individualized approach called ‘Personalized Medicine’.

Patients with BRAFwt metastatic melanoma have a short life expectancy because existing therapies are minimally effective and the mutations that drive the growth and survival of this disease are poorly understood. An understanding of the molecular underpinnings of BRAFwt metastatic melanoma is urgently needed to advance the development of rational therapeutics

for this disease. Critical to the success of the innovative translational study will be the creation of a framework for capturing, processing, analyzing, mining, and interpreting the data. The pilot study demonstrated that this approach can give information on patients' tumors, and shows that we can provide actionable information to the treating physician which can eventually guide cancer therapy. We now aim to conduct the phase II clinical trial and will utilize our pre-assembled portfolio of molecularly guided therapeutic agents, along with the SOPs developed in the pilot study, combined with our refined molecularly-guided decision tree. We hypothesize that the use of molecularly-guided therapy will improve overall tumor response in those patients assigned to MEK162 compared to historical rates. This clinical trial will be conducted in a similar fashion to the pilot trial. However, it will take advantage of the knowledge and refinement identified through the completion of that pilot trial, will involve actual treatment of the patient, and will be performed on a much larger scale to fulfill the requirements to achieve the necessary statistical power to test our hypothesis.

6 Eligibility to Participate

6.1 *Inclusion Criteria*

NOTE: Unless specified, these criteria will be used to evaluate patient eligibility for initial enrollment in the study (prior to biopsy and genomic analyses). Specific criteria can and will be used for all treatments following treatment selection, based on the particular drug selected for the individual patient. If any conflicts exist between the general protocol inclusion/exclusion criteria presented here, or the inclusion/exclusion criteria presented in the drug-specific appendices, the criteria in the drug-specific appendices take precedent.

6.1.1 Patients with metastatic or locally advanced and unresectable BRAF wild-type melanoma who have either progressed following previous treatment of immunotherapy, or are not eligible for immunotherapy. Patients are defined as "BRAF wild-type" if they test negative for V600 mutations based on a CLIA certified assay**.**

6.1.2 Patients must have tumor accessible by interventional radiology or surgical intervention and suitable for biopsy with 5-6 passes of a 16 or 18 gauge needle for core biopsy (defined as at least 1 cm^3 tumor/50 mg accessible for biopsy), and must agree to undergo up to two surgical resections/biopsies to collect tumor for research purposes. The first of these biopsies will occur at the beginning of the study, prior to genetic analysis and treatment. The second biopsy will be performed at the time of disease progression/end of study should funding be available. See section 8.1 tumor and blood collection

6.1.3 Patients must have measurable disease (per RECIST v1.1 criteria), defined as at least one lesion that can be accurately measured in at least one dimension (longest diameter to be recorded for non-nodal lesions and short axis for nodal lesions) as ≥20 mm with conventional techniques or as ≥10 mm with spiral CT scan, MRI, or a subcutaneous or superficial lesion that can be measured with calipers by clinical exam. For lymph nodes, the short axis must be ≥15 mm. See Section 13 for the evaluation of measurable disease.

6.1.4 Previous therapies: Prior radiation therapies, immunotherapies, and investigational therapies are allowed as follows.

6.1.4.1 Radiation: Prior radiation therapy is allowed with the following conditions:

- Patients who have received minimal radiation therapy (≤5% of their

total marrow volume) must have completed it ≥ 2 weeks prior to the initiation of study treatment.

- Patients who have received radiation therapy that constituted >5% but < 50% of their total marrow volume must have completed it ≥ 4 weeks prior to the initiation of study treatment.
- Patients who have received prior radiation to 50% or more of their total marrow volume will be excluded
- Patients may be biopsied while undergoing radiation therapy as long as biopsy site is not in the radiation portal. However, they still have to wait the required amount of time from radiation to treatment even though the tumor board may have already occurred and a treatment plan assigned.

6.1.4.2 Other therapies: Prior investigational or targeted therapies and immunotherapies may be allowed following discussion with the Principal Investigator. If the PI deems the prior treatment acceptable, patients must not have received these therapies for 28 days or five half-lives of the drug (whichever is lesser) prior to the initiation of study treatment and must have full recovery from any acute effects of these therapies. Prior therapy with MEK inhibitors will not be allowed.

6.1.5 Patients with chronic grade 2 toxicity may be eligible at the discretion of the Principal Investigator if the condition has been stable, and not worsening, for at least 30 days. Patients with ongoing alopecia of any grade will be eligible. (See Exclusion 6.2.1 regarding criteria for peripheral neuropathy)

6.1.6 Patients must be ≥ 18 years of age. Because no dosing or adverse event data are currently available for patients <18 years of age for many of the agents in the study pharmacopeia, children are excluded from this study.

6.1.7 Patient must have a life expectancy of ≥3 months, as estimated by the treating oncologist

6.1.8 Patient must have an Eastern Cooperative Oncology Group (ECOG) performance status ≤2 (see Appendix I).

6.1.9 Patients must have adequate organ and marrow function as defined below.

6.1.9.1 Hemoglobin ≥ 9 g/dL
6.1.9.2 Leukocytes ≥ 3,000/microliter (mcL)
6.1.9.3 Absolute neutrophil count (ANC) ≥1,500/mcL
6.1.9.4 Platelets (PLT) ≥100,000/mcL
6.1.9.5 Aspartate aminotransferase (AST) ≤ 2.5 x upper limit of normal (ULN); if liver metastases are present, ≤ 5 x ULN
6.1.9.6 Alanine aminotransferase (ALT) ≤ 2.5 x ULN; if liver metastases are present, ≤ 5 x ULN
6.1.9.7 Bilirubin ≤ 1.5 x ULN
6.1.9.8 Creatinine ≤ 1.5 x ULN
OR:
Calculated or measured creatinine clearance ≥ 50 mL/min/1.73 m^2 for patients with creatinine above institutional normal

6.1.10 If available, patient must agree to provide archival tissue (collected prior to

immunotherapy whenever possible) for research purposes (either archival paraffin tissue block or 10 unstained slides of a primary or metastatic melanoma lesion) prior to enrollment. Samples should be shipped within 3 months after enrollment.

6.1.11 Patient agrees to having a blood sample (approximately 10 - 20 mL) drawn and analyzed to compare their normal genetic profile to that of their tumor sample

6.1.12 Patient must be able to tolerate oral medication.

6.1.13 The effects of investigational agents/chemotherapy on the developing human fetus are unknown. For this reason and because therapeutic agents or modalities used in this trial are known to be teratogenic, women of child-bearing potential and men must agree to use 2 forms of adequate contraception (hormonal or barrier method of birth control; abstinence) for the duration of study participation, and for four months following completion of study therapy. Should a woman become pregnant or suspect she is pregnant while participating in this study, she should inform her treating physician immediately. Women who become pregnant must immediately discontinue treatment with any study therapy. Male patients should avoid impregnating a female partner. Male patients, even if surgically sterilized, (i.e. post-vasectomy) must agree to one of the following: practice effective barrier contraception during the entire study treatment period and through 4 months after the last dose of study drug, or completely abstain from sexual intercourse.

6.1.14 Patient must have the ability to understand and the willingness to sign a written informed consent document.

6.1.15 Patient must be willing and able to comply with the protocol for the duration of the study, including attending scheduled visits, examinations, the biopsy procedure, and having their tumor and blood molecularly characterized.

6.1.16 Patient understands they must meet all Inclusion and Exclusion criteria in the drug specific appendix for which they were assigned.

6.2 *Exclusion Criteria*

6.2.1 Patients with peripheral neuropathy ≥ Grade 2 are not permitted unless discussed with the Principal Investigator and only in unique circumstances (i.e. unilateral neuropathy due to trauma)

6.2.2 Patient has disease that tests positive for BRAF V600 mutations based on the results of a CLIA certified assay.

6.2.3 Patients with active infection (unless discussed with PI)

6.2.4 Patients with any evidence of severe or uncontrolled systemic disease(s) including known cases of Hepatitis B or C or human immunodeficiency virus (HIV). Screening for chronic conditions is not required, although patients known to have such conditions at screening should not be included.

6.2.5 Any patient requiring chronic maintenance of red blood cell, white blood cell or granulocyte counts through the use of blood transfusions or growth factor support (e.g. Neulasta®, Neupogen®)

6.2.6 Patients with a prior history of seizures within the past year unrelated to brain metastases.

6.2.7 Patients with known active progressive brain metastases. (These patients will be excluded from this clinical trial because of their poor prognosis and because they

often develop progressive neurologic dysfunction that would confound the evaluation of neurologic and other adverse events.) Patients with prior treated brain metastases are allowed, providing that they were not accompanied by seizures within the past year and that a baseline brain MRI scan prior to study entry demonstrates no current evidence of active brain metastases. All patients with prior treated brain metastases must be stable for >1 months after treatment and off steroid treatment prior to study enrollment.

6.2.8 Patients receiving any other anti-cancer therapy (cytotoxic, biologic, radiation, or hormonal other than for replacement) except for medications that are prescribed for supportive care but may potentially have an anti-cancer effect (i.e. megestrol acetate, bisphosphonates). These medications must have been started ≥ 1 month prior to enrollment on this study. Patients may be on low molecular weight heparin or direct factor Xa inhibitors.

6.2.9 Patients with any clinically significant medical condition which, in the opinion of the Investigator, makes it undesirable for the patient to participate in the study or which could jeopardize compliance with protocol requirements including, but not limited to: ongoing or active infection, significant uncontrolled hypertension, or severe psychiatric illness/social situations

6.2.10 Patients with preexisting cardiac conditions, including uncontrolled or symptomatic angina, arrhythmias, or congestive heart failure will not be eligible.

6.2.11 Patients with LVEF < 45% will not be eligible

6.2.12 Patients with either of the following within 6 months before the first dose of study treatment:

6.2.12.1 stroke (including TIA, or other ischemic event)
6.2.12.2 myocardial infarction

6.2.13 Patients with acute gastrointestinal bleeding within 1 month of study entry.

6.2.14 Patients who have, at screening, QTcF ≥450 msec for males and QTcF ≥470 for females

6.2.15 Patients with a co-morbid condition(s) that, in the opinion of the investigator, prevents safe surgery/biopsy procedure.

6.2.16 Patients with malabsorption syndrome or other condition that would interfere with intestinal absorption or ability to swallow oral medication.

6.2.17 Pregnant or nursing women. These women are excluded from this study because of the potential for being treated with an agent having teratogenic or abortifacient effects. There is also potential risk for adverse events in nursing infants secondary to treatment of the mother with agents included in the pharmacopeia. As a result, breastfeeding must be discontinued prior to treatment.

6.2.18 Patients who have received organ transplant

6.2.19 Patients who have had major surgery within 14 days of study enrollment.

6.2.20 Patients diagnosed or treated for another malignancy within 3 years of enrollment, with the exception of complete resection of basal cell carcinoma or squamous cell carcinoma of the skin, or an *in situ* malignancy. Patients with low grade prostate cancer, not on hormonal therapy, for which the disease is confined to the prostate,

may be considered eligible by the overall Principal Investigator on a case by case basis.

6.3 *Inclusion of Women and Minorities*

Women and men of all races and ethnic groups are eligible for this trial.

7 Registration Procedures

7.1 *General Guidelines*

Eligible patients will be entered on study centrally at Yale Center for Clinical Investigation by the Coordinating Site Project Manager (CSPM) Manuel Avedissian, or appropriate designee from the Principal Investigator. Study slots will not be limited to a predefined number for a particular clinical site. Sites will accrue based on total slot availability. Clinical sites should notify the CSPM Manuel Avedissian by email at manuel.avedissian@yale.edu with the screening date for potential patients. If the screening date is not known in advance the clinical site should notify the CSPM the day of screening. Nearing the end of recruitment or at any other time deemed necessary by the Principal Investigator, clinical sites may be required to request a slot before screening a patient. Sites will be notified when this occurs and given the process for requesting a slot.

All patients (or patients' legal representatives) must provide written informed consent before any study specific assessments may be performed. Following consent, attempt should be made for patients to have all screening tests and the biopsy/blood collection performed within 7 days unless a site contacts the CSPM or designee about the delay and the site receives an email confirmation from the CSPM or designee approving delay. Issues that would cause screening and specimen collection delays should be discussed with the Principal Investigator.

If the patient fits all enrollment criteria, the site will again contact the CSPM for official enrollment confirmation and unique patient identifier assignment. All appropriate registration forms must be faxed or emailed to the CSPM, as noted in Section 7.2 below. If a patient does not receive tissue/blood collection following registration, the patient's registration on the study will be counted as 'registered' but not included in determination of appropriate objectives or endpoints and will be replaced. The CSPM should be notified of cancellations as soon as possible. If a patient does not receive tissue/blood collection following registration, the patient may be replaced for appropriate endpoint evaluation.

Except in very unusual circumstances, each participating institution will place the initial order of investigational agents and commercially available agents provided by the study via the pharmacy at the Coordinating Site. Following initial IRB approval the participating institution should contact the Oncology Investigational Drug Service at Smilow Cancer Hospital at (203)-200-4455 or email (preferred) PharmacyOncInvDrug@ynhh.org. The initial drug shipment will include enough drug for 2 patients for 2 cycles. Subsequent treatment cycles of drug provided by the study will be ordered directly from the participating clinical site to the applicable company or designee. Agents may be ordered by a participation site only after the initial IRB approval for the site has been forwarded to the Coordinating Site.

7.2 *Registration Process*

To register a patient, the following documents should be completed by the research nurse or Responsible Data Manager at a participating site and faxed (203-785-3500) or e-mailed (manuel.avedissian@yale.edu) to the Coordinating Site Project Manager:

- Copy of required laboratory tests
- Signed patient consent form
- Signed HIPAA authorization form
- Eligibility Screening Worksheet (signed)
- Registration Form (signed)
- Pathology report including BRAF V600 Mutation Test Results
- Source documents to confirm all eligibility criteria

The research nurse or data manager at the participating site will then call (203-737-3669) or e-mail Manuel Avedissian (manuel.avedissian@yale.edu), the Coordinating Site Project Manager to verify eligibility. To complete the registration process, the Coordinating Site Project Manager will:

- confirm eligibility
- assign a patient study number
- register the patient on the study
- fax or e-mail the patient study number to the participating site

8 Study Design

This is a Phase 2, prospective, multi-center, open-label study with a Simon 2-Stage optimal design.

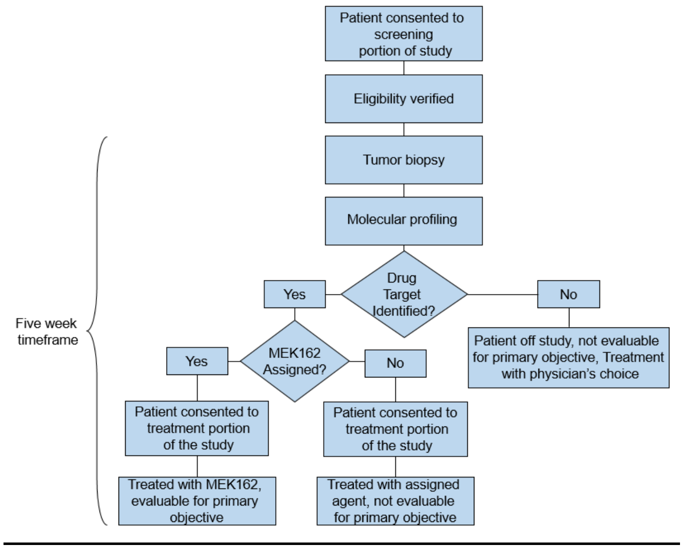

Figure 2. Study schema

The study is summarized as follows (Figure 2). Patients with metastatic BRAFwt MM whose tumors have progressed following previous treatment with standard therapy (e.g. immunotherapy) or for which immunotherapy was not given due to co-morbidities, patient refusal, etc. will undergo initial evaluation for eligibility. Following consent, they will undergo screening tests as well as a tumor biopsy and blood draw. Specimens from all sites will be managed and sent to Ashion Analytics, LLC, a central CLIA-certified laboratory affiliated with TGen, for study pathology evaluation and molecular analyte generation. The tumor sample will be retained under a "chain of custody" within Ashion Analytics. Separate tumor samples from a subset of patients will be sent to the Mayo Clinic for the establishment of primary patient tumorgrafts in immune compromised mice.

For each qualified tumor (DNA/RNA) and blood (DNA) sample, extracted analytes will be sent from Ashion Analytics to an adjacent laboratory at TGen for Next Generation Sequencing (NGS). Data obtained will be processed by the Genome Processing and Knowledge Recovery Teams, and a list of mutations will be generated. The molecular data will be presented to a molecular tumor board who will assess the molecular profile

generated for the individual patient and analyze the weight of evidence in support of a proposed target/drug match. This information will then be discussed by a clinical tumor board who will determine an individualized treatment plan based on the available data. If a druggable target/pathway is found, the results will be verified by Ashion Analytics as needed. The formulated treatment plan will be reviewed by an independent medical overseer and additional screening will occur to ensure the patient is eligible for the selected treatment. Based upon our group's participation in similar trials, we anticipate the time from patient biopsy to initiation of treatment to be less than 5 weeks. Extent of disease will be initially measured at 30-35 days, and at 8 or 9 week intervals (depending on length of cycle) following initial assessment. After 6 months of continued therapy, scan frequencies can be altered to reflect routine care. In the event profiling does not identify a therapy, the patient elects not to be treated, or there are co-morbidities excluding them from undergoing therapy, the sequence of events will be documented and the patient will be discontinued from the study, and be replaced.

The aim of this study is to evaluate whether molecularly-informed personalized therapy selection, based on a patient's tumor molecular profile, will improve outcomes when compared to the historical response rate seen in standard-of-care therapy in patients with BRAFwt metastatic melanoma. Patients who are assigned and receive MEK162 will be evaluable in the primary endpoint of Best Overall Response Rate (BORR) compared to a historical response rate of 7% in this patient population. Patients not assigned MEK162 will receive their assigned treatment, but will not be evaluable for the primary endpoint.

The primary objective, comparison of best overall response rate between patients assigned MEK162 following molecular analysis of their tumor, will be assessed by the presence of radiologically assessable disease before and after treatment and compared to a historical response rate of 7%. To select treatment, we will characterize BRAFwt metastatic melanoma tumors to identify a molecularly informed list of mutations. A 5-week timeframe will be followed from biopsy to initial treatment and includes the following, as detailed below and schematically in Figure 2:

- tumor biopsy;
- tissue pathologic evaluation
- molecular analysis (DNA/RNA extraction and quality control, molecular profiling, identifying DNA mutations and copy number alterations, RNA sequence and expression level alterations, integration of DNA and RNA information, knowledge mining and report generation)
- molecular and clinical tumor board reviews with formulated treatment plan;
- independent medical overseer review;
- first treatment with molecularly-guided therapy

8.1 *Study Workflow*

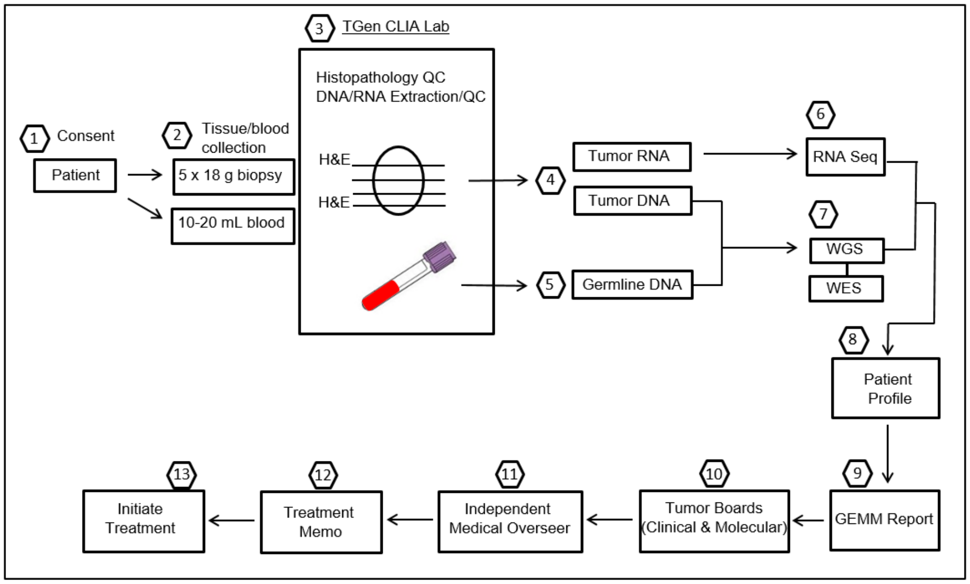

Figure 3: Process diagram depicting key steps from patient consent, through molecular profiling and review by the medical overseer, to patient treatment. The textual summaries relate to the referenced numerical ID.

Figure 3 depicts the summary workflow of the approach we will take to align molecular profiles of individual patients and their biopsied disease with molecularly-guided therapy. The estimated time from patient biopsy to initiation of treatment is 35 days, and includes a 7-day variance factor that we have incorporated to account for unexpected delays as well as non-business days. The sections below summarize the individual enumerated steps in the process.

1. <u>Patient Informed Consent</u>
The use of human specimens for research has never been under stricter ethical scrutiny, because of the wealth of personal, clinical, and behavioral information that can be extracted from such samples. We recognize that research involving biospecimens and genetic analyses raise important issues concerning informed consent. Although the collection, storage, and research use of biospecimens and data are typically thought to involve minimal risk (43), information must be conveyed during the consent process that can be complex or controversial. Examples include the use of biospecimens; medical record access; contact for future research; large-scale data sharing; development of commercial products; privacy and confidentiality protections; participants' access to research results; and the ability to discontinue participation.

Recognizing appropriate informed consent underpins the ethical use of human specimens in research, we fully intend to conduct all operations of this specimen bank in a way that maximally protects the interests of the participant as well as future patients. Outlined below is the code of conduct we intend to implement to best balance the needs and rights of patients and researchers and optimize the use of this precious resource.

For research as part of this protocol, written informed consent will be obtained from each specimen donor in accordance with HHS regulations noted in 45 CFR 46.116 (General requirements for informed consent). Included among the basic elements of informed consent will be a clear description of the following:

- study goals

- methods used and risks involved, including a discussion of the biopsy process and how both tumor and germline mutations will be identified and what disclosure of these results may mean to study participants and their families. The experimental nature of molecular sequencing will be highlighted.
- specific description of the type of research to be conducted
- conditions under which data and specimens (including all derivatives such as derived tumorgraft models) will be released to recipient-investigators
- procedures for protecting the privacy of subjects and maintaining the confidentiality of data
- basic operation of the specimen storage at TGen in Arizona and length of time the specimen will be stored at TGen

Potential study subjects will be encouraged to ask questions. The voluntary nature of the study will be stressed, as well as the ability to withdraw from participation. At the time of consent, appropriate information about what will be done with the samples will be provided to the patient.

2. <u>Tumor and blood collection</u>
Following registration, each patient will undergo a blood draw for research and a scheduled surgical or biopsy procedure. The patient will be given a full description of the biopsy/blood draw procedures and associated risks. Common sample identifiers will be provided by the Study Coordinator and will be used by all parties throughout the project to ensure sample, data and report alignment between participating organizations.

For each biopsy, we will collect five to six 1-2 centimeter 16- or 18-gauge core needle specimens from accessible tumor. Two of the cores will be placed in externally-threaded cryovials and immediately flash frozen in liquid nitrogen and stored at -80°C until shipping on dry ice. Both of these frozen cores will be shipped to Ashion Analytics a central CLIA-certified laboratory affiliated with TGen in Arizona for DNA and RNA extraction (see Appendix IV). Depending on need, a third core will either be flash frozen and sent to Ashion or TGen for correlative analyses or biobanking (see Appendix IV), or it will be placed immediately in RPMI media and shipped to Mayo Clinic in Arizona on frozen and refrigerated cool packs for implanting into immune compromised mice for primary tumorgraft development (see Appendix V). The fourth core will be cryopreserved in Optimal Cutting Temperature (OCT) compound for proteomics (to be sent to George Mason University; see Appendix VIII). A fifth core will be formalin-fixed and paraffin-embedded at the clinical site and the block shipped to Ashion (see Appendix VI). Archival (diagnosis) paraffin blocks or slides (archival tissue collected prior to immunotherapy whenever possible) will also be shipped to Ashion (within 3 months of patient enrollment) for longitudinal analyses (see Appendix VI). Additional tissue, if any is available, will be flash frozen and banked at Ashion.

For surgical biopsy, a minimum of 50 mg of tissue will be collected and partitioned to reflect the distribution of the core biopsies described above.

Order of fresh tissue priority (<u>**initial biopsy**</u>):
1. Flash frozen core for DNA analysis (to be sent to Ashion; see Appendix IV);
2. Flash frozen core for RNA analysis (to be sent to Ashion; see Appendix IV);
3. Flash frozen core for correlative analyses or biobanking (to be sent to Ashion; see Appendix IV) <u>**OR**</u> fresh core biopsy in transport media (NOT FROZEN) for

tumorgraft development (to be sent to Mayo Clinic in Arizona). Exact use of this core will be based on need and determined by the Principal Investigator for each individual patient enrolled on this study (see Appendix V);

4. Cryopreservation of Tissue in OCT for proteomics (to be sent to George Mason University; see Appendix VIII)
5. Core for creation of formalin-fixed paraffin-embedded (FFPE) block (to be fixed/embedded at clinical site and sent to Ashion; see Appendix VI)
6. Any extra tissue will be flash frozen and biobanked (to be sent to Ashion; see Appendix IV)

Each patient will also have blood collected at the time of initial biopsy and/or while on treatment as follows:

1.) All patients will have 10-20 mL of whole blood collected at the time of biopsy in lavender top EDTA tubes for extraction of constitutional analytes at Ashion (see Appendix III).
2.) All patients will have 10 mL of blood collected at the following timepoints: day of initial biopsy (prior to actual biopsy procedure); Cycle 1, Day 1 (prior to treatment); Cycle 1, Day 8 or 15 (depending on which date their scheduled visit will occur, prior to treatment); and Day 1 of each subsequent cycle (prior to treatment) for preparation of plasma for analysis of circulating tumor nucleic acids (ctNA) at TGen (see Appendix VII)
3.) All patients will also have 5.5 mL of blood collected at the following timepoints: day of initial biopsy (prior to actual biopsy procedure); Cycle 1 Day 15 or Cycle 2 Day 1 (depending on cycle length, prior to treatment); and at the time of clinical response and/or progression for RNA gene expression profiling and assays at Merck (see Appendix IX).

Patient identifiers will remain under HIPAA compliance, and clinical information will be kept in accordance with each site’s standard practice. Sample kits containing barcoded tubes for shipping samples to the appropriate laboratory will be provided.

Appendix X contains a table summarizing sample distribution from the clinical sites. Appendices III, IV, V, VI, VII, VIII and IX contain detailed standard operating procedures for the sample procurement, handling, processing, and shipping procedures for blood and tissue. Figure 4 depicts the planned specimen processing and workflow for the first biopsy and during treatment.

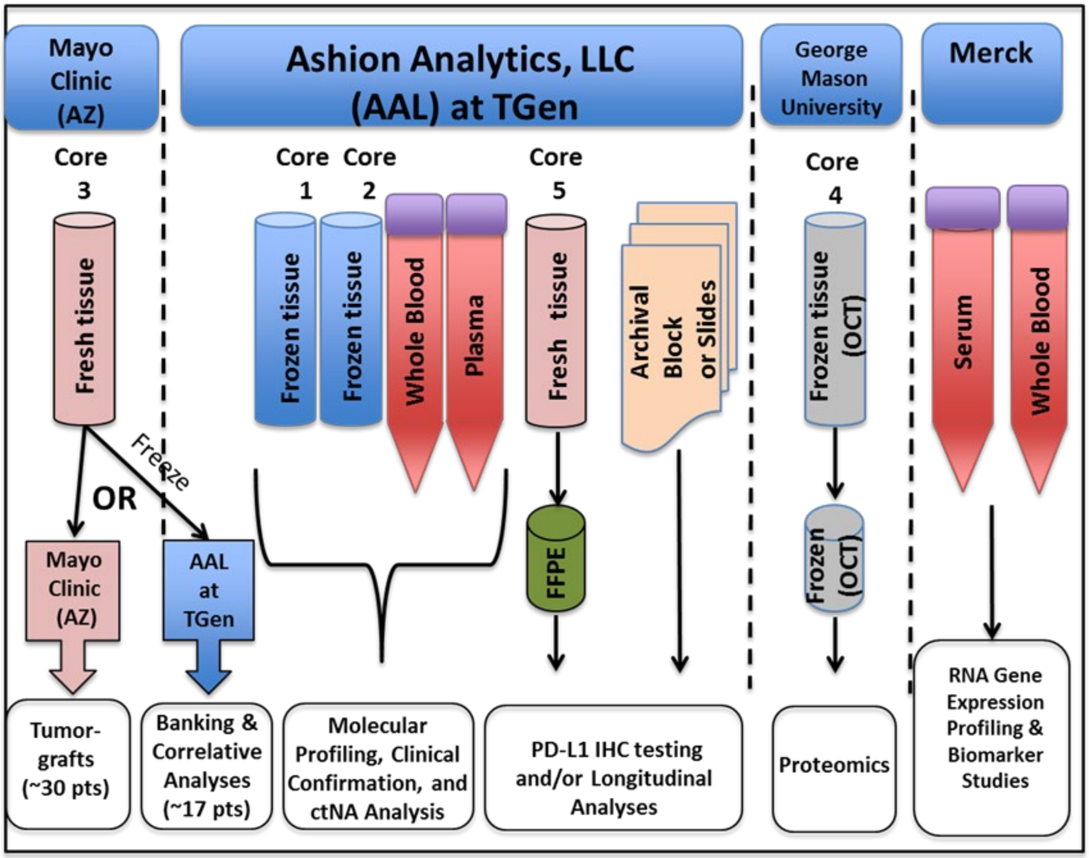

Figure 4: Specimen Processing and Workflow (first biopsy and during treatment)

3. <u>TGen CLIA lab Receipt and Pathological QC</u>
Upon receipt by the Ashion Analytics staff, each sample will be accessioned, verified against the shipping manifest, and entered into a Laboratory Information Management System (LIMS). The sample will be transferred on dry ice following Chain of Custody procedures to the frozen QC area and a weight of the tissue will be taken and recorded in the LIMS. The tissue will be inked with black and red pathology ink on polar opposite ends of the tissue. A small sliver of tissue will be chipped off of each colored end, not to exceed 10% of the whole sample. The frozen tissue will then be weighed and the value recorded in the LIMS as the "post QC weight". The two tissue chips will be formalin-fixed and paraffin embedded, and 4 μM sections will be cut from the tissue blocks and stained with hematoxylin and eosin (H&E). Samples will be qualified for the study based on pre-established histopathological acceptance criteria. The H&E tissue sections will be reviewed by a board-certified pathologist to identify samples containing ≥ 25% tumor nuclei, and DNA and RNA will be extracted from qualified samples. The histopathological QC results will be entered in the LIMS, and the sample will be triaged for analyte isolation.

4. <u>Extraction and QC of genomic DNA and total RNA from tumor tissue</u>
Ashion Analytics will extract DNA and total RNA from the two full-core frozen tissue samples they receive. DNA and RNA will be quantitated by spectrophotometry and the distribution of molecular weights in the preparations will be visualized by gel electrophoresis. RNA integrity will be assessed using the Agilent Bioanalyzer. DNA

samples will be aliquoted and kept at -20°C, and RNA samples will be aliquoted and kept at -80°C until use in molecular studies.

5. Extraction and QC of genomic DNA from whole blood.
Whole blood samples will be immediately sent to TGen for extraction of DNA using the Qiagen All Prep Kit. DNA will be quantitated by spectrophotometry, and the distribution of molecular weights in the preparations will be visualized by gel electrophoresis. Blood-derived DNA samples will be aliquoted and kept in Ashion Analytics at -20°C until use in molecular studies.

6. RNA Sequencing (RNA Seq) of tumor total RNA to identify transcriptome aberrations.
RNA-Seq will be performed on the tumor sample only using paired-end chemistry on the Illumina HiSeq instrument and >100 base pair read lengths. Our RNA-Seq approach will focus on messenger RNA only and we will dedicate at least 50 million unique reads to each sample. RNA-Seq data will be used for several purposes (where applicable) based on expression level of the gene/transcript): (1) to improve our confidence in any detected gene-gene fusion events; (2) to improve our confidence in any detected mutation event; (3) to compare the transcription profile to a collection of profiles from normal tissues in order to identify the set of transcripts that most readily highlight a therapeutically actionable approach; (4) to compare the transcription profile to a collection of profiles from other tumor samples to assess the similarities and differences; and (5) to compare the transcription profile to a collection of profiles from other melanoma BRAFwt tumor samples to identify transcripts and pathways that are significantly similar.

7. Whole genome sequencing of both germline and tumor DNA to identify additional candidate DNA aberrations.
Whole-genome Sequencing (WGS) will be performed using two approaches: WGS using a shotgun long insert approach for detection of structural variants and exome sequencing for identification of mutations within exons. The whole-genome sequencing libraries will be designed for detection of structural variants, such as copy number variants or translocations. We will leverage paired-end sequencing chemistry on the Illumina HiSeq instrument with a typical insert size of between 800-1,200 base pairs, and read-pairs will contain greater than 50 bases of Q30 data as measured by the sequencer. We will sequence both the tumor- and normal-derived DNA within a lane utilizing sequencer of primer specific barcodes to bin reads to the appropriate source library. A target of greater than 20x physical coverage will be generated by use of long-insert libraries sequenced for approximately 100 million read-pairs. WGS is included in our study design because we can leverage the resulting data to determine copy number changes with very high resolution and can detect gene-gene fusion events with accuracy.

A second form of whole genome sequencing will also be utilized – Whole Exome Sequencing (WES), or deep coverage exome sequencing (Exome-Seq). Exome-Seq utilizes a capture strategy to target and enrich for regions within the human genome that are known to encode for expressed sequences (e.g. exons). We will perform Exome-Seq using standard paired-end chemistry on the Illumina HiSeq instrument and insert sizes with read lengths of >100 base pairs. The tumor sample will be sequenced to an average depth per targeted base pair of at least 100X, while the normal sample will be sequenced to an average depth of at least 50X. Utilizing deep Exome-Seq will allow us to search for and identify mutations that are present in the tumor sample even

if the heterogeneity is high. For example, in a sample that is 50% tumor, for the "average" sequenced base pair we would still expect to see 50 out of 100 sequence reads representing a homozygous change in the tumor or 25 out of 100 sequence reads for a heterozygous change. These levels are significantly above the typical overall error rate of next generation sequencing (currently between 1-4%). Deep Exome-Seq is included in our study design to allow us to search for tumor specific mutations in known oncogenes and tumor suppressors AmpliSeq assay. Additionally, Exome-Seq will allow us to search for novel tumor mutations outside of these known genes.

8. Patient Profile

The next generation sequencing approaches employed in the Genomics-Enabled Medicine for Melanoma study will identify somatic events from normal/tumor pairs at the genomic (DNA) level, including coding point mutations and small insertions/deletions, copy number changes, and structural events (intra-chromosomal rearrangements and translocations), and at the transcriptomic (RNA) level including differential gene expression and RNA fusions. These aggregate data sets will be curated and annotated by the Genome Processing and Knowledge Recovery Team at TGen, and a molecular profile will be generated for each patient. This molecular profile will be collected along with the patient's histopathology information and clinical history to form a patient profile. As described in more detail below, this patient profile will be used to generate a Genomics-Enabled Medicine for Melanoma (GEMM) report, which will be shared with the Molecular Tumor Board who will assess the weight of genomic evidence in support of particular target/drug matches.

Investigational agents in the study pharmacopeia were selected under the auspices of the assembled SU2C team under the direction of Dr. Patricia LoRusso. Selection criteria included: mechanisms of action relevant to non-BRAF mutated melanoma biology; previous knowledge of drug and possible preclinical and clinical efficacy; prior determination of a recommended Phase 2 dose (RP2D) and/or maximally tolerated dose (MTD); and drug availability. Approved oncology agents were selected from broad therapeutic classes based on information available from the Melanoma Disease Model initiative from Cancer Commons and the Melanoma Molecular Map Project. Target/drug matching is performed through curation of PubMed biomedical database and publications to identify those molecular events that alter drug response. The gene/drug annotation framework is based on the Targeted Therapeutic Database maintained by the Melanoma Molecular Map Project. The supporting evidence comes from a variety of sources including PubMed, clinicaltrials.gov, and DrugDex (Thomson Reuters). While the total drug pool available to this study using this approach is currently 326 FDA approved drugs, only those that overlap with the drug list being evaluated in this clinical trial will be considered by the Molecular and Clinical Tumor Boards.

9. Generation of a Genomics-Enabled Medicine for Melanoma (GEMM) Report

Upon execution of the different genomics analytical methodologies in Nodes 6 and 7 and creation of a patient profile in Node 8, a knowledge mining analysis and translational report (termed a "Genomics-Enabled Medicine for Melanoma Report") will be generated for each patient. Each report will provide an interpretation of the genomic aberration, an evidence-supported mechanistic explanation to describe the contextual vulnerabilities that were found, and a list of recommended FDA-approved drugs and investigational therapies in our study drug pharmacopeia relevant to the aberrations. Aberrations are classified according to the following criteria:

1.) <u>Level 1:</u> Published literature links a particular drug to the molecular aberration
2.) <u>Level 2:</u> Available information (via TCGA, Sanger Cosmic, etc.) indicates a particular aberration in molecular profile is thought to be involved in cancer, but published literature does not link this aberration to a particular drug
3.) <u>Level 3:</u> All other molecular aberrations

Level 1 and Level 2 aberrations will be included in the Genomics-Enabled Medicine for Melanoma Report listed in alphabetical order and presented in an unbiased, non-prioritized fashion. The interactive Genomics-Enabled Medicine for Melanoma report allows the reviewers to quickly navigate to the underlying knowledge and evidence at multiple levels (for example: to attain more information on the specific drug, the drug's putative target(s), clinical trials assessing the predicted drug's efficacy, and other levels of supporting evidence). The report conveys the predicted efficacy of the drugs identified by each of the analytical methods and also highlights evidence that supports or refutes the use of the predicted drug in the context of the patient's disease state (e.g., BRAF wild type metastatic melanoma).

As a result of molecular profiling, there will be three possible scenarios:
1) The patient is found to have a BRAF V600 mutation in contrast to the prior negative results obtained via a CLIA certified assay. If this event occurs, the patient will be removed from the study;
2) The profiling results in no potential match with any drug available in our pharmacopeia. In this event, the patient will be removed from the study;
3) The data defines a potential match (or multiple matches) with a targeted therapy that exists in our pharmacopeia. In this scenario, the Genomics-Enabled Medicine for Melanoma report will be completed and distributed to the Molecular Tumor Board.

The drug-rule match algorithm is a mechanistic process operated on the GEMM database that takes, as inputs, genomic aberrations and their values and identifies the drug candidates from the drug rule match knowledge base described above. The output consists of records containing both the matched biomarkers and drugs with available information in the knowledge base. In particular, this construct does not restrict the application, and any specific phenotype can be used to match drugs to genomic aberrations in any general setting.
The genomic aberrations are matched to drug rules in the knowledge base using the following criteria:
- Match based on the type of genomic aberration of a biomarker with or without exact match of the biomarker value
- Match based on the type of genomic aberration of a modifier with or without exact match of the modifier value

10. <u>Multi-disciplinary Molecular and Clinical Tumor Boards will convene once all data have been collated to discuss candidate biomarkers that are therapeutically actionable and assess the weight of evidence supporting the associated therapy (see Appendix XII for additional information)</u>

Molecular Tumor Board
The Molecular Tumor Board will assess the molecular profile generated for the individual patient and analyze the weight of evidence in support of a proposed target/drug match. The Molecular Tumor Board will meet by conference call/WebEx

weekly (or as needed) to review the Genomics-Enabled Medicine for Melanoma reports. Molecular profiling data will be given to the Molecular Tumor Board members a minimum of 48 hours in advance of the meeting. The Molecular Tumor Board will consist of expert members of the team as follows:

- Available clinical PIs
- Genomics experts involved in sequencing and analysis, knowledge mining, and computational and systems biology
- Bioinformaticians
- Pharmacy representation
- Available molecular biologists
- Patient advocates

At least one Genomics expert/bioinformatician, one pharmacy representative, one patient advocate, and three clinical investigators will be needed for a proper quorum to hold a Molecular Tumor Board meeting. Each Molecular Tumor Board conference call will be recorded in its entirety. A summary of the meeting will be written and presented to the Medical Overseer.

Clinical Tumor Board

A final recommended treatment plan will be generated by the Clinical Tumor Board utilizing the information contained in the knowledge mining analysis, information contained in the translational Genomics-Enabled Medicine for Melanoma report, and relevant patient clinical characteristics (e.g., history & physical, prior treatment history, comorbidities, etc.). Specific treatment details will consist of a regimen chosen from a guided list of agents implicated in critical molecular signaling pathways and/or from signature-based predictions of drug efficacy summarized in the Genomics-Enabled Medicine for Melanoma report. All agents are listed in the current pharmacopoeia we are planning to use in this study, but the selected therapy may differ amongst individual patients depending on several factors, including results of their unique molecular profiling. Potential drug interactions between the molecularly guided agents and the subject’s routine medications and supplements will be considered by the Clinical Tumor Board, as well as patient’s clinical characteristics and prior treatment history. The decision making process, including prioritization of treatments, will be based on the following:

1.) Depth of knowledge for each individual agent and its response in tumors with similar molecular signatures including (but not limited to)peer-reviewed published *in vivo* data, *in vitro* data, and response profile differential;
2.) Safety considerations, including (but not limited to) review of the patient’s history & physical, prior treatments, concurrent medications, and potential drug/drug interactions;
3.) Expertise of team in evaluating each target

The Clinical Tumor Board will meet by conference call/WebEx bi-weekly or more frequently as needed following each Molecular Tumor Board. The Clinical Tumor Board will consist of expert members of the team as follows:

- At least three study clinical investigators.
- Additional clinical investigators, as available
- At least one patient advocates (non-voting)
- At least one pharmacy representative (non-voting)

The final treatment recommendation will be made through a majority vote of all clinical investigators in attendance at the Clinical Tumor Board meeting. If a majority vote in favor of a specific treatment is not reached, discussion will be made whether more information is needed, including the possible reconvening of the Molecular Tumor Board, or whether the patient should come off study. In the event of an even number of clinical investigators being present on the call, one will be excluded at random prior to the vote so that a tie will not occur.

Representative members of the Clinical Tumor Board also will participate in the Molecular Tumor Board and thus be familiar with the discussions that have occurred. Each Clinical Tumor Board conference call will be recorded in its entirety. A summary of the meeting will be written and presented to the Medical Overseer. If a particular therapy is recommended, a Treatment Plan will be generated and sent to the Medical Overseer for review.

11. <u>The treatment plan with accompanying molecular aberrations and associated evidence linking the molecular aberration to a specific therapeutic agent(s) will be provided to an Independent Medical Overseer for review.</u>
Following discussions by the Clinical Tumor Board, the final Treatment Plan will be prepared for review by the Medical Overseer. This Treatment plan will include (but will not be limited to) the following:
- Information regarding the drug selected
- Summarized patient information as was presented to the Clinical Tumor Board, including pertinent relevant clinical characteristics including history & physical, comorbidities, prior treatment history, etc.
- Summaries from Molecular and Clinical Tumor Board meetings

The final Clinical & Molecular Tumor Board Treatment Plan will be presented to the independent Medical Overseer, who will have the opportunity to reject the purposed regimen requiring reconvening of the Clinical Tumor Board. The independent Medical Overseer will be a board-certified medical oncologist with extensive experience in early clinical trials. He/she will review the Tumor Board treatment recommendations and supervise trial conduct. The Medical Overseer will ensure that the study protocol and procedures were followed in the acquisition and analysis of the patient samples, that the drug suggested by the Genomics-Enabled Medicine Inference Report and recommended by the Clinical Tumor Board is anticipated to be safe for the patient, and that dose and drug administration scheduling are appropriate. The final treatment regimen, decision of the Medical Overseer, and the summaries of the Molecular and Clinical Tumor Board meetings will be documented. If deemed acceptable, the lead clinical site will be notified.

12. <u>A Treatment Memo will be generated outlining the risks of the recommended therapy and will require the signature of the patient or their legal representative.</u>
The patient will be assigned a treatment according to the results of the tumor board discussion. The assigned treatment regimen will be discussed with patients and their families, and will include a list of known side effects and any additional clinical monitoring that may be required or recommended by the FDA, the pharmaceutical company, and/or the Clinical Tumor Board. All patients must sign a treatment-specific memo. The patient will be given the option to proceed with therapy. The patient may also choose not to proceed with the assigned course of therapy; if this occurs, he/she will come off study.

13. <u>Treatment will be initiated.</u>

The patient's treating physician will be notified of the results of the tumor board decision. All patients will sign a Treatment Memo. Patients will undergo treatment according to the results of the tumor board decision, using the appropriate dose and schedule defined by the Clinical Tumor Board and approved by the Medical Overseer. Typical for any patient preparing to undergo cancer treatment, during the five-week window when data is being generated and analyzed, pretreatment evaluation will occur (e.g., CT scans, blood work, etc). If the assigned therapy requires the patient to undergo additional testing specific to a particular drug (as defined in the drug package insert for commercially-available agents or the drug-specific appendices of this protocol for investigational agents), it will be performed prior to treatment. We will attempt to initiate treatment of patients within 7 business days following their signing of the Treatment Memo; anticipated delays should be discussed with the study Principal Investigator. We fully recognize several scenarios will exist in which a specific therapy is assigned but the patient will not be able to undergo that particular treatment, including (but not limited to): unique drug-specific inclusion/exclusion restrictions, refusal, physician discretion, co-morbidities, prior treatments, and unexpected events occurring during the five-week analysis period of their tumor. If a patient is unable to receive the assigned treatment due to issues related to eligibility, or refuses to receive the recommended treatment, the patient will come off study and be treated by their physician as he/she deems appropriate. Basic work-up testing that was done during the "window" period can then be used as baseline evaluation for the treating physician's treatment choice.

Should the patient come off study following treatment initiation, a second surgical or biopsy procedure will occur, contingent upon funding. Whenever possible, we intend to collect fresh tissue prospectively during routine dissection procedures or from leftover biopsy/surgical tissues. When this is not possible, the patient will be given a full description of the biopsy procedure and associated risks. Common sample identifiers will be provided by the Study Coordinator and will be used by all parties throughout the project to ensure sample, data and report alignment between participating organizations.

As with the initial biopsy, for the second biopsy procedure we will collect five to six 1-2 centimeter 16- or 18-gauge core needle specimens from accessible tumor. Three of the cores will be placed in externally-threaded cryovials and immediately flash frozen in liquid nitrogen and stored at -80°C until shipping on dry ice. These three cores will be shipped to Ashion a central CLIA-certified laboratory affiliated with TGen in Arizona for DNA and RNA extraction and correlative analyses or biobanking (see Appendix IV). The fourth core will be cryopreserved in Optimal Cutting Temperature (OCT) compound for proteomics (to be sent to George Mason University; see Appendix VIII). A fifth core will be formalin-fixed and paraffin-embedded at the clinical site and the block shipped to Ashion (see Appendix VI). Additional tissue, if any is available, will be flash frozen and banked at TGen.

In cases where fresh tissue is accessible by surgical biopsy, a minimum of 50 mg of tissue will be collected and partitioned to reflect the distribution of the core biopsies described above. Patient identifiers will remain under HIPAA compliance, and clinical information will be kept in accordance with each site's standard practice. Sample kits containing barcoded tubes for shipping samples to the appropriate laboratory will be provided.

Order of fresh tissue priority (**<u>second biopsy</u>**):

1. Flash frozen core for DNA analysis (to be sent to Ashion; see Appendix IV);
2. Flash frozen core for RNA analysis (to be sent to Ashion; see Appendix IV);
3. Cryopreservation of Tissue in OCT for proteomics (to be sent to George Mason University; see Appendix VIII)
4. Core for creation of formalin-fixed paraffin-embedded (FFPE) block (to be fixed/embedded at clinical site and sent to Ashion; see Appendix VI)
5. Flash frozen core for correlative analyses or biobanking (to be sent to Ashion; see Appendix IV)
6. Any extra tissue will be flash frozen and biobanked (to be sent to Ashion; see Appendix IV)

Each patient will also have blood collected at the time of the second biopsy as follows:

1.) All patients will have 10 mL of blood collected at the second biopsy timepoint (prior to the actual biopsy procedure) for preparation of plasma for analysis of circulating tumor nucleic acids (ctNA) at TGen (see Appendix VII)
2.) Patients who have progressed on treatment will have 5.5mL of blood collected at the second biopsy timepoint (prior to the actual biopsy procedure) for RNA gene expression profiling and assays at Merck (see Appendix IX).

Appendix X contains a table summarizing sample distribution from the clinical sites. Appendices IV, VI, VII, VIII, and IX contain detailed standard operating procedures for the sample procurement, handling, processing, and shipping procedures for tissue and blood collected at the time of second biopsy. Figure 5 depicts the planned specimen processing and workflow for blood and tissue collected at the time of the second biopsy.

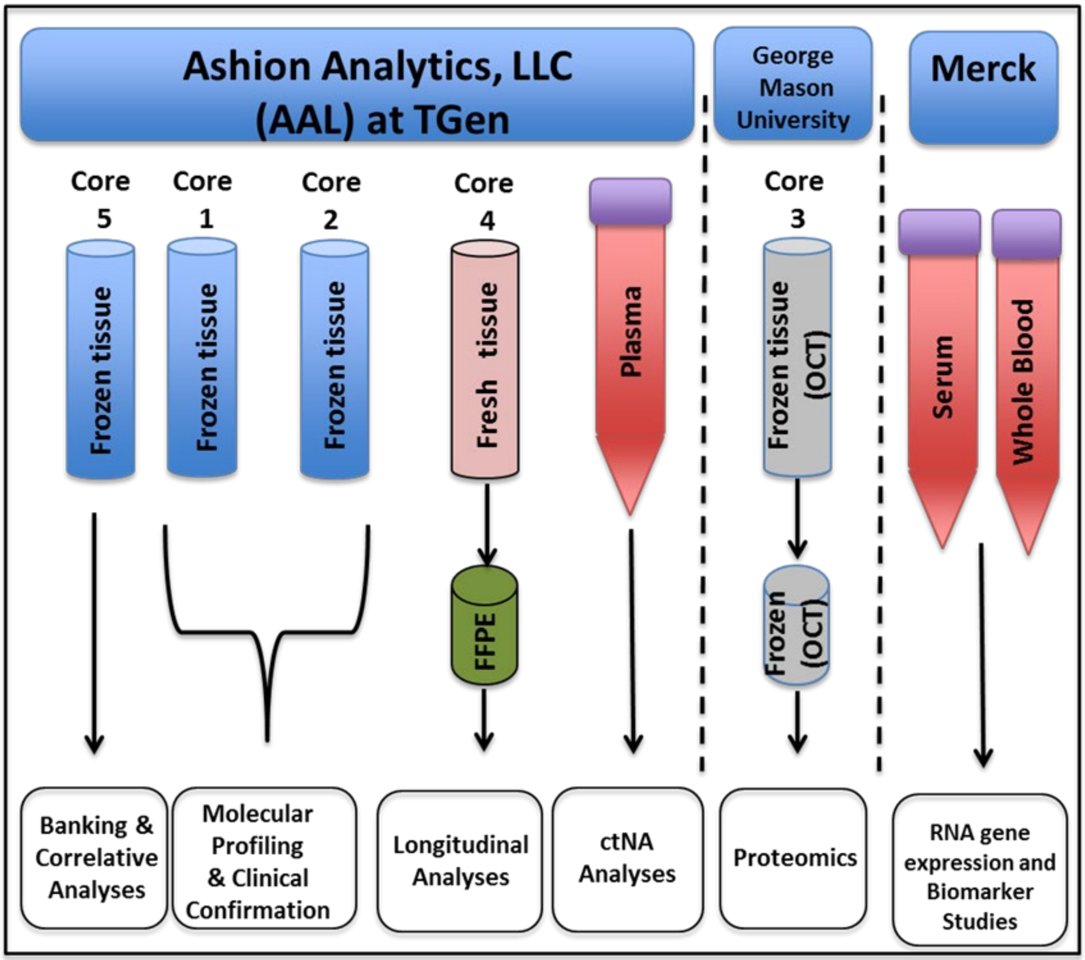

Figure 5: Specimen Processing and Workflow – Second biopsy

8.2 *General Concomitant Medication and Supportive Care Guidelines*

All intercurrent medical conditions will be treated at the discretion of the Investigator according to acceptable community standards of medical care. All concomitant medications and treatments will be documented on the appropriate case report form.

Specific concomitant medication and supportive care guidelines will be presented in the appropriate Appendix for each drug in the study pharmacopeia. Any concomitant medications will be discussed during the Tumor Board presentation.

In general, because there is a potential for interaction of many of the agents in the pharmacopeia with other concomitantly administered drugs through the cytochrome P450 system, the case report form must capture the concurrent use of all other drugs, over-the-counter medications, or alternative therapies.

The following medications are not permitted at any time while on study:

- Any cytotoxic chemotherapy
- Any other investigational treatment
- Any other systemic anti-neoplastic therapy including, but not limited to, immunotherapy, hormonal therapy, targeted therapies, anti-angiogenic therapies, or monoclonal antibody therapy

The following medications/treatments may be administered as follows:

- Any radiotherapy administered with palliative intent/pain control and not to an area that encompasses any target lesion being followed unless there are multiple targeted lesions being followed.
- Blood products, anti-emetics, steroids, and transfusions may be administered at the discretion of the Investigator based on established criteria. Consideration for use of hematologic growth factors may occur after Cycle 1, if necessary, as per current ASCO guidelines. However, colony stimulating factors should not be administered prophylactically prior to the first dose of study treatment.
- Complementary and alternative medicines (CAMs) are not permitted without prior approval from the study Principal Investigator
- Anti-emetogenic agents may be administered at the discretion of the Principal Investigator but are not commonly required as a prophylactic agent. Although not prohibited, the use of benzodiazepines for the prophylaxis or treatment of nausea or vomiting is discouraged. Medications with potential CNS effects are not prohibited in this study, but it is recommended that their use be minimized to avoid confusion in the interpretation of CNS effects should they occur during the course of treatment.
- Megace© is allowed for appetite stimulation.
- If patient is on prior bisphosphonates, continuation may be allowed at the discretion of the Principal Investigator.

8.3 *Duration of Study*

8.3.1 Prior to Treatment

Patients will be removed from study if any of the following occur prior to initiation of therapy:

- following biopsy/resection, if the tumor sample is found to be inadequate for comprehensive molecular analysis, a second biopsy may be performed after discussion and confirmation with the Principal Investigator. If the tumor sample is found to be inadequate for comprehensive molecular analysis, the patient will be deemed ineligible and will be replaced
- molecular profiling results in no drug available in our pharmacopeia. Patients who do not have a druggable target will not be evaluable for response and will be replaced; they will be offered SoC therapy, or any other available clinical trials for which they are eligible.
- If, following molecular profiling, the patient is found to have a BRAF V600 mutation in contrast to negative previous results obtained via a CLIA certified assay. Patients who are found to have a BRAF mutation following molecular profiling will not be evaluable for response and will be replaced; they will be offered SoC therapy, or any other available clinical trials for which they are eligible.
- General or specific changes in the patient's condition render the patient

unacceptable for further treatment in the judgment of the investigator

- Patient or guardian decides to withdraw consent and wish to no longer participate in the study;
- Patient chooses to undergo non-study assigned treatment for their cancer.

8.3.2 Following Initiation of Treatment

In the absence of treatment delays due to adverse event(s), treatment may continue until one of the following criteria applies:

- Disease progression;
- Intercurrent illness that prevents further administration of treatment or requires a prohibited concomitant treatment;
- Patient or guardian decides to withdraw consent to continue participation in the study;
- General or specific changes in the patient's condition render the patient unacceptable for further treatment in the judgment of the investigator;
- Death
- Female participant becomes pregnant
- Maximum allowed cumulative dose of drug, when known and appropriate
- Unacceptable adverse event(s), which in the opinion of the Investigator, precludes further trial participation or fulfills the protocol requirements for withdrawal;

At the time of disease progression/end of study, patients will be re-biopsied, contingent upon funding. Patients who progress on molecularly-guided treatment will be offered SoC therapy, or any other available clinical trials for which they are eligible, and will come off study.

8.4 *Duration of Follow-Up*

Adverse event data will be collected for subjects who receive at least one dose of treatment for 30 days following the last treatment, suspected study drug-related toxicity at the 30 day follow-up visit must continue to be followed until resolution to baseline or ≤ Grade 2 or stabilization of the event upon follow up. For response, patients will be followed to disease progression or until the patient comes off study when any of the criteria listed in Section 8.3 applies. While response data will be collected on all patients, only those assigned to MEK162 will be evaluable for the primary endpoint.

8.5 *Criteria for Removal from Study:*

Patients will be removed from study when any of the criteria listed in Section 8.3 applies. The reason for study removal and the date the patient was removed must be documented in the Case Report Form. In addition, patients found to be ineligible, who have inadequate tissue for molecular analysis, or who are found not to have a druggable target following molecular analysis will go off study and be replaced.

8.6 *Safety Evaluations*

Safety analysis will be conducted on all patients who have received at least one dose of therapy, and will include the frequency of all adverse events and laboratory abnormalities as well as frequency of dose interruptions, dose reductions, and

treatment discontinuation. Subjects who receive one total cycle of treatment (typically 21 or 28 days, depending on the specific treatment) will be considered as having completed the evaluation for safety. Additional treatment cycles may be delivered in a maintenance setting if there are no safety concerns, there is no disease progression, and/or there is an indication of clinical benefit. Maintenance monitoring will occur on Day 1 of every additional treatment cycle, unless indicated.

Any safety concern or new information that might affect either the safety or the ethical conduct of this trial will be immediately forwarded to the Overall Principal Investigator and Medical Overseer in written form. The Overall Principal Investigator will be responsible for informing the IRB and Data and Safety Monitoring Committee (DSMC). If trends in toxicities are noted or stopping rules are met, the Overall Principal Investigator will temporarily suspend enrollment while reviewing the episodes with the IRB and DSMC. Toxicity data must be submitted to the Coordinating Center via Oncore at the end of each cycle of therapy.

Additional monitoring may be required based on the assigned treatment regimen and as recommended by the FDA for specific drugs. Any additional testing will be outlined in the Clinical Tumor Board treatment plan and included in the treatment memo.

Medical Overseer: is a medical oncologist with extensive experience in early clinical trials. The Medical Overseer must approve the treatment plan prior to drug administration.

Dose Modifications: dose modifications will occur according to directions specified in the drug package insert (for commercially-available agents) or according to the directions specified in the drug-specific appendix (for investigational agents).

Institutional Oversight: The Yale Cancer Center DSMC and the Office of Quality Assurance and Training at Yale Center for Clinical Investigation will provide independent oversight of this trial.

Teleconference/WebEx: The Principal Investigator will meet with the clinical research team at frequent and regularly scheduled intervals to determine treatment modifications and treatment based toxicities. It is imperative that maximum communication occur among all sites including: study coordinators, nursing, data management, pharmacists, regulatory, sample procurement/processing, and investigators/clinicians. Reassessment of the frequency of the teleconference/WebEx meeting may occur following enrollment and treatment of the initial patients to ensure adequate response time.

9 Adverse Events: List and Reporting Requirements

9.1 *Definitions*

9.1.1 Adverse Event (AE)

An ***adverse event (AE)*** is any untoward medical occurrence in a patient or clinical investigation subject administered a pharmaceutical product and which does not necessarily have to have a causal relationship with this treatment. An adverse event can therefore be any unfavorable and unintended sign (including an abnormal laboratory finding, for example), symptom, or disease

temporally associated with the use of a medicinal product, whether or not considered related to the medicinal product.

An untoward medical event which occurs outside the period of follow-up as defined in the protocol will not be considered an adverse event unless related to study drug. Worsening of a medical condition for which the efficacy of the study drug is being evaluated will not be considered an adverse event.

9.1.2 Unexpected Adverse Event

An ***unexpected adverse event (UAE)*** is one for which the nature or severity of the event is not consistent with the applicable product information as outlined in the package insert filed with the FDA (for commercial agents) or the Investigator's Brochure (for investigational agents).

9.1.3 Serious Adverse Event (SAE)

An AE is considered a ***serious adverse event (SAE)*** if it results in ANY of the following outcomes:

- Death
- A life-threatening adverse event. An adverse event or suspected adverse reaction is considered "life-threatening" if, in the view of either the investigator or sponsor, its occurrence places the patient or subject at immediate risk of death. It does not include an adverse event or suspected adverse reaction that, had it occurred in a more severe form, might have caused death.
- An adverse event that results in inpatient hospitalization or prolongation of existing hospitalization for ≥ 24 hours
- A persistent or significant incapacity or substantial disruption of the ability to conduct normal life functions
- A congenital anomaly/birth defect.
- Important Medical Events (IME) that may not result in death, be life threatening, or require hospitalization may be considered serious when, based upon medical judgment, they may jeopardize the patient or subject and may require medical or surgical intervention to prevent one of the outcomes listed in this definition. (FDA, 21 CFR 312.32; ICH E2A and ICH E6).

The term "severe" is often used to describe the intensity (severity) of an event; the event itself may be of relatively minor medical significance (such as a severe headache). This is not the same as "serious", which is based on patient/event outcome or action usually associated with events that pose a threat to a patient's life or functioning.

9.1.4 Exposure During Pregnancy

Exposure during pregnancy relates to pregnancies where the fetus (from pre-embryo to birth) may have been exposed to study drug at any time during study

participation. This applies to female study participants and female partners of male study participants.

9.2 *Grading and Relatedness of Adverse Events*

9.2.1 Grading of Severity of an Adverse Event

Each adverse event will be graded for severity per the National Cancer Institute Common Terminology Criteria for Adverse Events (NCI CTCAE version 4.0), and these criteria must be used in grading the severity of adverse events. The criteria can be found at: http://ctep.cancer.gov/reporting/ctc.html

Grading of Severity of an Adverse Event Not Listed in Published Criteria:

For those adverse events which are not listed as part of the NCI CTCAE version 4.0, the same grading system should be used, where:

- **Mild** corresponds to an event not resulting in disability or incapacity and which resolves without intervention
- **Moderate** corresponds to an event not resulting in disability or incapacity but which requires intervention
- **Severe** corresponds to an event resulting in temporary disability or incapacity and which requires intervention
- **Life-threatening** corresponds to an event in which the patient was at risk of death at the time of the event
- **Fatal** corresponds to an event that results in the death of the patient

9.2.2 Relatedness to Study Drug

The Participating Site Investigator must attempt to determine if an adverse event is in some way related to the use of the study drug and define an attribution category. This relationship should be described as follows:

RELATIONSHIP	ATTRIBUTION	DESCRIPTION
Unrelated to investigational agent/intervention	Unrelated	The AE *is clearly **NOT** related* to the intervention.<br>The event is clearly due to causes distinct from the use of the study drug, such as a documented pre-existing condition, the effect of a concomitant medication, or a new condition which, based on the pathophysiology of the condition, and the pharmacology of the study drug, would be unrelated to the use of the study drug.
	Unlikely	The AE *is **doubtfully related*** to the intervention.<br>Adverse event does not have temporal relationship to intervention, could readily have been produced by the subject's clinical state, could have been due to environmental or other interventions, does not follow known pattern of response to intervention, does not reappear or worsen with reintroduction of intervention
	Possible	The AE ***may be related*** to the intervention.<br>The event follows a reasonable temporal sequence from administration of the study drug and the event follows a known response pattern to the study drug BUT the event could have been produced by an intercurrent medical condition which, based on the pathophysiology of the condition, and the pharmacology of the study

Related to investigational agent/intervention		drug, would be unlikely related to the use of the study drug OR the event could be the effect of a concomitant medication
	Probable	The AE ***is likely related*** to the intervention.<br>The event follows a reasonable temporal sequence from administration of the study drug, the event follows a known response pattern to the study drug AND the event cannot have been reasonably explained by an intercurrent medical condition OR the event cannot be the effect of a concomitant medication.
	Definite	The AE ***is clearly related*** to the intervention.<br>The event follows a reasonable temporal sequence from administration of the study drug, the event follows a known response pattern to the study drug and based on the known pharmacology of the study drug, the event is clearly related to the effect of the study drug. The adverse event improves upon discontinuation of the study drug and reappears upon repeat expos

9.3 *Documenting Adverse Events*

The Participating Site Investigator should elicit information regarding the occurrence of adverse events through open-ended questioning of the patient, physical examination and review of laboratory results.

All adverse events, whether serious or not, will be described in the source documents and should be captured on the adverse event case report form. All new events, as well as those that worsen in intensity or frequency relative to baseline, which occur after administration of study drug through the period of protocol-specified follow-up, must be captured.

Information to be reported in the description of each adverse event includes:

- A medical diagnosis of the event (if a medical diagnosis cannot be determined, a description of each sign or symptom characterizing the event should be recorded)
- The date of onset of the event
- The date of resolution of the event and whether the event is serious or not
- Action taken; drug treatment required; non-drug treatment required; hospitalization or prolongation of hospitalization required; diagnostic procedure performed; patient discontinued from the study
- Outcome: complete recovery or return to baseline; unknown/lost to follow-up; adverse event persisting; patient died (notify the Coordinating Center immediately, and complete the Serious Adverse Event page and the Final Visit section of the case report form)

Adverse events, regardless of suspected cause, will be collected for 30 days following the last treatment, suspected study drug-related toxicity at the 30 day follow-up visit must continue to be followed until resolution to baseline or ≤ Grade 2 or stabilization of the event. All adverse events will be reviewed by the Participating Site Principal Investigator.

9.4 *Reporting of Adverse Events*

The following should be followed for all AEs:

- Identify the type of event using the NCI CTCAE v4.0. The CTCAE provides descriptive terminology and a grading scale for each adverse event listed.

- Grade the event using the NCI CTCAE v4.0 according to the guidelines in Section 9.2.1.
- Determine whether the AE is related to the study drug. Attribution categories are as follows: unrelated, unlikely, possible, probable, and definite. Definitions for each category can be found in Section 9.2.2.
- Determine the prior experience of the AE. Expected events are those that have been previously identified as resulting from administration of the drug, as defined in Section 9.1.2. An AE is considered "expected" when it is listed in the drug-specific Appendix.
- Determine if the AE is categorized as a serious adverse event, according to the criteria in Section 9.1.3.

Any ≥ grade 3 AE, (whether it meets the definition of serious or not according to the criteria in Section 9.1.3) which occur anytime after the subject has been consented up to 30 days after the last dose of treatment, that is determined to be **both** *unexpected* (i.e. are not listed in the relevant drug-specific appendices) and *definitely, probably, or possibly related* to study drug must be reported to the Coordinating Center by telephone, fax, or e-mail within 24 hours and the site's local IRB per institutional policy. If full information is not known, additional follow-up by the Participating Site Investigator will be required. Institutions that have been granted access should enter the SAE into the OnCore® system within 24 hours of discovering the event, in addition to notifying the Coordinating Center.

All SAEs need to be reported to the Coordinating Center by as soon as possible, and no later than 24 hours from the time of event (or the study team becoming aware of the event). SAEs will be sent to the Coordinating Center via email to SAEreportingSU2C@yale.edu, using the SAE form provided by the Coordinating Center. In order to adhere to the 24 hour deadline, sites should complete and return the form with all available details. It is acceptable for some of the information to be incomplete if it is not yet known. All additional information learned after the first 24 hours will sent on the same form in a subsequent follow-up report to be filed as soon as information is available. Multiple follow-up reports are acceptable as new information is gathered.

All deaths within 30 days of the last study intervention must be reported to the Coordinating Center by telephone, fax, or e-mail within 24 hours. If full information is not known, additional follow-up by the Participating Site Investigator will be required.

All serious adverse events (according to the criteria in Section 9.1.3) that are unexpected (i.e. are not listed in the relevant drug-specific appendices) which occur anytime after the subject has been consented up to 30 days after the last dose of treatment, and are possibly probably, or definitely related to the research must be reported by the Coordinating Center to the FDA (via MedWatch report) according to FDA 21CFR312.32 within 15 calendar days of the event occurring. The coordinating site must also notify all participating investigators within the same 15 calendar day time frame. Expedited safety reports will be aggregated/batched and submitted to the FDA on a quarterly basis to the IND (because all drugs/combinations will already have a recommended phase 2 dose and prior safety experience).

For patients assigned to receive investigational agents or commercial agents provided by a pharmaceutical company, pharmaceutical company-specific reporting will be required according to additional instructions outlined in the drug appendices.

AE reporting must also be carried out by each participating site investigator, according to their local policy and procedures, to the Institutional Review Board (IRB) responsible for oversight of their patients. A copy of the local IRB's acknowledgement should be forwarded to the Coordinating Site Project Manager at Yale. All serious adverse events must be followed until resolution or stabilization. Unanticipated problems involving risk to subjects or others, serious adverse events related to participation in the study, and all volunteer deaths related to participation in the study should be promptly reported to the Coordinating Center.

9.4.1 Exposure During Pregnancy

Pregnancy, although not itself an SAE, must also be reported on a Medwatch form and be followed up to determine outcome, including spontaneous or voluntary termination, details of birth, and the presence or absence of any birth defects or congenital abnormalities. Exposure during pregnancy reports relate to pregnancies where the fetus (from pre-embryo to birth) may have been exposed to study drug at any time during study participation.
All exposure during pregnancy information is submitted to the Coordinating Center on a Medwatch form, irrespective of whether an AE has occurred, within 24 hours of awareness of the pregnancy and/or exposure.

10 Schedule of Assessments and Procedures

10.1 *Pre-study assessments and procedures*

The site investigator is responsible for keeping a record of all patients screened for entry into the study and subsequently excluded along with reasons for their exclusion.

A signed Patient Informed Consent Form must be obtained **prior to registration**. Unless otherwise noted, an attempt should be made to have the following screening procedures performed within 7 business days following signing of the consent form unless approval for delay has been provided by CSPM or designee via email confirmation:

1. It has been confirmed that the patient meets **all** inclusion criteria and **none** of the exclusion criteria **prior to registration**.
2. A medical history, including documentation of the evidence of BRAFwt metastatic melanoma as determined by a CLIA certified assay. Include all other pertinent medical conditions and a history of all prior medical treatments;
3. Concomitant medications/therapies including documentation of steroid use and dose;
4. Demographics, height, weight, and vital signs, including temperature, pulse rate, and blood pressure
5. Physical exam, including ECOG Performance status. See Appendix I
6. Serum chemistry (Albumin, alkaline phosphatase, total bilirubin, bicarbonate, BUN, calcium, chloride, creatinine, glucose, LDH, magnesium, phosphorus, potassium, total protein, SGOT [AST], SGPT [ALT], sodium), PT/PTT, and CBC w/diff, platelets for baseline levels and eligibility evaluation

7. Baseline EKG
8. Baseline ECHO or MUGA scan
9. Serum pregnancy test for female subjects of child bearing potential;
10. Availability of adequate tumor sample (either archival paraffin tissue block or 10 unstained slides of a primary or metastatic cancer lesion – initial metastasis preferred. Archival tissue collected prior to immunotherapy whenever possible) should be confirmed prior to enrollment. Archival samples should be shipped within 1 month after enrollment (see Appendix VI).

10.2 *Following verification of eligibility*

Following verification of eligibility, an attempt must be made to have the following performed within 7 business days of initial consent unless approval for delay has been provided by CSPM or designee via email confirmation:

1. A whole blood sample (10mL minimum, 20 mL preferred) will be drawn prior to biopsy/surgery and collected in purple top EDTA tubes for extraction of constitutional analytes and analysis of germline DNA. The sequencing of germline DNA will determine normal polymorphisms. See Appendix III for blood collection and shipping instructions.
2. All patients will have 10 mL of blood collected at the initial biopsy timepoint (prior to the actual biopsy procedure) for preparation of plasma for analysis of circulating tumor nucleic acids (ctNA) at TGen (see Appendix VII)
3. All patients will have 5.5 mL of blood collected at the initial biopsy (prior to actual biopsy procedure for RNA gene expression profiling and assays at Merck (see Appendix IX).
4. Solid tumor biopsy or surgical resection consisting of five to six 1-2 centimeter 16- or 18-gauge core needle specimens from accessible tumor or surgical specimen of equivalent or greater size. Tissue samples will be handled and shipped per Appendices IV, V, VI, and VIII.
5. Baseline adverse event assessment will be performed
6. Baseline Quality of Life assessment will be measured and collected for 20 pateints who receive at least one dose of study treatment (any time after consent but before first treatment)
7. CT or MRI of measurable disease sites. Scans must be performed ≤ 4 weeks prior to the initiation of study treatment.

10.3 *Following molecular profiling and tumor board/medical overseer evaluation:*

Following results of molecular profiling, and tumor board/medical overseer evaluation:

1. Concomitant medications/therapies including documentation of steroid use and dose;
2. Weight and vital signs, including temperature, pulse rate, and blood pressure
3. Physical exam, including ECOG Performance status. See Appendix I
4. Serum chemistry, PT/PTT, and CBC w/diff, platelets
5. Agent-specific screening/eligibility, depending on which drug has been matched to patient following results of genetic profiling and treatment assignment. (Refer to agent specific appendix)
6. Monitoring of AEs and review of concurrent illnesses

7. Begin treatment using agent-specific schedule and dosing depending on which drug has been matched to patient following results of genetic profiling and treatment assignment. Patients who receive oral study therapy will be given a toxicity/drug accountability diary.
8. All patients will also have 10 mL of blood collected on Cycle 1, Day 1 (prior to treatment) and Cycle 1, Day 8 or 15 (depending on which date their scheduled visit will occur, prior to treatment) for preparation of plasma for analysis of circulating tumor nucleic acids (ctNA) at TGen (see Appendix VII)
9. All patients will have 5.5 mL of blood collected on Cycle 1 Day 15 or Cycle 2 Day 1 (depending on cycle length, prior to treatment); and at the time of clinical response (if any) for RNA gene expression profiling and assays at Merck (see Appendix IX).
10. Serum pregnancy test for female subjects of child bearing potential;
11. Quality of Life assessment will be measured and collected for 20 pateints who receive at least one dose of study treatment, The second assessment will be done before treatment, following tumor board assignment (approximately 35 days following bisopsy)

10.4 *Additional treatment cycles (regular maintenance monitoring):*

***NOTE: Clinic visits on Day 8 or Day 15 of Cycle 1 is predicated on the total length of each cycle. For drugs with 28-day cycles, the clinic visit will occur on Day 15. For drugs with 21-day cycles, the clinic visit will occur on Day 8. After Cycle 1, patients will come in only on Day 1 of each cycle unless specified in the drug-specific appendix.**

1. Continue agent-specific schedule and dosing depending on which drug has been matched to patient following results of genetic profiling and tumor board assignment. Unless otherwise noted, beyond Cycle 1, a treatment window of +/- 2 days for subsequent cycles will be allowed.
2. Continue agent-specific safety evaluations depending on which drug has been matched to patient following results of genetic profiling and tumor board assignment.
3. Tumor and radiologic evaluation: extent of disease will be initially measured at 30-35 days, and at 8 or 9 week intervals (depending on length of assigned study drug treatment cycle; 9 weeks for 21 day cycle, 8 weeks for 28 day cycles) following the initial assessment thereafter.
4. Monitoring of AEs and review of concurrent illnesses on Day 1 of each cycle and Day 8 or 15* of Cycle 1 or as indicated
5. Review and recording of concomitant medications on Day 1 of each cycle and Day 8 or 15* of Cycle 1 or as indicated
6. Vital signs, including weight, temperature, pulse rate, and blood pressure on Day 1 of each cycle and Day 8 or 15* of Cycle 1 or as indicated
7. Physical exam, including ECOG Performance status on Day 1 of each treatment cycle or as indicated. See Appendix I
8. Serum chemistry, CBC w/diff, and platelets on Day 1 of each cycle and Day 8 or 15* of Cycle 1 or as indicated
9. Additional imagining or studies may be done at any time if clinically indicated by symptoms, exam, or tumor markers.

10. All patients will also have 10 mL of blood collected on Day 1 of each Cycle (prior to treatment) for preparation of plasma for analysis of circulating tumor nucleic acids (ctNA) at TGen (see Appendix VII)
11. All patients will have 5.5 mL of blood collected at the time of clinical response (if any) for RNA gene expression profiling and assays at Merck (see Appendix IX).
12. Patients receiving oral study therapy will be given a toxicity and/or drug accountability diary at the beginning of each cycle and reviewed at each subsequent clinic visit in order to assess their compliance and determine the need for technique/treatment alterations
13. Quality of Life assessment will be measured and collected for 20 patients who receive at least one dose of study treatment. The third and final assessment will be done following 1 cycle of treatment (Cycle 2 Day 1 only)

10.5 ***Off treatment Visit:***

Subjects will return to the clinic within 14 days whenever possible after the last dose of treatment for the off treatment study visit. Last dose of treatment is defined as the last day that the subject receives their assigned treatment. This includes subjects that change treatment regimens from the tumor board recommendation, reduce doses, or change prescribing factors. The following evaluations will be conducted:

1. Monitoring of AEs and review of concurrent illnesses
2. Review and recording of concomitant medications
3. Vital signs, including weight, temperature, pulse rate, and blood pressure
4. Physical exam, including ECOG Performance status. See Appendix I
5. Serum chemistry, PT/PTT, CBC w/diff, and platelets
6. For those patients who have come off treatment, attempts will be made to obtain a solid tumor biopsy or surgical resection consisting of five to six 1-2 centimeter 16- or 18-gauge core needle specimens from accessible tumor or surgical specimen of equivalent or greater size. This is contingent on funding. Samples will be handled and shipped per Appendicies IV, V, VI, and VIII.
7. All patients will have 10 mL of blood collected at the second biopsy timepoint (prior to actual biopsy procedure) for preparation of plasma for analysis of circulating tumor nucleic acids (ctNA) at TGen (see Appendix VII)
8. Patients who have progressed on treatment will have 5.5mL of blood collected at the second biopsy timepoint (prior to the actual biopsy procedure) for RNA gene expression profiling and assays at Merck (see Appendix IX).

10.6 ***30 Day Follow Up (End of Study) Visit***

Subjects will return to the clinic or study staff will call the patient approximately 30 (+5) days after the last dose of treatment for the end of study assessements. Last dose of treatment is defined as the last day that the subject receives their assigned treatment. This includes subjects that change treatment regimens from the tumor board recommendation, reduce doses, or change prescribing factors. The following evaluations will be conducted:

1. Monitoring of AEs and review of concurrent illnesses
2. Review and recording of concomitant medications

Any subject with a suspected study drug-related toxicity at the follow-up visit must be followed either by patient visits or phone calls until the drug related toxicities have resolved to ≤ Grade 2 or stabilization of the event. This may require additional clinical assessments and laboratory tests.

10.7 *Study Calendar (Pre-treatment)*

**Procedure**	**Screening**	**Following Registration**	**Stop here and follow calendar in assigned drug specific appendix for appropriate drug calendar[9]**
Sign Informed consent	X		
Confirm BRAF wt status[5]	X		
Demographics	X		
Medical history	X		
Concurrent meds	X		
Physical exam	X		
Vital signs	X		
Height	X		
Weight	X		
Performance Status	X		
CBC w/diff, plts	X		
PT/PTT	X		
Serum chemistry [1]	X		
Serum Pregnancy Test	X		
Tumor measurements and radiologic evaluations[6]	X[6]		
surgical resection &/or tumor biopsy[2]		X[2]	
Blood draw for Extraction of Constitutional Analytes [3]		X	
Blood draw for analysis of ctNA[10]		X[10]	
Archival tissue requested for research[4]	X		
Adverse events		X	
EKG	X		
MUGA or ECHO	X		
Blood draw for PAXgene and serum biomarkers[7]		X[7]	
Quality of Life Assessment (20 pts)[8]	X[8]		

1. Albumin, alkaline phosphatase, total bilirubin, bicarbonate, BUN, calcium, chloride, creatinine, glucose, LDH, magnesium, phosphorus, potassium, total protein, SGOT [AST], SGPT [ALT], sodium.
2. Pre-treatment biopsy: Five to six 1-2 centimeter 16- or 18-gauge core needle specimens from accessible tumor or surgical specimen of equivalent or greater size. Two of the cores will be placed in externally-threaded cryovials and immediately flash frozen in liquid nitrogen and stored at -80°C until shipping on dry ice. Both of these frozen cores will be shipped to Ashion a central CLIA-certified laboratory affiliated with TGen in Arizona for DNA and RNA extraction (see Appendix IV). Depending on need, a third core will either be flash frozen and sent to Ashion for correlative analyses or biobanking (see Appendix IV), or it will be placed immediately in RPMI media and shipped to Mayo Clinic in Arizona on frozen and refrigerated cool packs for implanting into immune compromised mice for primary tumorgraft development (see Appendix V). The fourth core will be cryopreserved in Optimal Cutting Temperature (OCT) compound for proteomics (to be sent to George Mason University; see Appendix VIII). A fifth core will be formalin-fixed and paraffin-embedded at the clinical site and the block shipped to Ashion (see Appendix VI). Archival (diagnosis) paraffin blocks or slides will also be shipped to Ashion (within 3 months of patient enrollment) for longitudinal analyses (see Appendix VI). Additional tissue, if any is available, will be flash frozen and banked at Ashion.
3. 10-20 mL of whole blood collected in purple top EDTA tubes at time of pre-treatment biopsy for extraction of constitutional analytes to be sent to Ashion (Appendix III). Samples are to be drawn prior to biopsy/surgery.
4. Availability of adequate tumor sample (either archival paraffin tissue block or 10 unstained slides of a primary or metastatic cancer lesion– initial metastasis preferred. Archival tissue collected prior to immunotherapy whenever possible) should be requested prior to enrollment. Samples should be shipped within 3 month after enrollment. (see Appendix VI)

5. Patient's melanoma must test negative for BRAF V600 mutations based on the results of a CLIA certified assay.
6. Initial screening scans should consist of patient's prior scans showing evidence of measureable disease. Pre-treatment scans must be performed within the following timeframe: ≤ 4 weeks prior to the start of therapy.
7. All patients will have 5.5 mL of blood collected on the day of initial biopsy (prior to actual biopsy procedure); Once on treatement, (see drug-specific appendices), patients will also have blood drawn on Cycle 1 Day 15 or Cycle 2 Day 1 depending on cycle length (Patients on 28 day cycle will have blood drawn on Cycle 1, Day 15 [prior to dosing], Patients on 21 day cycle will have blood drawn on Cycle 2, Day 1 [prior to dosing]; and at the time of clinical response and/or progression for RNA gene expression profiling and assays at Merck (see Appendix IX).
8. In this study, QoL will be measured and collected at three timepoints for 20 patients who receive at least one dose of study treatment: survey #1 at baseline (following initial consent); survey #2 before treatment, and survey #3 following 1 cycle of treatment.
9. Patients will follow the calendar in the assigned drug-specific appendix.
10. 10 mL of blood collected in purple top EDTA tube on the day of initial biopsy (prior to actual biopsy procedure); once on treatment, (see drug-specific appendices), patients will also have blood drawn on Cycle 1, Day 1 (prior to treatment); Cycle 1, Day 8 or 15 (depending on which date their scheduled visit will occur, prior to treatment); Day 1 of each subsequent cycle (prior to treatment), and day of second biopsy (end of study/progression, prior to actual biopsy procedure) for preparation of plasma for analysis of circulating tumor nucleic acids (ctNA) at TGen (see Appendix VII). Patients who cross-over to molecularly guided treatment will also have blood collected at the following timepoints: Cycle 1, Day 1 (prior to molecularly guided treatment); Cycle 1, Day 8 or 15 (depending on which date their scheduled visit will occur, prior to treatment); Day 1 of each subsequent cycle (prior to treatment), and at Off-Study visit for preparation of plasma for analysis of circulating tumor nucleic acids (ctNA) at TGen (see Appendix VII).

11 Protocol Drugs

Individual treatment will either consist of a regimen chosen from a molecularly guided list of agents.

11.1 *Drug Accountability*

Accountability for the drug at all study sites is the responsibility of the Principal Investigator. The Principal Investigator will ensure that the drug is used only in accordance with this protocol. Drug accountability records indicating the drug's delivery date to the site (if applicable), inventory at the site (if applicable), use by each patient, and return to a specific pharmaceutical company or disposal of the drug (if applicable, and as dictated by the specific pharmaceutical company) will be maintained by the clinical site. Accountability records will include dates, quantities, lot numbers, expiration dates (if applicable), and patient numbers.

11.2 *Molecularly Guided Therapy*

Drug assignment for this protocol is based upon the treatment plan decided by the Clinical Tumor Board. Patients may be assigned either commercially-available or investigational agents depending on their individual molecular signature. Agents available for use are listed in the study pharmacopoeia (Appendix II), but will differ amongst individual patients. Molecularly guided treatment regimens will be discussed with patients and will include review of known side effects per Pharmacopeia Library (Drug-Specific Appendix) (information obtained from package insert or Investigators' Brochures). Any additional clinical monitoring based on the specific treatment assigned will also be discussed with the patient prior to therapy initiation. Once a treatment has been assigned, the patient will be given the option to proceed with therapy and if he/she decides to proceed with the tumor board's treatment decision, they will be asked to sign a treatment specific memo.

11.2.1 Commercially available agents

Commercially available agents will be either be provided by the companies or ordered directly by the participating site. Investigators will follow all manufacturer (package insert), institutional, and Clinical Tumor Board treatment plan guidelines. If more than one dose or schedule are available for a particular agent, determination of a treatment plan will be made by the Clinical Tumor Board.

Should commercial agents be provided by the company, initial shipment of drug will be requested by the investigational pharmacy (IP) manager of the coordinating institution following approval of the protocol by the IRB and sent directly from the company to the participating site per treating hospital protocol. Subsequent shipments of drug will be ordered directly from the participating site to the company with the coordinating center notified.

11.2.2 Investigational agents

Molecularly guided investigational therapies will consist of specific agents that have known Recommended Phase 2 dosing (i.e. agents have already completed phase 1 testing).

Following IRB approval of the protocol, the initial shipment of IP will be requested by the IP manager of the coordinating institution and sent directly

from the company to the participating site per treating hospital protocol. Subsequent shipments of drug will be ordered directly from the participating site to the company with the coordinating center notified. Treatment will be administered per pharmaceutical company guidelines and tumor board recommendations.

12 Laboratory Evaluations

12.1 *DNA and RNA analysis (Ashion Analytics a central CLIA-certified laboratory affiliated with TGen)*

Isolation of DNA and RNA from patient samples will be performed for use in genomic studies. Nucleic acids will be extracted from tumor tissues (one 1-2 centimeter 16- or 18-gauge core needle specimen for RNA and a second for DNA analysis). Each patient will also have 10-20 mL of whole blood collected in purple top EDTA tubes for extraction of constitutional analytes. Appendices III and IV contain detailed procurement, handling, processing, and shipping details related to this work.

Analysis Deliverables: Deliverables will include analysis of tumor-specific mutations and differential expression of targetable genes and/or molecular pathways. For each melanoma patient we will compile a list of somatic alterations including: 1.) Somatic coding point mutations and frameshift mutations; 2.) Mutations in important domains or motifs (kinase, ligand binding, etc); 3.) Genes mapping within focal high level amplicons or homozygous deletions; 4.) Genes involved in translocations/fusions; 5.) Rank order of differentially expressed genes; and, 6.) Germline mutations or SNPs involved in high-risk cancer predisposition or drug metabolism. In addition, we will list the genes that become evident only from integrated analysis of genome and transcriptome analysis (e.g., a gene mapping to a large hemizygous deletion region containing an obviously inactivating frameshift or nonsense mutation in the retained allele). Lastly, a knowledge mining analysis and translational report will be generated for each patient's genetic alterations known to be relevant to pharmacological treatment pathways. Each report will provide an interpretation of the aberration, a mechanistic explanation to describe the contextual vulnerabilities that were found, and a ranking of putative therapeutic agents. The Tumor Board will evaluate this data and provide therapy recommendations.

12.2 *Analysis of circulating tumor nucleic acids (ctNA) at TGen*

Cell-free nucleic acid fragments contributed by a tumor into blood can be measured using molecular assays for cancer-specific somatic mutations. Circulating tumor nucleic acids (ctNA) can be an inherently cancer-specific biomarker for solid cancers such as melanoma, allowing non-invasive, repeatable sampling of the cancer genome while potentially overcoming spatial intra-tumor heterogeneity. Within the context of the SU2C trial, ctNA analysis can complement current strategies for:

1) molecular cancer stratification at presentation and at relapse
2) measurement of systemic tumor burden to assess initial tumor response and to monitory for recurrence/progression
3) assessment of sub-clonal tumor dynamics, treatment-driven cancer evolution and acquired resistance

A key strength of ctNA analysis is the ability to acquire longitudinal samples with minimal discomfort to a cancer patient. Blood samples will be collected at the following timepoints for each patient: day of initial biopsy (prior to actual biopsy procedure); Cycle

1, Day 1 (prior to treatment); Cycle 1, Day 8 or 15 (depending on which date their scheduled visit will occur, prior to treatment); Day 1 of each subsequent cycle (prior to treatment), and at the time of second biopsy (end of study/progression, prior to actual biopsy procedure) for preparation of plasma and buffy coat (peripheral blood cells) for analysis. For visits while the patient is on treatment, samples are best obtained prior to administration of drug on each visit. For visits while the patient is undergoing a biopsy, samples must be obtained prior to the biopsy procedure. Samples can be stored at -80°C and shipped in batches; see Appendix VII for detailed descritpions of the procedures for collection, handling, and shipping of these samples.

Aliquots of tumor DNA and RNA from initial and post-treatment biopsies (where available) will be analyzed to identify genomic alterations for comparison with plasma samples. Buffy coat samples will be analyzed to discriminate somatic genomic alterations from germline variants and for detection of tumor-related nucleic acids (from circulating tumor cells or cells of the immune system).

12.3 *Establishment of primary BRAFwt MM tumorgraft models*

To evaluate the performance of the individual predictive methodologies, a robust but relevant model system(s) is required. Each method can then be analyzed for its ability to predict drug response based on genomic profiling of the model. To this end, the Mayo Clinic in Arizona (Dr. Alex Sekulic) will oversee the establishment and characterization of primary patient tumorgrafts (so named to distinguish them from classical cell line xenografts that poorly reflect the patient's disease). These have been shown to closely resemble the histopathological and molecular characteristics of the originating patient's tumor (44).

Depending on availability and need (as determined by the principal investigator prior to biopsy), one fresh (unfrozen) 1-2 centimeter 16- or 18-gauge core needle specimen from each BRAFwt MM patient may be used for the establishment of tumorgraft models at Mayo Clinic (Arizona). Appendix V contains detailed procurement, handling, processing, and shipping details related to this work.

12.3.1 Tumor collection and establishment of melanoma tumorgrafts

Viable, fresh tissue (equivalent of one core biopsy sample) from tumor biopsy/surgical resection should be placed into RPMI transport media and shipped immediately overnight on frozen and refrigerated cool packs to Mayo Clinic (Arizona) (see Appendix V for detailed sample handling and shipping instructions). These will be used for implantation into immune compromised mice for primary patient tumorgraft development and the generation of primary cell lines. Tumor samples will be de-identified and labeled with subject's study number.

All tumorgraft development will be performed in the AAALAC-accredited viviarium at Mayo Clinic in Arizona with full IACUC approval under the direct supervision of Dr. Sekulic and a preclinical team experienced with a broad spectrum of preclinical animal models of human cancers, including melanoma. Standardized operating procedures (SOPs) developed to maximize probability of engraftment and establishment of this renewable tumorgraft resource will be used. Ten melanoma tumor explants have been created previously with this approach.

Depending on the amount of tissue provided, the cryopreserved melanoma issue fragments will be engrafted subcutaneously into 2 to 5, 6 week-old, gender matched immune compromised mice (NOG strain for initial transplant). All tumorgraft models that arise from this initial implant with primary patient material (conservatively estimated at 50% success rate) will be transplanted fresh into a second cohort of 5 mice, to ascertain tumorgraft viability through multiple generations. In addition, tumorgraft fragments from all generations will be used to establish cell lines from the primary patient tumor tissue. Tumorgraft fragments from both first and second generation tumorgrafts from each successful model will be cryopreserved in 10% DMSO/ 90% RPMI media and maintained in liquid nitrogen storage at Mayo Clinic. Cryopreserved fragments from each tumorgraft model that are successfully established in mice will also be evaluated for viability following cryopreservation. Viability of cryopreserved tumorgraft fragments assures that this model is a renewable resource that can be reconstituted when necessary for further study. This resource of viable cryopreserved primary patient melanoma tumorgraft models and corresponding cell lines will be made widely available to the melanoma community, in conjunction with de-identified clinical data relating to the patients history, treatments and outcomes.

The fresh (**NON FROZEN**) tumor fragments will be shipped overnight on frozen and refrigerated cool packs in transport media to the Mayo Clinic (Arizona). **Please see Appendix V for specific shipping instructions.**

12.4 ***Reverse Phase Protein Microarray***

Viable, fresh tissue (equivalent of one core biopsy sample) from tumor biopsy/surgical resection should be placed into Optimal Cutting Temperature (OCT) compound and shipped on dry ice to George Mason University (samples can be stored at -80ºC and shipped in batches; see Appendix VIII for detailed sample handling and shipping instructions). In this study, we will incorporate Reverse Phase Protein Microarray (RPMA) to measure the activity and level of FDA approved and experimental targeted therapy agents. The RPMA technology has been developed to address the analytical challenges of the sandwich and forward phase protein arrays (e.g. mismatch of sandwich antibody affinity, imprecision within and between analytes, and poor sensitivity). The platform has been designed to enable non-subjective, quantitative, multiplexed analysis of specific forms of cellular proteins (e.g. phosphorylated, unphosphorylated, and cleaved) from a limited amount of starting sample, such as with a fine needle aspirate or laser capture microdissected (LCM) cellular material from a needle biopsy specimen to procure pure populations of the target cells of interest. Particularly suited for clinical tissue samples, RPMA uses a single antibody directed against the epitope of interest.

A key attribute of the RPMA is the ability to quantitatively measure the activation state of hundreds of signaling proteins, often the drug targets themselves, concomitantly from only a few thousand cells, thus providing a critical means of broad-scale cell signaling analysis directly from tissue samples, cell culture models, and animal tissues from pre-clinical studies. The RPMA technology, invented in Dr Petricoin/Liotta's laboratory and now optimized for routine clinical sample analysis, is currently being employed within the CAP/CLIA complaint proteomics laboratory within the Center for Applied Proteomics and Molecular Medicine at George Mason University.

The RPMA format immobilizes an individual test sample in each array spot. An array can be comprised of up to hundreds of patient samples or cellular lysates. Each array

is incubated with a single primary antibody and a single analyte is measured. Since RPMAs maintain the concentration of the input sample, the sensitivity is greater as compared with a forward phase, (e.g. antibody array) probed with the same small number of input cells.

With the RPMA technology, a lysate from the laser capture microdissected tumor epithelial cells is printed along with high and low controls, and a calibration curve lysate series on a nitrocellulose coated slide. Each printed spot contains an immobilized analyte zone measuring only a few hundred microns in diameter. The detection probe can be tagged and signal amplified independently from the immobilized analyte protein. Coupling the detection antibody with highly sensitive amplification systems can yield detection sensitivities to fewer than 1,000 to 5,000 molecules per spot with good linearity (correlation coefficient or R^2 = 0.990-0.999) and inter-experiment precision (R^2= 0.973). Within run analytical precision is between a 3-13% CV (coefficient of variation).

The RPMA technology has been developed and optimized for performance as a fluorescent-based calibrated assay, generally identical in design and analysis to standard ELISA or standard clinical immunoassays. As a calibrated assay, each assay consists of:

a. Experimental patient samples printed in triplicate
b. High, and low controls built in
c. Calibrators, consisting of a 7-14-point curve whereby the analyte of interest is decreasing in concentration in the background of a constant protein concentration.

The analyte concentration is thereby determined by interpolation/extrapolation to a linear curve fit of the calibration curve and reported in relative fluorescent units.

12.5 *T Cell*

If there is sufficient material from the collected blood and tumor tissues, we will pursue additional sequencing of T cell receptors for the analysis T cell clonal responses. If available, up to 1 microgram of DNA extracted from each tumor and blood would be used for this analysis. The DNA will be sent to Adaptive Biotechnologies to perform the molecular profiling. The analysis of the obtained data would be performed in parallel at both Adaptive Biotechnologies and TGen.

The study may also include analysis of the archived FFPE specimens, collected prior to the enrollment into this study. Such analyses would be performed to allow a comparison with the molecular characterization performed on the specimens collected in conjunctions with this study. As such, the previously collected FFPE tissues would be retrieved from corresponding facilities, pending the availability, and molecularly analyzed using DNA and RNA sequencing and protein assessments.

12.6 *Quality of Life.*

The Functional Assessment of Chronic Illness Therapy is a widely-used set of self-report quality of life (QoL) measures that all share a common core set of items. These items assess patient well-being in the physical, functional, emotional, and social/family domains (45). For patients with cancer, the common core items representing the physical, functional, social/family, and emotional domains are collectively known as the Functional Assessment of Cancer Therapy – General (FACT-G). These common core items facilitate comparisons among patients with different types of malignancies. The core items are supplemented with disease-specific modules that contain items

addressing concerns most relevant to disease-specific subpopulations. A melanoma-specific module has been developed to accompany the FACT-G to provide greater sensitivity and discriminatory power for patients with all stages of melanoma (46). When the melanoma-specific items are combined with the FACT-G, the expanded questionnaire is known as the FACT-Melanoma (FACT-M). Studies have established the FACT-M as a valid and reliable measure of patient-reported health-related quality of life (47). In this study, QoL will be measured and collected at three timepoints for 20 patients who receive at least one dose of study treatment: survey #1 at baseline (following initial consent); survey #2 before treatment (approx 35 days following biopsy), and survey #3 following 1 cycle of treatment. Dr. Louis Penner, professor in the Population Studies and Disparities Research Program at Karmanos and the Department of Oncology, has an extensive background in psychometrics and the assessment of QoL and other self-report measures. He will perform exploratory analysis of patient responses on the FACT-M. Because of the small sample size of this pilot study, there will not be sufficient power to conduct meaningful statistical comparisons between groups. Within-group repeated measure analyses will have more power, but are still likely to be substantially underpowered. However, we can examine trends and carry out an exploratory pilot study of the feasibility of collecting QoL data within this context. This should enable us to define ways to maximize the reliability and validity of such data. Further, even with this very small heterogenous sample, we can conduct preliminary analyses of the psychometric properties of the FACT-M. These preliminary data will be used to guide how we can minimize attrition and collect reliable and valid QoL data in larger scale studies which contain enough statistical power to conduct meaningful comparisons between groups and within-group longitudinal analyses.

12.7 *Merck Sharp & Dohme (RNA gene expression profiling)*

The collection of tumor samples along with the baseline and post-dose blood samples, is for the purposes of RNA gene expression profiling. RNA gene expression profiling requires both tumor samples and blood samples for analysis. In addition serum will be collected for future biomarker studies.

13 Measurement of Effect

13.1 *Antitumor Effect – Solid Tumors*

For the purposes of this study, patients should be initially measured for response at 30-35 days following first treatment and re-evaluated for response every 9 or 8 weeks thereafter, depending on the cycle length of the assigned treatment (9 weeks for 21 day cycles, 8 weeks for 28 day cycles). In addition to a baseline scan, confirmatory scans should also be obtained 4 weeks following initial documentation of objective response.

Response and progression will be evaluated in this study using the new international criteria proposed by the revised Response Evaluation Criteria in Solid Tumors (RECIST) guideline (version 1.1) (48). Changes in the largest diameter (unidimensional measurement) of the tumor lesions and the shortest diameter in the case of malignant lymph nodes are used in the RECIST criteria.

13.1.1 Definitions

Evaluable for toxicity. All patients will be evaluable for toxicity from the time of their first treatment.

Evaluable for objective response. Only those patients who have measurable disease present at baseline, have received at least one cycle of therapy, and have had their disease re-evaluated will be considered evaluable for response. These patients will have their response classified according to the definitions stated below. (Note: Patients who exhibit objective disease progression prior to the end of cycle 1 will also be considered evaluable.)

Evaluable Non-Target Disease Response. Patients who have lesions present at baseline that are evaluable but do not meet the definitions of measurable disease, have received at least one cycle of therapy, and have had their disease re-evaluated will be considered evaluable for non-target disease. The response assessment is based on the presence, absence, or unequivocal progression of the lesions.

13.1.2 Disease Parameters

Measurable disease. Measurable lesions are defined as those that can be accurately measured in at least one dimension (longest diameter to be recorded) as ≥20 mm by chest x-ray or as ≥10 mm with CT scan, MRI, or calipers by clinical exam. All tumor measurements must be recorded in millimeters (or decimal fractions of centimeters).

Note: Tumor lesions that are situated in a previously irradiated area might or might not be considered measurable.

Malignant lymph nodes. To be considered pathologically enlarged and measurable, a lymph node must be ≥15 mm in short axis when assessed by CT scan (CT scan slice thickness recommended to be no greater than 5 mm). At baseline and in follow-up, only the short axis will be measured and followed.

Non-measurable disease. All other lesions (or sites of disease), including small lesions (longest diameter <10 mm or pathological lymph nodes with ≥10 to <15 mm short axis), are considered non-measurable disease. Bone lesions, leptomeningeal disease, ascites, pleural/pericardial effusions, lymphangitis cutis/pulmonitis, inflammatory breast disease, and abdominal masses (not followed by CT or MRI), are considered as non-measurable.

Note: Cystic lesions that meet the criteria for radiographically defined simple cysts should not be considered as malignant lesions (neither measurable nor non-measurable) since they are, by definition, simple cysts.

'Cystic lesions' thought to represent cystic metastases can be considered as measurable lesions, if they meet the definition of measurability described above. However, if non-cystic lesions are present in the same patient, these are preferred for selection as target lesions.

Target lesions. All measurable lesions up to a maximum of 2 lesions per organ and 5 lesions in total, representative of all involved organs, should be identified as **target lesions** and recorded and measured at baseline. Target lesions should be selected on the basis of their size (lesions with the longest diameter), be representative of all involved organs, but in addition should be those that lend themselves to reproducible repeated measurements. It may be the case that, on occasion, the largest lesion does not lend itself to reproducible measurement in which circumstance the next largest lesion which can be measured reproducibly should be selected. A sum of the diameters (longest

for non-nodal lesions, short axis for nodal lesions) for all target lesions will be calculated and reported as the baseline sum diameters. If lymph nodes are to be included in the sum, then only the short axis is added into the sum. The baseline sum diameters will be used as reference to further characterize any objective tumor regression in the measurable dimension of the disease.

Non-target lesions. All other lesions (or sites of disease) including any measurable lesions over and above the 5 target lesions should be identified as **non-target lesions** and should also be recorded at baseline. Measurements of these lesions are not required, but the presence, absence, or in rare cases unequivocal progression of each should be noted throughout follow-up.

13.1.3 Methods for Evaluation of Measurable Disease

All measurements should be taken and recorded in metric notation using a ruler or calipers. All baseline evaluations should be performed as closely as possible to the beginning of treatment and never more than 4 weeks before the beginning of the treatment.

The same method of assessment and the same technique should be used to characterize each identified and reported lesion at baseline and during follow-up. Imaging-based evaluation is preferred to evaluation by clinical examination unless the lesion(s) being followed cannot be imaged but are assessable by clinical exam.

Clinical lesions Clinical lesions will only be considered measurable when they are superficial (*e.g.*, skin nodules and palpable lymph nodes) and ≥10 mm diameter as assessed using calipers (*e.g.*, skin nodules). In the case of skin lesions, documentation by color photography, including a ruler to estimate the size of the lesion, is recommended.

Chest x-ray Lesions on chest x-ray are acceptable as measurable lesions when they are clearly defined and surrounded by aerated lung. However, CT is preferable.

Conventional CT and MRI This guideline has defined measurability of lesions on CT scan based on the assumption that CT slice thickness is 5 mm or less. If CT scans have slice thickness greater than 5 mm, the minimum size for a measurable lesion should be twice the slice thickness. MRI is also acceptable in certain situations (*e.g.* for body scans).

Use of MRI remains a complex issue. MRI has excellent contrast, spatial, and temporal resolution; however, there are many image acquisition variables involved in MRI, which greatly impact image quality, lesion conspicuity, and measurement. Furthermore, the availability of MRI is variable globally. As with CT, if an MRI is performed, the technical specifications of the scanning sequences used should be optimized for the evaluation of the type and site of disease. Furthermore, as with CT, the modality used at follow-up should be the same as was used at baseline and the lesions should be measured/assessed on the same pulse sequence. It is beyond the scope of the RECIST guidelines to prescribe specific MRI pulse sequence parameters for all scanners, body parts, and diseases. Ideally, the same type of scanner should be used and the image acquisition protocol should be followed as

closely as possible to prior scans. Body scans should be performed with breath-hold scanning techniques, if possible.

PET-CT At present, the low dose or attenuation correction CT portion of a combined PET-CT is not always of optimal diagnostic CT quality for use with RECIST measurements. However, if the site can document that the CT performed as part of a PET-CT is of identical diagnostic quality to a diagnostic CT (with IV and oral contrast), then the CT portion of the PET-CT can be used for RECIST measurements and can be used interchangeably with conventional CT in accurately measuring cancer lesions over time. Note, however, that the PET portion of the CT introduces additional data which may bias an investigator if it is not routinely or serially performed.

Ultrasound Ultrasound is not useful in assessment of lesion size and should not be used as a method of measurement. Ultrasound examinations cannot be reproduced in their entirety for independent review at a later date and, because they are operator dependent, it cannot be guaranteed that the same technique and measurements will be taken from one assessment to the next. If new lesions are identified by ultrasound in the course of the study, confirmation by CT or MRI is advised. If there is concern about radiation exposure at CT, MRI may be used instead of CT in selected instances.

Endoscopy, Laparoscopy The utilization of these techniques for objective tumor evaluation is not advised. However, such techniques may be useful to confirm complete pathological response when biopsies are obtained or to determine relapse in trials where recurrence following complete response (CR) or surgical resection is an endpoint.

Tumor markers Tumor markers alone cannot be used to assess response. If markers are initially above the upper normal limit, they must normalize for a patient to be considered in complete clinical response. Specific guidelines for both CA-125 response (in recurrent ovarian cancer) and PSA response (in recurrent prostate cancer) have been published [*JNCI* 96:487-488, 2004; *J Clin Oncol* 17, 3461-3467, 1999; *J Clin Oncol* 26:1148-1159, 2008]. In addition, the Gynecologic Cancer Intergroup has developed CA-125 progression criteria which are to be integrated with objective tumor assessment for use in first-line trials in ovarian cancer [*JNCI* 92:1534-1535, 2000].

Cytology, Histology These techniques can be used to differentiate between partial responses (PR) and complete responses (CR) in rare cases (*e.g.*, residual lesions in tumor types, such as germ cell tumors, where known residual benign tumors can remain).

The cytological confirmation of the neoplastic origin of any effusion that appears or worsens during treatment when the measurable tumor has met criteria for response or stable disease is mandatory to differentiate between response or stable disease (an effusion may be a side effect of the treatment) and progressive disease.

FDG-PET While FDG-PET response assessments need additional study, it is sometimes reasonable to incorporate the use of FDG-PET scanning to complement CT scanning in assessment of progression (particularly possible 'new' disease). New lesions on the basis of FDG-PET imaging can be identified according to the following algorithm:

a. Negative FDG-PET at baseline, with a positive FDG-PET at follow-up is a sign of PD based on a new lesion.

b. No FDG-PET at baseline and a positive FDG-PET at follow-up: If the positive FDG-PET at follow-up corresponds to a new site of disease confirmed by CT, this is PD. If the positive FDG-PET at follow-up is not confirmed as a new site of disease on CT, additional follow-up CT scans are needed to determine if there is truly progression occurring at that site (if so, the date of PD will be the date of the initial abnormal FDG-PET scan). If the positive FDG-PET at follow-up corresponds to a pre-existing site of disease on CT that is not progressing on the basis of the anatomic images, this is not PD.

c. FDG-PET may be used to upgrade a response to a CR in a manner similar to a biopsy in cases where a residual radiographic abnormality is thought to represent fibrosis or scarring. The use of FDG-PET in this circumstance should be prospectively described in the protocol and supported by disease-specific medical literature for the indication. However, it must be acknowledged that both approaches may lead to false positive CR due to limitations of FDG-PET and biopsy resolution/sensitivity.

Note: A ‘positive’ FDG-PET scan lesion means one which is FDG avid with an uptake greater than twice that of the surrounding tissue on the attenuation corrected image.

13.1.4 Response Criteria

13.1.4.1 Evaluation of Target Lesions

Complete Response (CR): Disappearance of all target lesions. Any pathological lymph nodes (whether target or non-target) must have reduction in short axis to <10 mm.

Partial Response (PR): At least a 30% decrease in the sum of the diameters of target lesions, taking as reference the baseline sum diameters.

Progressive Disease (PD): At least a 20% increase in the sum of the diameters of target lesions, taking as reference the smallest sum on study (this includes the baseline sum if that is the smallest on study). In addition to the relative increase of 20%, the sum must also demonstrate an absolute increase of at least 5 mm. (Note: the appearance of one or more new lesions is also considered progressions).

Stable Disease (SD): Neither sufficient shrinkage to qualify for PR nor sufficient increase to qualify for PD, taking as reference the smallest sum diameters while on study.

13.1.4.2 Evaluation of Non-Target Lesions

Complete Response (CR): Disappearance of all non-target lesions and normalization of tumor marker level. All lymph nodes must be non-pathological in size (<10 mm short axis).

Note: If tumor markers are initially above the upper normal limit, they must normalize for a patient to be considered in complete clinical response.

Non-CR/Non-PD: Persistence of one or more non-target lesion(s) and/or maintenance of tumor marker level above the normal limits.

Progressive Disease (PD): Appearance of one or more new lesions and/or *unequivocal progression* of existing non-target lesions. *Unequivocal progression* should not normally trump target lesion status. It must be representative of overall disease status change, not a single lesion increase.

Although a clear progression of "non-target" lesions only is exceptional, the opinion of the treating physician should prevail in such circumstances, and the progression status should be confirmed at a later time by the review panel (or Principal Investigator).

13.1.4.3 **Evaluation of Best Overall Response**

The best overall response is the best response recorded from the start of the treatment until disease progression/recurrence (taking as reference for progressive disease the smallest measurements recorded since the treatment started). The patient's best response assignment will depend on the achievement of both measurement and confirmation criteria.

For Patients with Measurable Disease (*i.e.*, Target Disease)

Target Lesions	Non-Target Lesions	New Lesions	Overall Response	Best Overall Response when Confirmation is Required*
CR	CR	No	CR	≥4 wks. Confirmation**
CR	Non-CR/Non-PD	No	PR	≥4 wks. Confirmation**
CR	Not evaluated	No	PR	
PR	Non-CR/Non-PD/not evaluated	No	PR	
SD	Non-CR/Non-PD/not evaluated	No	SD	Documented at least once ≥4 wks. from baseline**
PD	Any	Yes or No	PD	no prior SD, PR or CR
Any	PD***	Yes or No	PD	
Any	Any	Yes	PD	

- See RECIST 1.1 manuscript for further details on what is evidence of a new lesion.

** Only for non-randomized trials with response as primary endpoint.

*** In exceptional circumstances, unequivocal progression in non-target lesions may be accepted as disease progression.

Note: Patients with a global deterioration of health status requiring discontinuation of treatment without objective evidence of disease progression at that time should be reported as "*symptomatic deterioration.*" Every effort should be made to document the objective progression even after discontinuation of treatment.

For Patients with Non-Measurable Disease (*i.e.*, Non-Target Disease)

Non-Target Lesions	New Lesions	Overall Response
CR	No	CR
Non-CR/non-PD	No	Non-CR/non-PD*

Not all evaluated	No	not evaluated
Unequivocal PD	Yes or No	PD
Any	Yes	PD
• 'Non-CR/non-PD' is preferred over 'stable disease' for non-target disease since SD is increasingly used as an endpoint for assessment of efficacy in some trials so to assign this category when no lesions can be measured is not advised		

13.1.5 Duration of Response

Duration of overall response: The duration of overall response is measured from the time measurement criteria are met for CR or PR (whichever is first recorded) until the first date that recurrent or progressive disease is objectively documented (taking as reference for progressive disease the smallest measurements recorded since the treatment started).

The duration of overall CR is measured from the time measurement criteria are first met for CR until the first date that progressive disease is objectively documented.

Duration of stable disease: Stable disease is measured from the start of the treatment until the criteria for progression are met, taking as reference the smallest measurements recorded since the treatment started, including the baseline measurements.

13.1.6 Progression-Free Survival

Progression Free Survival (PFS) is defined as the duration of time from start of treatment to time of progression or death, whichever occurs first.

14 Statistics

All patients will be enrolled and receive molecularly guided treatment but a vast majority of those were be treated with MEK162. Based on the initial 23 patients enrolled on this study who underwent molecular profiling and tumor board evaluation, 20 were assigned MEK162. We will not randomize patients to physician's choice of care, as listed in the original protocol.

14.1 *Primary Analysis*

The primary endpoint is best overall response (BORR) to therapy. For overall response, patients will be followed to disease progression or until the patient comes off study when any of the criteria listed in Section 8.3 applies. Only patients assigned MEK162 will be included in the primary analysis.

The primary statistical hypothesis is to compare BORR in MEK162 patients to a historical control response rate using a Simon two-stage design. The historical response rate is under 10% as documented by others (4-8). We will use a historical response rate of 7% as our null hypothesis. A Simon two-stage optimal design will enroll 22 MEK162 patients in stage 1 and terminate early if one or fewer patients respond (1/22 = 4.5%) If there are 2 or more MEK162 patients responding in stage 1, then we will enroll an additional 25 in stage 2 for a total of 47 receiving MEK162. If 6 or more of the 47 total patients respond (6/47 = 12.8%) then we will reject the lower response rate of 7% in favor of a higher rate. The statistical significance level of this design is .1. As an example of power, if the response rate is 20% or higher then this design has power 90%. The expected sample size is 33.5.

14.2 ***Secondary Analyses***

We will examine overall survival and progression-free survival of all patients and separately for those treated with MEK162 following personalized molecularly guided assignment. We will examine response rate of all patients enrolled (including those not receiving MEK162) and compare these rates to the historical control. We did not power for these endpoints and no corrections will be made for the multiplicity of these statistical tests.

Genetic markers will be compared across those patients who respond and those who do not. We will also examine marker differences by treatment received. Markers will be compared longitudinally, in patients for whom data is available on multiple time points. We will use the Benjamini-Hochberg criteria to control for false discovery rate.

14.3 ***Accrual Rate and Trial Duration***

We anticipate that approximately 25 to 30% of patients consented will not be eligible for treatment due to:

1) Insufficient tissue for genomic evaluation, failure in meeting minimal requirements for percent tumor nuclei, or insufficient quality of molecular analyte (5%);
2) Patient does not have BRAFwt MM upon re-evaluation (3-5%);
3) Co-morbidity exclusions (5-10%);
4) Ineligible due to development of other co-morbidities, or worsening disease, during the 5-week “wait” period (5-10%);
5) No drug available which matches with the molecular profile (5-10%);
6) Patient refusal (3-5%);
7) Alternative treatment executed (3-5%).

More than one of these attrition factors may occur within the same patient. We assume approximately 60 patients with BRAFwt disease will have to be consented in order to have 47 patients treated. All participating clinical sites are experienced in tissue acquisition and clinical trial execution. Over 1000 new patients with advanced metastatic melanoma are collectively seen across these sites annually. BRAF V600 mutations are present in approximately 50% of all metastatic melanomas.

We have chosen sites that have very large melanoma populations and whose melanoma programs are one of the main programs of their clinical trial operation and cancer treatment. As example, Mayo Clinic Rochester (main campus) treats approximately 800 metastatic melanoma patients every year. Similarly, the goal of screening more than 1000 patients will not be difficult to achieve. Once the trial is running at all sites, we anticipate accrual will be approximately 10-12 patients/month.

We anticipate accrual to take approximately 18 months. There will be a start-up time for each of the sites after initial IRB approval of approximately three months so an additional five months has been added to this projected accrual time.

14.4 ***Follow-up Times***

Adverse event data will be collected for all subjects. Patients suspected of study drug-related toxicity at the 30 day follow-up visit will continue to be followed until resolution to baseline or ≤ Grade 2 or else stabilization of the event upon follow up. To assess overall response, each patient will be followed to disease progression or until the patient comes off study for any of the criteria listed in Section 8.3.

15 Tissue Donor Privacy and Confidentiality

We recognize that the process of both contribution and access to human specimens needs to be transparent and governed by sound ethical and scientific principles so we can assure our patients that their free donations are put to as appropriate a use as possible. We feel confident that the studies performed with these specimens are important, are covered by the individuals' initial consent, and pose no greater than minimal risk.

It is the responsibility of the coordinating site-Principal Investigator to ensure that confidentiality for all patients participating in the trial and all of their medical information is maintained. Case report forms and other documents submitted must never contain the name of a trial participant. Each patient in the trial will be identified by a unique identifier that will be used on all CRF's and any other material. All case report forms and any identifying information will be kept in a secure location with access limited to the study staff directly participating in the trial.

Personal medical information may be reviewed by representatives of the coordinating site (Yale University), of the IRB or of regulatory authorities in the course of monitoring the progress of the trial. Every reasonable effort will be made to maintain such information as confidential. Personal identifying information will only be released with the express written permission of the tissue/blood donor along with IRB approval.

The results of the study may be presented in reports, published in scientific journals or presented at medical meetings; however, patient names will never be used in any reports about the study.

The OnCore clinical research management system (Forte Research Systems) at Yale University will be used to hold all clinical data pertaining to this protocol. The foundation of the security and access controls in OnCore is the Model Based Access Control technology that has been proven over the last few years at numerous clinical research centers. This has been extended to provide a detailed representation of patient consent, access requests and institutional policies concerning Protected Health Information (PHI), intellectual property, and institutional approvals for specimen requests. Thus, the system supports robust security and access control mechanisms sufficient to comply with both regulatory requirements such as HIPAA as well as institutional policies.

TGen information security services are provided via a robust security infrastructure that includes redundant Cisco firewall, Intrusion Detection Systems, and other access monitoring and control devices. TGen provides advanced storage area networks that currently provide over 600 terabytes of capacity and are capable of scaling into the petabytes. TGen maintains an enterprise class data center facility that is monitored 24x7x365 and provides redundant power and fire suppression systems designed to maximize system availability and data protection. This infrastructure combined with our services provides a fully managed computing environment that is responsive to the needs of the Institute.

In Ashion Analytics, data will be managed in a configurable, secure LIMS developed to provide all required workflows in the collection, QA/QC, specimen annotation, storage, analysis and reporting of specimens. The LIMS enables the tracking of specimen derivatives and associated laboratory activities and annotations. Specimens are manipulated according to appropriate SOPs, and any deviations from the SOPs can be recorded. Samples and reagents will be qualified for use in the procedures on criteria such as current acceptance status or reagent expiration date. The LIMS manages the workflow of laboratories for processing specimens into derivatives (e.g., DNA and RNA), including quality control workflow and annotations. This includes capturing pathology QC, molecular data and final

report results for each specimen. The data collected throughout all the steps of specimen collection, processing, analysis, and reporting is made available to authorized personnel only via Ashion Analytics LIMS.

15.1 *Access to Biospecimens and Donor Information*

Only personnel authorized by the Principal Investigators will have direct access to the specimen storage facilities and samples, including records pertaining to the identity of participants. Authorized study personnel at Yale will keep a copy of the signed consent form and the key linking the unique identifiers to the patient PHI. The master list linking the patient PHI and unique study identifiers will be kept in the Yale OnCore computer system with electronic safeguards. The access is limited to the designated Yale study personel. Other than elements of dates that will possibly needed by the Clinical Tumor Board to identify potential appropriate treatments (i.e. dates of initial diagnosis or prior treatment dates), and the unique de-identified coded sample number, no PHI will not be shared with entities outside of Yale.

Standard Yale procedure is to train requested staff on OnCore's Biospecimen Module (BSM) role and assign that to them. The scope of the role is limited to the protocol, in this case only the Clinical Research Support Lab at Yale is using Oncore's Biospecimen Module.

At Ashion Analytics, only laboratory personnel will have access to de-identified patient specimens. Ashion Analytics patient specimens are stored in a card-key access laboratory that only Ashion Analytics personnel have access to. Additionally, de-identified specimens will be shared with TGen study staff for the research detailed in this protocol.

15.2 *Research with de-identified specimens*

All specimens will be de-identified and coded with two unique identifiers before being sent to TGen. One identifier will be assigned by the Coordinating Site Project Manager at Yale while the second unique identifier will be assigned by Ashion Analytics at TGen.

15.3 *Biospecimen Banking*

Derivatives generated from the processed cells and the tumor specimen reserved for banking will be stored in Ashion Analytics under CLIA chain of custody. Banked specimens may be used for further validation or, if the patient agrees, for future medical research. Ashion Analytics has storage procedures designed to ensure that the storage process maintains the molecular and cellular integrity of the specimen.

When specimens arrive at Ashion Analytics, they will be entered in the LIMS and assigned an appropriate storage location. Both of the specimen's unique identifiers will be entered into the system and recorded on the specimen chain of custody form. The Chain of Custody form and LIMS will track specimen usage through the life of the specimen in Ashion Analytics. If a specimen or aliquot of derivatives is shared with another project investigator, it will be recorded and tracked in the LIMS, which will maintain a record for reporting and audit purposes.

The specimen and any other derivatives may be stored for up to 20 years in Ashion Analytics to answer research scientific questions related to cancer and/or study drugs. The subject retains the right to have the sample material destroyed at any time by contacting the Principal Investigator at Yale. If a patient requests sample destruction, the Yale Principal Investigator will provide Ashion Analytics with the patient's unique

identifiers. Ashion Analytics will remove any remaining patient specimen from storage and destroy the sample following standard operating procedures.

If a patient wishes to withdraw from the study at any time, he or she can contact the Yale study Principal Investigator or research nurse. When the patient withdraws from the study, the patient's specimens or residual specimens will be removed from storage and destroyed. Confirmation of destruction of the specimens will be forwarded to the Yale Principal Investigator, who in turn, will inform any withdrawing participants of the donated specimens that their samples have been destroyed. Withdrawing from the study will have no impact on the subject's medical care.

15.4 *Future Use of Banked Samples:*

Patients will have the option during the consent process of allowing their specimens to be used for additional medical research beyond the scope of this study, provided the specimens are completely de-identified. Use of this material will require prior review and approval by the IRB or record at the recipient investigator's site and approval from study Principal Investigators. Other than the de-identified sample number, no PHI will remain with the biobank-stored specimen.

15.5 *Restrictions on Sample Usage*

The intended use for these samples is to facilitate research. All investigators receiving samples will be reminded of their ethical and regulatory responsibilities concerning the use of such samples by providing them the following statement:

The recipient of any human samples will acknowledge that the conditions for use of this research material are governed by their site IRB in accordance with Department of Health and Human Services regulations noted in 45 CFR 46. The recipient agrees to comply fully with all such conditions and to report promptly to their IRB any proposed changes in the research project and any unanticipated problems involving risks to subjects or others. The recipient remains subject to applicable State or local laws or regulations and institutional policies that provide additional protections for human subjects. This research material may only be used in accordance with the conditions stipulated by the IRB.

15.6 *Incidental Findings*

During the course of data analysis, investigators may discover genetic information about the study participant that is not related to the current study (an incidental finding). We will follow the guidelines outlined by the American College of Medical Genetics and Genomics (ACMG) for incidental variants of known significance, when encountered (49). Investigators will consult with a genetic counselor if needed.

16 Study Data Handling and Record Keeping

Information about study subjects will be kept confidential and managed according to the requirements of the Health Insurance Portability and Accountability Act of 1996 (HIPAA). Those regulations require a signed subject authorization informing the subject of the following:

- What protected health information (PHI) will be collected from subjects in this study
- Who will have access to that information and why
- Who will use or disclose that information
- The rights of a research subject to revoke their authorization for use of their PHI.

In the event that a subject revokes authorization to collect or use PHI, the investigator, by regulation, retains the ability to use all information collected prior to the revocation of subject authorization. For subjects that have revoked authorization to collect or use PHI, attempts should be made to obtain permission to collect at least vital status (i.e. that the subject is alive) at the end of their scheduled study period.

The OnCore clinical research management system (Forte Research Systems) at Yale University will be used to hold all clinical data pertaining to this protocol. The Investigator will maintain records separate from the case report forms in the form of clinical charts, medical records, original laboratory, radiology and pathology reports, pharmacy records, etc. The Investigator will document in the clinic chart or medical record the name and number of the trial and the date on which the patient signed informed consent prior to the patient's participation in the trial. Source documents must completely reflect the nature and extent of the patient's medical care, and must be available for source document verification against entries in the case report forms when the monitor visits the investigational site. All information obtained from source documents will be kept in strict confidentiality.

All participating Investigators will retain the records of the study on site until the study is terminated and then records will be kept in storage for a minimum of 7 years. All study records must be maintained in a safe and secure location that allows for timely retrieval, if needed. Study records that must be retained include copies of case report forms, signed informed consents, correspondence with the IRB, source documents, clinic charts, medical records, laboratory results, radiographic reports and screening/enrollment logs. A copy of the signed consent form will be kept in the patient's medical record.

Should there be any changes in the archival arrangements for the study records, the coordinating site (Yale University) must be notified. The responsibility for maintaining the study records may be transferred to another suitable individual, but the coordinating site (Yale University) must be notified of the identity of the individual assuming responsibility for maintaining the study records and the location of their storage.

For purposes of capturing risks associated with the blood draw and biopsy procedures, the Common Terminology Criteria for Adverse Events (CTCAE) v4.0 will be used. Adverse events associated with these procedures will be collected in study case report forms.

16.1 *Deposition of Genotype Results to Public Databases*

The genotype results obtained during the course of the proposed study will be submitted to the **d**ata**b**ase of **G**enotypes **a**nd **P**henotypes (dbGaP), a database developed to archive and distribute the results of studies that have investigated the interaction of genotype and phenotype. Such studies include genome-wide association studies, medical sequencing, molecular diagnostic assays, as well as association between genotype and non-clinical traits. The advent of high-throughput, cost-effective methods for genotyping and sequencing has provided powerful tools that allow for the generation of the massive amount of genotypic data required to make these analyses possible.

dbGaP provides two levels of access - open and controlled - in order to allow broad release of non-sensitive data, while providing oversight and investigator accountability for sensitive data sets involving personal health information. Summaries of studies and the contents of measured variables as well as original study document text are generally available to the public, while access to individual-level data including phenotypic data tables and genotypes require varying levels of authorization.

All submissions to the NIH dbGaP of data will be accompanied by a certification by Institutional Official(s) at TGen that they approve submission to the NIH Genome-wide

association studies (GWAS) data repository. The certification will assure that:

- The proposed data submission is consistent with applicable federal and Virginia laws and regulations, as well as institutional policies;
- The research uses of the data and the uses that are specifically excluded by the informed consent documents are described.
- The proposal provides that the identities of research participants will not be disclosed to the NIH GWAS data repository; and
- The institution's Human Subjects Research Protections Program (HRPP)/IRB has reviewed the relevant aspects of the proposal and verified that
  - The proposed submission of data to the general NIH GWAS data repository for subsequent sharing for research purposes as described in the NIH Policy is not inconsistent with the informed consent of study participants from whom the data were obtained;
  - The investigator's plan for de-identifying datasets is consistent with the NIH "Policy for Sharing of Data Obtained in NIH Supported or Conducted Genome-Wide Association Studies"
  - Based on the characteristics of the subject population and the data involved in the primary study, and within the limits of its knowledge of the future potential uses and users of the data, it has considered the risks to individuals, their families, and groups or populations associated with the proposed submission of the data to the general NIH GWAS data repository. The IRB/Privacy Board understand that assessment of risks associated with specific future secondary uses will be performed by NIH's Data Access Committees, and,
  - To the extent applicable, the genotype and phenotype data proposed to be submitted were collected/will be collected in a manner consistent with 45 C.F.R. Part 46.

17 Plan for Communication of Collaborating Sites

Communication between centers will be critical in this trial. If a spot is available, the potential patient will undergo consent and completion of all required screening procedures and certification of all inclusion and exclusion criteria by the Investigator. If the patient fits all enrollment criteria, the study coordinator will notify TGen in preparation for collection and processing of tissue sample. The appropriate study staff will organize the convening of members of the tumor board for discussion of the current case once the full report is available. In addition a unique patient identifier will be assigned to the patient. The study coordinator will contact all sites via e-mail if the study enrollment is on hold or closed at any time.

Patient samples will be collected at each participating institution and sent to Ashion Analytics a central CLIA-certified laboratory affiliated with TGen as detailed above. TGen will perform molecular profiling of the tumor tissue and generate a report detailing the results. Once the report is generated by TGen, it will be sent to the Molecular and Clinical Tumor Boards. There will be a meeting of the tumor boards weekly or as needed to agree upon a treatment plan recommendation. The final recommendation from the tumor boards will be reviewed by the Medical Overseer.

18 Ethical Considerations

This study is to be conducted according to US and international standards of Good Clinical Practice (FDA Title 21 part 312 and International Conference on Harmonization guidelines) and the principles of the 2004 version of the Declaration of Helsinki. Applicable government

regulations and institutional research policies and procedures will also be followed. Each site will obtain IRB approval from their respective IRB's prior to collection and receipt of patient specimens.

19 Pre-Study Documentation

Each institution that wishes to participate in a Yale coordinated study is expected to comply with all applicable federal regulations and Yale multicenter trial requirements, the protocol and HIPAA requirements. Prior to activation of a study, the participating institution is expected to provide all required documentation requested in the start-up package. Upon receipt of all required site documents, the Coordinating Site Project Manager will issue a Site Activation Memo, which will open the participating institution to accrual. The following are **EXAMPLES** of documents that may be required.

- Participating Site Contact Information Sheet
- IRB Membership List
- Form Food & Drug Administration (FDA) 1572

  This document must be signed and dated by the Principal Investigator. In addition, all sub-investigators (if any) must be listed on the form. It is the Principal Investigator’s responsibility to assure that each sub-investigator is aware of their responsibilities as listed on the 1572. All other performance sites (including laboratories) must also be listed. The 1572 must be updated and forwarded to Yale as investigators and labs are added &/or deleted.
- The Investigator at each site must sign and date the Acknowledgement form (Appendix XI).
- The name and address listed on the 1572 form of the PI will be the address listed on the cover sheet of the protocol.
- Financial Disclosure form for each participating investigator
- Curriculum Vitae (CV)
  - A current copy is required for each PI
  - The front page must be signed and dated. A CV must be current within the year, and should be replaced prior to expiration.
  - The PI is responsible for maintaining a current CV on all subinvestigators listed on the 1572 form.
- A copy of medical license for each investigator
- A copy of Human Protection Training certificate for each investigator
- Laboratory Compliance Materials
  - Copies of the Clinical Laboratory Improvement Amendments (CLIA) accreditation and College of American Pathologists (CAP) certificates.
  - Copy of the normal ranges of laboratory values from each lab used with corresponding units.
  - These materials are required of every laboratory used in the conduct of this study. Each laboratory should be listed on the Form 1572.
  - New CLIA, CAP and lab normals are to be forwarded to Yale, so that the most current documents are kept on file.
- Informed Consent
  - A copy of the intended Informed Consent must be reviewed and approved by the Yale Regulatory Coordinator prior to IRB submission.
  - The consent must include the required elements as specified in the Code of Federal Regulations & Institutional Review Board (IRB) providing regulatory oversight:

        - A statement that the study involves research, and is conducted in a multi-institutional setting.
        - The purpose of the study.
        - Description of the procedures and/or treatment.
        - Procedures to be performed to monitor the patients.
        - Risks/discomforts.
        - Benefits, including monetary if applicable.
        - Alternatives
        - Confidentiality statement which includes oversight by state and federal authorities as well as the Yale Cancer Center designated study staff, IRB & their representatives.
        - Compensation for study related injury clause
        - Emergency treatment or injury will or will not be provided; other injury will or will not be compensated (i.e. mental injury, stress, etc.).
        - Contact person for questions.
        - Voluntary participation statement
        - Statement regarding how participation or lack of participation effects treatment & relation with physicians or institution.
        - Statement regarding how to withdraw from study and repercussions of withdrawal.
        - Statement regarding how new study information will be forwarded to participants
        - Cost statement
        - Signature line: participant; witness (if required); physician designee; all with date and time.
    - Each consent will be reviewed by the Yale Regulatory Coordinator to ensure that:
        - The IRB & Yale has permission to review all records involving the study and it is not held responsible for monetary compensation should study patient injury occur.
    - Yale CTO will make every effort to return and revise the contents of the consent within five working days of receipt.
- Review the HIPAA language for Yale & IRB
- Copy of the Institutional Review Board (IRB) approval, including the IRB approved consent
- Contract / Subcontract
  A contract or subcontract generated by Yale (or sponsor) must be reviewed and agreed upon. This contract must be signed and dated by both Yale administration and administration at the designated site, prior to site activation.
- Delegation of authority form

20 New Protocol Distribution

The coordinating center will distribute the final Yale Sponsor (Yale WIRB IRB approved) protocol and any subsequent amended protocols to all participating institutions.

The Yale Sponsor's informed consent template will serve as a template for the informed consent for participating institutions. The participating institution consent form must follow the consent template as closely as possible. Participating institutions are to send their version of the informed consent document and HIPAA authorization, if a separate document, to the Coordinating Site Project Manager for review and approval prior to

submission to their local IRB. The approved consent form must also be submitted to the coordinating center after approval by the local IRB for all consent versions.

21 Site initiation and Monitoring

The protocol will be open for accrual at the participating site after all required documents have been received including contract finalization.

Before the protocol is open for accrual, a user ID and password will be assigned and the site will be trained. The password will allow access to OnCore® for electronic data entry.

A monitor from the Coordinating Site will perform an initial site initiation visit, remote monitor and make scheduled trips as needed to each investigational site to review the progress of the trial. The actual frequency of monitoring trips will depend on the enrollment rate and performance at each site. The DSMC may request on-site monitoring. At each visit, the monitor will review various aspects of the trial including, but not limited to, screening and enrollment logs; compliance with the protocol and with the principles of Good Clinical Practice; completion of case report forms; source data verification; facilities and staff.

During scheduled monitoring visits, the Investigator and the investigational site staff must be available to meet with the study monitor in order to discuss the progress of the trial, make necessary corrections to case report form entries, respond to data clarification requests and respond to any other trial-related inquiries of the monitor.

In addition to the above, representatives of Stand Up To Cancer, Yale University Human Research Protection Program -Research Quality Assurance & Compliance or government inspectors may review the conduct/results of the trial at the investigational site. The Investigator at each site must promptly notify the Coordinating Center of any audit requests by regulatory authorities.

22 IRB Requirements

<u>New Protocol Submission:</u>

The participating site consent form will be reviewed to ensure its contents are both correct and complete and the format is unique to the participating site institution. The consent will also be reviewed to ensure that Yale University & the IRB has permission to review all records involving the study and it is not held responsible for monetary compensation should study patient injury occur. Expedited review of the initial submission for treatment protocols will not be accepted. The participating site institution will submit their IRB approval letter/form together with their approved consent & HIPAA form to the Yale Coordinating Center.

Once the regulatory requirements and contracts have been completed, and the IRB/consent approvals received by Yale, the Coordinating Site Project Manager will notify the participating site PI or a designee in writing that the trial is open for enrollment at the participating site.

<u>Annual Renewal Submission</u>

The participating site is required to submit an annual update to their IRB/ethics committee within 365 days from the initial approval. A copy of the IRB annual renewal approvals and consent changes are forwarded to the Coordinating Center on an ongoing basis.

Yale University will forward Data Safety Monitoring Reports and other pertinent communication to the participating site. It is the responsibility of the participating site to forward this information to their IRB for review. This information, at a minimum, should be included in the participating site's annual IRB submission.

Amendment, Revisions and SAE Distribution & Submission:
As protocol amendments are approved by the IRB, the Coordinating Center will forward the amended protocol and consent form (as applicable), to the participating site.

Upon receipt of the new version the participating site is responsible for the following:

- Confirm receipt of the amendment by e-mail or faxed letter at the time of receipt & forwarding a new signature page.
- If the amendment includes a consent change, modified participating site consent must be reviewed by the Coordinating Center for approval before it is sent to the local IRB.
- Updates are submitted to the local IRB as soon as possible after receipt. The amendment must receive IRB approval by the institution within three months (90 days) from the date that it was received.
- The Yale version date/and or amendment date should appear on the IRB approval letter/form. The date is located on the face page. The approval letter should reflect whether the protocol (only) has changed, or if the protocol &/or consent form have changes.
- A copy of the IRB approval (and amended consent if applicable) must be forwarded to the Coordinating Center within 30 days from time of approval.
- Changes to the consent may require participants to be re-consented if the change affects the study design, treatment or risk/benefit ratio. The Coordinating Center will notify sites if re-consenting is mandatory. The process for re-consenting is at the discretion of the local IRB unless specified by the sponsor. Re-consenting should be completed as soon as possible, but prior to, or during the next patient visit. Safety issues need to be communicated to the patient within a reasonable time frame, but no greater than 30 days.

The Coordinating Center at Yale will forward SAE's and IND Safety Reports to the participating site.

23 Data and Safety Monitoring

1. Protocol Research Team Meetings
   a. Scheduled meetings will be held monthly or more frequently depending on the activity of the protocol. These meetings will include the protocol investigators and research staff involved with the conduct of the protocol.

   b. During these meetings the investigators will discuss:

- Safety of protocol participants (adverse events and reporting)
- Validity and integrity of the data (data completeness on case report forms and complete source documentation)
- Enrollment rate relative to expectation of target accrual, (eligible and ineligible participants)
- Retention of participants, adherence to the protocol and protocol violations

2. Data and Safety Monitoring Committee
   a. The Yale Cancer Center Data and Safety Monitoring Committee (DSMC) will provide the primary oversight of data and safety monitoring. The Yale DSMC will review and

monitor compliance, toxicity and deviations from this study. The committee is composed of clinical specialists with experience in oncology and who have no direct relationship with the study. Information that raises any questions about participant safety will be addressed with the Overall PI, medical monitor and study team.

b. The DSMC will review this protocol monthly. Information to be provided to the committee includes: a study narrative by the PI, a summary DSMC report produced by OnCore (which includes participant accrual, response, trial status history, SAEs, Adverse Events, Deviations and survival); audit results, and monitoring reports as applicable. Other information (e.g. scans, laboratory values) will be provided upon request.

3. The YCCI Office of Quality Assurance and Training will audit the trial at least annually or as determined by the DSMC. The Overall Principal Investigator, study coordinator and/or data manager may request access to all source documents and other study documentation for on-site or remote monitoring, audit or inspection.

4. The Yale Human Research Protection Program, Research Quality Assurance & Compliance, may inspect study records during internal auditing procedures.

24 References:

1. Lovly CM, Dahlman KB, Fohn LE, Su Z, Dias-Santagata D, Hicks DJ, et al. Routine multiplex mutational profiling of melanomas enables enrollment in genotype-driven therapeutic trials. PLoS One. 2012;7:e35309.
2. Balch CM, Gershenwald JE, Soong SJ, Thompson JF, Atkins MB, Byrd DR, et al. Final version of 2009 AJCC melanoma staging and classification. J Clin Oncol. 2009;27:6199-206.
3. Eggermont AM, Robert C. New drugs in melanoma: It's a whole new world. Eur J Cancer. 2011.
4. Agarwala SS, Kirkwood JM, Gore M, Dreno B, Thatcher N, Czarnetski B, et al. Temozolomide for the treatment of brain metastases associated with metastatic melanoma: a phase II study. J Clin Oncol. 2004;22:2101-7.
5. Chapman PB, Einhorn LH, Meyers ML, Saxman S, Destro AN, Panageas KS, et al. Phase III multicenter randomized trial of the Dartmouth regimen versus dacarbazine in patients with metastatic melanoma. J Clin Oncol. 1999;17:2745-51.
6. Hersh EM, Del Vecchio M, Brown MP, Kefford R, Loquai C, Testori A, et al. A randomized, controlled phase III trial of nab-Paclitaxel versus dacarbazine in chemotherapy-naive patients with metastatic melanoma. Ann Oncol. 2015;26:2267-74.
7. Ribas A, Kefford R, Marshall MA, Punt CJ, Haanen JB, Marmol M, et al. Phase III randomized clinical trial comparing tremelimumab with standard-of-care chemotherapy in patients with advanced melanoma. J Clin Oncol. 2013;31:616-22.
8. Schadendorf D, Ugurel S, Schuler-Thurner B, Nestle FO, Enk A, Brocker EB, et al. Dacarbazine (DTIC) versus vaccination with autologous peptide-pulsed dendritic cells (DC) in first-line treatment of patients with metastatic melanoma: a randomized phase III trial of the DC study group of the DeCOG. Annals of oncology : official journal of the European Society for Medical Oncology / ESMO. 2006;17:563-70.
9. Chapman PB, Hauschild A, Robert C, Haanen JB, Ascierto P, Larkin J, et al. Improved survival with vemurafenib in melanoma with BRAF V600E mutation. N Engl J Med. 2011;364:2507-16.
10. Flaherty KT, Puzanov I, Kim KB, Ribas A, McArthur GA, Sosman JA, et al. Inhibition of mutated, activated BRAF in metastatic melanoma. N Engl J Med. 2010;363:809-19.
11. Long GV, Menzies AM, Nagrial AM, Haydu LE, Hamilton AL, Mann GJ, et al. Prognostic and clinicopathologic associations of oncogenic BRAF in metastatic melanoma. J Clin Oncol. 2011;29:1239-46.
12. Bauer J, Buttner P, Murali R, Okamoto I, Kolaitis NA, Landi MT, et al. BRAF mutations in cutaneous melanoma are independently associated with age, anatomic site of the primary tumor, and the degree of solar elastosis at the primary tumor site. Pigment Cell Melanoma Res. 2011;24:345-51.
13. Vogelstein B, Papadopoulos N, Velculescu VE, Zhou S, Diaz LA, Jr., Kinzler KW. Cancer genome landscapes. Science. 2013;339:1546-58.
14. Braiteh F, Kurzrock R. Uncommon tumors and exceptional therapies: paradox or paradigm? Mol Cancer Ther. 2007;6:1175-9.
15. Stewart DJ, Kurzrock R. Cancer: the road to Amiens. J Clin Oncol. 2009;27:328-33.
16. Curtin JA, Fridlyand J, Kageshita T, Patel HN, Busam KJ, Kutzner H, et al. Distinct sets of genetic alterations in melanoma. N Engl J Med. 2005;353:2135-47.
17. Stark MS, Dutton-Regester K, Woods SL, Gartside M, Bonazzi VF, Aoude LG, et al. Decreased MAP3K9/5 signaling through frequent somatic mutation or loss of heterozygosity contributes to Temozolomide resistance in metastatic melanoma. Nature Genetics. 2011:(In Press).
18. Yokoyama S, Woods SL, Boyle G, Aoude LG, MacGregor S, Zismann V, et al. A novel recurrent mutation in MITF predisposes to familial and sporadic melanoma. Nature. 2011:(In Press).
19. Comprehensive genomic characterization defines human glioblastoma genes and core pathways. Nature. 2008;455:1061-8.
20. Balakrishnan A, Bleeker FE, Lamba S, Rodolfo M, Daniotti M, Scarpa A, et al. Novel somatic and germline mutations in cancer candidate genes in glioblastoma, melanoma, and pancreatic carcinoma. Cancer Res. 2007;67:3545-50.
21. Heng HH. Cancer genome sequencing: the challenges ahead. Bioessays. 2007;29:783-94.
22. Hamburg MA, Collins FS. The path to personalized medicine. N Engl J Med. 2010;363:301-4.
23. Beskow LM, Friedman JY, Hardy NC, Lin L, Weinfurt KP. Developing a simplified consent form for biobanking. PLoS One. 2010;5:e13302.

24. Report of the President's Council of Advisors on Science and Technology (PCAST) website (accessed 2011 Mar 21). Priorities for Personalized Medicine. www.whitehouse.gov/files/documents/ostp/PCAST/pcast_report_v2.pdf. 2008 [cited March, 2011]; Available from: www.whitehouse.gov/files/documents/ostp/PCAST/pcast_report_v2.pdf
25. Garzon R, Fabbri M, Cimmino A, Calin GA, Croce CM. MicroRNA expression and function in cancer. Trends in molecular medicine. 2006;12:580-7.
26. National Cancer Institute. Best practices for biospecimen resources (accessed 2011 March 21). http://biospecimens.cancer.gov/global/pdfs/NCI_Best_Practices_060507.pdf. 2007.
27. Stewart DJ, Whitney SN, Kurzrock R. Equipoise lost: ethics, costs, and the regulation of cancer clinical research. J Clin Oncol. 2010;28:2925-35.
28. Qu KZ, Pan Q, Zhang X, Rodriguez L, Uyeji J, Li H, et al. Detection of BRAF mutations in melanoma: Rate of mutation detection at codon 600 using Sanger sequencing as compared to the cobas 4800 method. J Clin Oncol. 2012;30:abstr 8596.
29. Zhu X, Gerstein M, Snyder M. Getting connected: analysis and principles of biological networks. Genes Dev. 2007;21:1010-24.
30. Jones S, Zhang X, Parsons DW, Lin JC, Leary RJ, Angenendt P, et al. Core signaling pathways in human pancreatic cancers revealed by global genomic analyses. Science. 2008;321:1801-6.
31. Huang S. Back to the biology in systems biology: what can we learn from biomolecular networks? Brief Funct Genomic Proteomic. 2004;2:279-97.
32. Aranda-Anzaldo A. Cancer development and progression: a non-adaptive process driven by genetic drift. Acta Biotheor. 2001;49:89-108.
33. Wang E, Lenferink A, O'Connor-McCourt M. Cancer systems biology: exploring cancer-associated genes on cellular networks. Cell Mol Life Sci. 2007;64:1752-62.
34. Michor F, Nowak MA, Iwasa Y. Evolution of resistance to cancer therapy. Curr Pharm Des. 2006;12:261-71.
35. Sjoblom T, Jones S, Wood LD, Parsons DW, Lin J, Barber TD, et al. The consensus coding sequences of human breast and colorectal cancers. Science. 2006;314:268-74.
36. Von Hoff DD, Stephenson JJ, Jr., Rosen P, Loesch DM, Borad MJ, Anthony S, et al. Pilot study using molecular profiling of patients' tumors to find potential targets and select treatments for their refractory cancers. J Clin Oncol. 2010;28:4877-83.
37. Littman B, Thompson J, Webb CP. Where are we heading/What do we really need? In: Bleavins M, Rahbari R, Jurima-Romet M, Carini C, editors. Biomarkers in Drug Development: A Handbook of Practice, Application, and Strategy. New York: Wiley; 2010. p. 593-623.
38. Webb C. Personalized medicine: The need for system integration in the design of targeted therapies. In: Mazzarella R, Head R, editors. Computational and Systems Biology: Methods and Applications. Kerala, India: Research Signpost; 2009. p. 177-95.
39. Couzin-Frankel J. Personalized medicine. Pushing the envelope in neuroblastoma therapy. Science. 2011;333:1569-71.
40. Tsimberidou AM, Iskander NG, Hong DS, Wheler JJ, Fu S, Piha-Paul SA, et al. Personalized medicine in a phase I clinical trials program: The M. D. Anderson Cancer Center Initiative. J Clin Oncol. 2011;29:suppl; abstr CRA2500.
41. Overington JP, Al-Lazikani B, Hopkins AL. How many drug targets are there? Nat Rev Drug Discov. 2006;5:993-6.
42. Wishart DS, Knox C, Guo AC, Cheng D, Shrivastava S, Tzur D, et al. DrugBank: a knowledgebase for drugs, drug actions and drug targets. Nucleic Acids Res. 2008;36:D901-6.
43. National Bioethics Advisory Commission. Research involving human biological materials: Ethical issues and policy guidance, Volume 1. Rockville, MD: National Bioethics Advisory Commission. 115 p. 1999.
44. Monsma DJ, Monks NR, Cherba DM, Dylewski D, Eugster E, Jahn H, et al. Genomic characterization of explant tumorgraft models derived from fresh patient tumor tissue. J Transl Med. 2012;10:125.
45. Cella DF, Tulsky DS. Quality of life in cancer: definition, purpose, and method of measurement. Cancer investigation. 1993;11:327-36.
46. Cormier JN, Davidson L, Xing Y, Webster K, Cella D. Measuring quality of life in patients with melanoma: development of the FACT-melanoma subscale. The journal of supportive oncology. 2005;3:139-45.

47. Cormier JN, Ross MI, Gershenwald JE, Lee JE, Mansfield PF, Camacho LH, et al. Prospective assessment of the reliability, validity, and sensitivity to change of the Functional Assessment of Cancer Therapy-Melanoma questionnaire. Cancer. 2008;112:2249-57.
48. Eisenhauer EA, Therasse P, Bogaerts J, Schwartz LH, Sargent D, Ford R, et al. New response evaluation criteria in solid tumours: revised RECIST guideline (version 1.1). Eur J Cancer. 2009;45:228-47.
49. Green RC, Berg JS, Grody WW, Kalia SS, Korf BR, Martin CL, et al. ACMG Recommendations for Reporting of Incidental Findings in Clinical Exome and Genome Sequencing. 2013 [cited 2013 April 16]; Available from: http://www.acmg.net/docs/ACMG_Releases_Highly-Anticipated_Recommendations_on_Incidental_Findings_in_Clinical_Exome_and_Genome_Sequencing.pdf

25 List of Appendices

1.) Appendix I: Performance Status Criteria

2.) Appendix II: Study Pharmacopeia

3.) Appendix III: Blood Collection and Processing for Extraction of Constitutional Analytes at Ashion Analytics a central CLIA-certified laboratory affiliated withTGen

4.) Appendix IV: Fresh Frozen Tumor Collection and Shipment

5.) Appendix V: Collection/handling/shipping of fresh tissue for tumorgraft implanting

6.) Appendix VI: Shipping of paraffin blocks and slides

7.) Appendix VII: Collection and Shipping of Plasma for Circulating Tumor Nucleic Acids (ctNA)

8.) Appendix VIII: Cryopreservation of Tissue in OCT (Proteomics)

9.) Appendix IX: Merck Sharp & Dohme

10.) Appendix X: Sample Distribution from Clinical Sites

11.) Appendix XI: Acknowledgment of the Investigators

12.) Appendix XII: Description of Tumor Boards

13.) Appendix XIII: Drug Specific Appendices

Appendix I: Performance Status Criteria

ECOG Performance Status Scale	
Grade	Descriptions
0	Normal activity. Fully active, able to carry on all pre-disease performance without restriction.
1	Symptoms, but ambulatory. Restricted in physically strenuous activity, but ambulatory and able to carry out work of a light or sedentary nature (*e.g.*, light housework, office work).
2	In bed <50% of the time. Ambulatory and capable of all self-care, but unable to carry out any work activities. Up and about more than 50% of waking hours.
3	In bed >50% of the time. Capable of only limited self-care, confined to bed or chair more than 50% of waking hours.
4	100% bedridden. Completely disabled. Cannot carry on any self-care. Totally confined to bed or chair.
5	Dead.

Appendix II: Study Pharmacopeia

Table 1. Commercial Agents Available For Use		
**Drug Name:**	**Target:**	**Route**
Adriamycin	DNA, TOP2A	IV
Bortezomib-TBD	proteasomes	IV, Sub-Q
Carboplatin	DNA	IV
Dacarbazine	POLA2	IV
Dasatinib	C-Src	Oral
Erlotinib	EGFR, NR1I2	Oral
Etoposide	topoisomerase-2	IV, Oral
Gemcitabine	RRM1, TYMS, CMPK, DNA	IV
Imatinib	Multikinase (BCR-ABL, c-kit, PDGF-R, RET)	Oral
Interferon, Recombinant	IFNAR2, IFNAR1	IV, Sub-Q
Paclitaxel	TUBB1, BCL2	IV
Pemetrexed	TYMS, ATIC, DHFR, GART	IV
Sorafenib	BRAF, RAF1, VEGFR2, VEGFR3, FLT3, PDGFRB, KIT, FLT4	Oral
Temozolomide	DNA	Oral
Vorinostat	pan-histone deacetylase inhibitor	Oral

Table 2. Commercial Agents Provided by Company				
**Company Name:**	**Drug Name:**	**Target:**	**Route**	**IND#**
Exelixis	XL184 (cabozantinib)	Multi-kinase (VEGFR2, Met, FLT3, Tie2, Kit and Ret)	Oral	72,596 113,446
Pfizer	PD-0332991 (palbociclib)	CDK 4/6 inhibitor	Oral	69,324
	Inlyta (axitinib)	VEGFR	Oral	N/A
	Bosulif (bosutinib)	Abl, Src	Oral	N/A
	Sutent (sunitinib)	PDGFRa, PDGFRb, VEGFR1, VEGFR2, VEGFR3, KIT, FLT3, CSF1R, RET	Oral	N/A
	Torisel (temsirolimus)	FRAP1	IV	N/A
	Xalkori (crizotinib)	ALK, ROS1, MET	Oral	N/A

Table 3. Investigational Agents Available For Use				
**Company Name:**	**Drug Name:**	**Target:**	**Route**	**IND#**
Millennium	MLN8237	Aurora A kinase	Oral	75,473
	MLN9708	proteasome protease inhibitor	Oral	104,482
Pfizer	PF-00299804	pan-erbB	Oral	72,775
Plexxikon	Pexidartinib (PLX3397)	FMS, Kit and Fit3-ITD	Oral	105,521
Novartis	BGJ398	FGFR 1/2	Oral	104,187
Array BioPharma	MEK162 (Binimetinib)	MEK 1/2	Oral	109,560

Table 4. Investigational Agents Awaiting RP2D and/or Company Review				
**Company Name:**	**Drug Name:**	**Target:**	**Route**	**IND#**
Eli Lilly	LY2157299	TGF-β Receptor I Kinase Inhibitor	Oral	
Millennium	MLN1117	PI3Kα	Oral	112048
	MLN0128	TORC 1/2	Oral	104801
	MLN 2480	PanRAF	Oral	108340

Appendix III: Blood Collection and Processing for Extraction of Constitutional Analytes at Ashion Analytis a central CLIA-certified laboratory affiliated with TGen

Blood samples will be drawn from participants that have been through the informed consent process for participation in the research study. Blood samples will be obtained by personnel qualified to draw blood from participants at the collection site. The purpose of this document is to outline standardized procedures for Collection Sites to follow for blood collection.
Collection kits containing all relevant clinical data collection forms, system-generated barcodes to pre-label tissue and blood collection tubes, shipping manifests, return labels and materials, and appropriate transport documentation will be assembled by trained shipping technicians within the TGen repository. The system-generated barcodes will represent global specimen identifiers that will be tracked by a biobanking software solution hosted by TGen. The system allows the assignment of multiple, unique identifiers to a single biospecimen, and the system can generate and assign a new, unique barcode label for a biospecimen received from a clinical site that may arrive with barcode labels specific to their institution. The system records the new barcode identifier along with the clinical site-specific barcode identifier and the global specimen identifier.

Clinical sites will be provided with SOPs detailing the proper packaging and shipping vessels to be employed for each sample type to protect them from loss, damage, and temperature variations during shipment. These protocols include the use of insulated packing material, refrigerated gel packs, frozen gel packs, dry ice pellets, and liquid nitrogen as appropriate.

I. SAFETY

Always use universal precautions when dealing with any blood samples. Dispose of all blood collection equipment in the appropriate receptacles at the collection site.

II. MATERIALS & EQUIPMENT

1. Tourniquet
2. Alcohol Swab
3. Phlebotomy needle
4. Gauze
5. Pre-labeled purple top EDTA blood collection tubes
6. Adhesive bandage

III. PROCEDURES

This procedure is intended to ensure that blood samples will be obtained from consented participants in a safe and efficient manner while eliminating the risks of contamination. Patient identifiers will remain under HIPAA compliance, and clinical information will be kept in accordance with each site's standard practice. Sample collection kits containing barcoded tubes for shipping samples to the appropriate laboratory will be provided to the collection sites.

A. Timing for Blood Collection

1. Preferably, if the participant will be undergoing surgery, blood collection should be done pre-operation and as close as possible to the time when the tissue is collected for the research study.
2. Identify the person responsible for processing the blood.
3. Contact this person before or soon after blood collection to arrange timely processing of the sample.

B. Blood Collection Procedure – Preparation

1. Blood collection must be performed by personnel qualified to draw blood.
2. Prior to blood collection, identify the participant, verify identification, and check that informed consent has been obtained.
3. Ensure that the barcoded labels on the blood collection tube match the barcode on the informed consent document.
4. Assess participant's physical and mental disposition and determine if this is the appropriate time to draw blood.
5. Be courteous, professional, and sensitive to the participant's needs. Ensure that all communications are discreet and respectful of participant confidentiality.
6. Assemble proper equipment to draw blood. See section IV, Equipment and Materials.

C. Blood Collection Procedure - Venipuncture

1. Provide for the participant's comfort as much as possible, and gain the participant's cooperation. Position the participant. The participant should sit in a chair, lie down or sit up in bed. Hyperextend the participant's arm.
2. Apply tourniquet to expose veins. Do not place too tightly. If superficial veins are not easily apparent, force blood into the vein by massaging the arm from wrist to elbow, tap the site with index and second finger, apply a warm, damp cloth to the site or lower extremity to allow veins to fill.
3. Select appropriate site for venipuncture. Avoid areas with excessive scars or hematomas. While hand and wrist veins are acceptable it is optimal to select an antecubital vein.
4. Prepare the participant's arm using an alcohol prep. Cleanse in a circular fashion, beginning at the site and working outward. Allow to air dry.
5. Anchor the vein and swiftly insert the needle (at a 15-30 degree angle with the surface of the arm) into the lumen of the vein. Avoid excessive probing and trauma to the site.
6. Draw blood 10-20 mL into an evacuated purple top EDTA blood collection tube.
7. When the last tube to be drawn is filling, remove the tourniquet.
8. Remove the needle from the participant and apply a gauze and adequate pressure to the site of venipuncture to avoid hematoma formation.
9. If needed, apply an adhesive bandage to the venipuncture site.
10. Dispose of needles and supplies in a safe manner.
11. Samples should be slowly inverted 8 to 10 times to ensure the mixing of the sample and the anti-coagulant liquid inside the tube.
12. Recheck that the barcode label on the blood collection tube matches the barcode on the informed consent document.
13. Complete the Ashion Analytics Specimen Submission and Requisition Form. Place a patient barcode label on the submission form. The barcode on the Ashion Analytics Specimen Submission and Requisition Form should match the barcode on the informed consent document and the blood collection tubes.
    a) Send the original form with the specimen.
    b) Keep a copy of the requisition in the patient's study binder if applicable.
14. Ship immediately with a cool pack for overnight delivery with arrival Monday-Friday. Specimens can be refrigerated for 7 days before shipping if needed.

D. Shipping Blood to Ashion Analytics

NOTE: Ship Monday through Thursday only unless prior notification is made is made with TGen. Do not ship the day before a U.S. Holiday.

1. Verify the barcode label matches the barcode on the Ashion Analytics Specimen Submission and Requisition Form.
2. Place the blood vacutainer(s) in a leak-proof biohazard bag containing absorbent material.
3. Place frozen ice/cooler packs in the bottom of a Styrofoam cooler. Place biohazard bag in the center of the cooler on top of the frozen ice/cooler packs, and then place another frozen ice/cooler pack(s) on top. Place the lid on the cooler and tape the lid tightly to the cooler, sealing all the way around the lid.
4. Place the cooler in the cardboard box, placing all paperwork associated with the case on top of the cooler, and tape shut.
5. The outermost container must be marked with the words Exempt Human Specimen (use labels or write by hand when necessary).
6. The U.S. DOT does not require these labels; however, IATA does require these labels. Therefore, include these labels on all packages in this category to streamline processes. Do not put the universal biohazard symbol on the outside of an exempt package as this may cause confusion regarding classification.
7. Verify that the FedEx air bill is marked **Standard Overnight Shipping**
8. Call Courier Service to pick up specimens
9. Ship Blood to TGen at the following address:

Ashion Analytics, LLC
445 North 5th St. Suite 468
Phoenix, AZ 85004
602-343-8796
602-343-8545

Appendix IV: Fresh Frozen Tumor Collection and Shipment

The purpose of this Standard Operating Procedure (SOP) is to outline the process for snap freezing tissue for processing and storage in Ashion Analytics. Tissue samples will be collected from participants who have been properly consented and who have agreed to participate in the research study. Tumor tissues are only suitable for molecular studies if frozen in a timely and appropriate manner. The purpose of this document is to outline standardized procedures for collection sites to follow for snap freezing tissue.

I. SAFETY

A. Wear personal protective equipment (PPE), such as lab coats and gloves when handling liquid nitrogen.

B. Liquid nitrogen is extremely cold and can cause 'burns'. Wear gloves that are specially made to withstand liquid nitrogen, as well as eye protection and a lab coat to protect skin from splashes and spills.

II. MATERIALS & EQUIPMENT

1. Container with dry ice (for transport of frozen tissue)
2. Clean forceps
3. Liquid nitrogen
4. 2-methylbutane (isopentane)
5. Container for isopentane
6. Pre-labeled cryovials
7. Dry shipper
8. FedEx Airway Bill
9. Biohazardous bag for shipping
10. Dry Ice
11. Personal protective equipment (PPE) to include gloves, lab coat.
12. Ashion Analytics Specimen Collection and Requisition Form

III. PROCEDURES

This procedure is intended to ensure that tissue samples collected from consented participants will be frozen in a safe and efficient manner while eliminating the risks of contamination and loss of molecular integrity. To facilitate the use of molecular techniques, tissue that has been adequately frozen is vital to obtaining products with high integrity and quality.

For each biopsy, we will collect five to six, 1-2 centimeter 16- or 18-gauge core needle specimens from accessible tumor. Depending on need (and as specified by the Principal Investigator for each patient enrolled on this study), either two or three cores will be placed in externally-threaded cryovials and immediately flash frozen in liquid nitrogen and stored at -80°C until shipping on dry ice to Ashion Analytics at TGen in Arizona. Other tissue cores will be cryopreserved in Optimal Cutting Temperature (OCT) compound (see Appendix VII), formalin-fixed and paraffin-embedded (see Appendix VI), and/or placed in media for eventual tumorgraft implantation (see Appendix V).

A. Tissue Priority

In cases where there is insufficient accessible tumor for collection of five core needle biopsies, sample processing will be prioritized as follows:

<u>FIRST BIOPSY</u>

1. Flash frozen core for DNA analysis (to be sent to Ashion; see Appendix IV);
2. Flash frozen core for RNA analysis (to be sent to Ashion; see Appendix IV);
3. Flash frozen core for correlative analyses or biobanking (to be sent to Ashion; see Appendix IV) **OR** fresh core biopsy in transport media (NOT FROZEN) for tumorgraft development (to be sent to Mayo Clinic in Arizona). Exact use of this core will be based on need and determined by the Principal Investigator for each individual patient enrolled on this study (see Appendix V);
4. Cryopreservation of Tissue in OCT for proteomics (to be sent to George Mason University; see Appendix VII)
5. Core for creation of formalin-fixed paraffin-embedded (FFPE) block (to be fixed/embedded at clinical site and sent to Ashion; see Appendix VI)
6. Any extra tissue will be flash frozen and biobanked (to be sent to Ashion; see Appendix IV)

SECOND BIOPSY

1. Flash frozen core for DNA analysis (to be sent to Ashion; see Appendix IV);
2. Flash frozen core for RNA analysis (to be sent to Ashion; see Appendix IV);
3. Cryopreservation of Tissue in OCT for proteomics (to be sent to George Mason University; see Appendix VII)
4. Core for creation of formalin-fixed paraffin-embedded (FFPE) block (to be fixed/embedded at clinical site and sent to Ashion; see Appendix VI)
5. Flash frozen core for correlative analyses or biobanking (to be sent to Ashion; see Appendix IV)
6. Any extra tissue will be flash frozen and biobanked (to be sent to Ashion; see Appendix IV)

B. Snap freezing of Tumor Tissue

1. Treat all tissue as potentially infectious.
2. Freezing is performed by research study staff as designated by the collection site principal investigator.
3. Have materials and equipment ready for tissue processing prior to surgery. Have pre-labeled cryovials ready.
4. Fresh tumor tissue should be frozen as soon as possible. Optimally, tissue should be frozen within 30 minutes from biopsy.
5. Do not freeze the tissue directly on ice.
6. Ensure that the biopsied tissue never desiccates or is contaminated by surrounding tissue or other samples. Use clean forceps between samples to avoid cross contamination. Do not place the sample in contact with formalin at any point in the process. Do not add serum to the sample.
7. Cool isopentane by suspending the container of isopentane in liquid nitrogen.
8. Isopentane is sufficiently cooled when “pearls” form and the solution becomes hazy.
9. With clean forceps, place the specimen to be frozen into an empty screw capped cryovial.
10. Close the cryovial.

11. Place the cryovial with the specimen into the container of cooled isopentane.
12. The specimen should freeze within 30 seconds.
13. Alternatively, the isopentane freezing step can be optional. Place the tissue specimen into an empty cryovial, close the cryovial, and immediately submerge the cryovial into liquid nitrogen. The specimen should freeze within 30-60 seconds. This is not recommended if the sample is large in size, as longer freezing time will result in ruined morphology.
14. Once snap frozen, samples should be packaged with dry ice and immediately shipped to Ashion Analytics.
15. If samples cannot be immediately shipped, samples should be placed on dry ice to be carried to the freezer or liquid nitrogen storage facility for storage until shipping.
16. Complete the Ashion Analytics Specimen Submission and Requisition Form. Place a patient barcode label on the submission form. The barcode on the Ashion Analytics Specimen Submission and Requisition Form should match the barcode on the informed consent document and the tissue collection cryovials.
    a) Send the original form with the specimen.
    b) Keep a copy of the requisition in the patient's study binder if applicable.

C. Fresh Frozen Tissue shipment to Ashion Analyics at TGen

NOTE: Ship Monday through Thursday only unless prior notification is made is made with TGen. Do not ship the day before a U.S. Holiday.

1. Verify the barcode label matches the barcode number of the Ashion Analytics Specimen Submission and Requisition form.
2. Place cryovial containing frozen specimen in biohazard bag.
3. Place 2-3 inches of dry ice in the bottom of a Styrofoam cooler. Place biohazard bag in the center of the cooler on top of the dry ice, and then fill the cooler the rest of the way with dry ice (preferably pelleted). Place a single paper-towel or piece of paper across top of ice, then put lid on the cooler and tape the lid tightly to the cooler, sealing all the way around the lid.
4. Place the cooler in the cardboard box, placing all paperwork associated with the case on top of the cooler, and tape shut.
5. The outermost container must be marked with the words Exempt Human Specimen (use labels or write by hand when necessary).
6. The U.S. DOT does not require these labels; however, IATA does require these labels. Therefore, include these labels on all packages in this category to streamline processes. Do not put the universal biohazard symbol on the outside of an exempt package as this may cause confusion regarding classification.
7. The outermost container must be labeled with a hazard class 9 label, UN1845, and net weight of dry ice in kilograms. The label should be affixed to a vertical side of the box (not the top or bottom) and orientated as shown in the picture in Appendix V. The maximum allowable net quantity of dry ice allowed per package is 200kg.
8. Verify the following on the FedEx air bill:
    a) Standard Overnight Shipping

b) The Airbill must include the statement “Dry ice, 9, UN1845, number of packages X net weight in kilograms”. FedEx has a check box on their Airbill to satisfy their requirement.

9. Call Courier Service to pick up specimens
10. Ship frozen tissue to:

Ashion Analytics, LLC
445 North 5th St. Suite 468
Phoenix, AZ 85004
602-343-8796
602-343-8545

Shipment Packing Instructions for Frozen Shipments

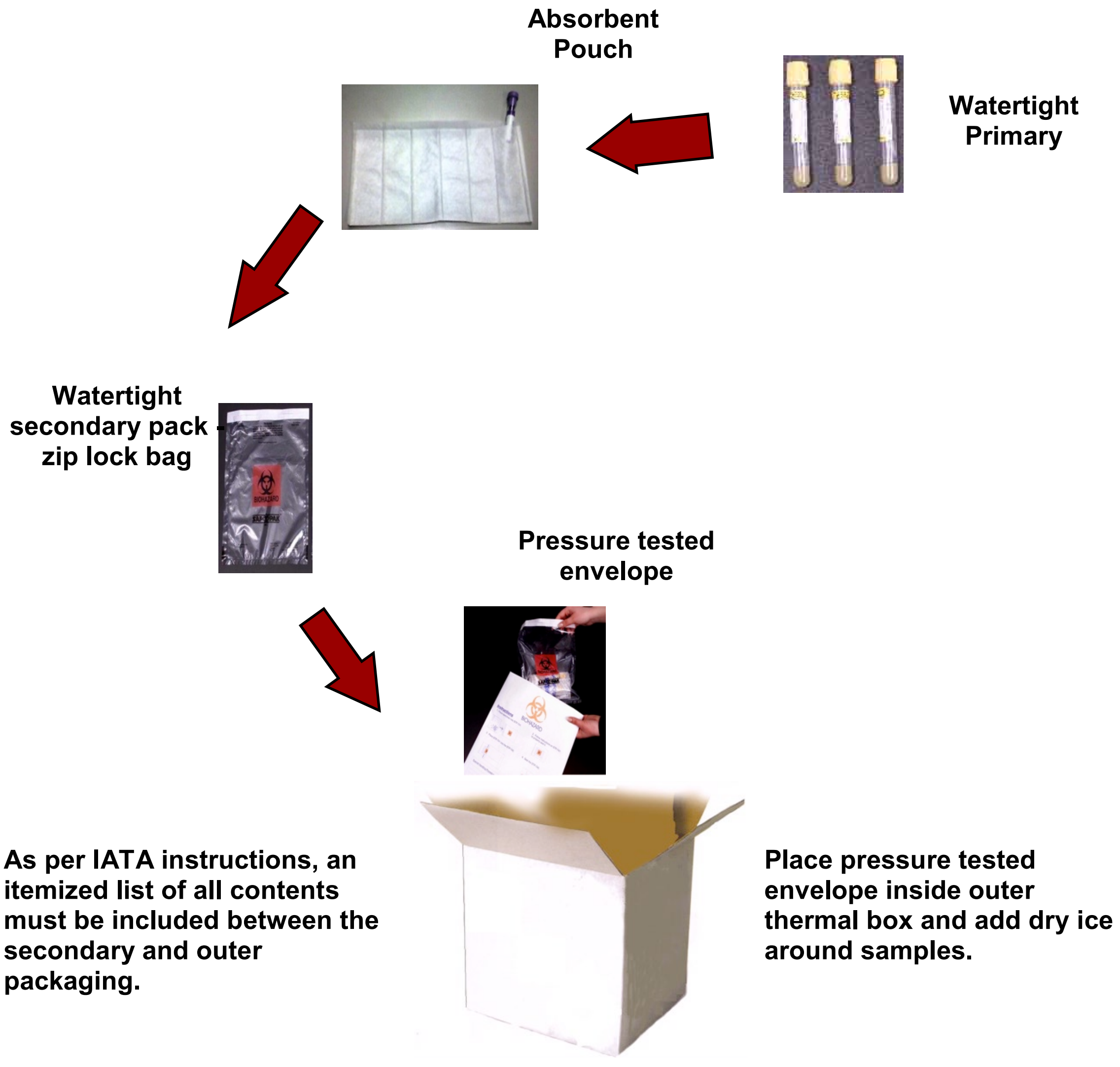

Shipment Packaging Instructions for Frozen Specimen

Appendix V: Collection/handling/shipping of fresh tissue for tumorgraft implanting

IMPORTANT: verify with Principal Investigator prior to biopsy whether a core tissue sample is to be designated for tumorgraft implantation, or whether it should be flash frozen for correlative analyses or biobanking.

Supplies:

1. Transport medium: RPMI1640 media (e.g. Invitrogen, Cat. #21870-076) + 10% FBS (e.g. Invitrogen, Cat. #16000-044) + 1% Penicillin/Streptomycin (e.g. Invitrogen, Cat. #151410-122) + Heparin 50 units/mL (e.g. Sigma, Cat. #H3149-25KU)
2. Sterile 15 mL conical tubes (e.g. Greiner, Cat. #188271 or Corning, Cat #43055)
3. Parafilm (e.g. Fisher Scientific, Cat. #13-374-10)
4. IATA 650 compliant insulated shipping container
5. Refrigerant gel packs (e.g. Polar Tech Ind., Cat. #1B-8)
6. Biohazard bag (e.g. Fisher, Cat. #01828D)

Sample packaging:

Note: Fresh biopsy tissue for use in tumorgraft implanting must be handled under sterile conditions. Prepare and handle all materials in a biological safety cabinet using sterile technique.

1. Label a sterile 15 mL conical tube with the protocol number, the de-identified tumor tissue ID, and the date of tissue harvest.
2. Add 8 mL of transport medium to the 15 mL conical tube.
3. Place the tissue sample in the 15 mL tube containing the transport media, close the tube cap tightly and seal the cap with Parafilm.
4. Place the 15 mL tube containing the tissue tissue in a biohazard bag and seal. Place the biohazard bag containing the 15mL tube between two refrigerated cold packs and secure in a plastic bag.
5. Place frozen cold packs to surround the sample in the Styrofoam container.
6. Fill empty space in Styrofoam container with bubble wrap or packing paper to avoid shifting of the contents. Prepare a shipping manifest that details the contents of the shipment such that individual samples can be readily identified.
7. Place the Styrofoam box in the cardboard box and place a copy of the shipping manifest on top of the Styrofoam box (between the Styrofoam box and the outer cardboard box).
8. Seal the cardboard box with tape.
9. The outermost container must be marked with the words Exempt Human Specimen (use labels or write by hand when necessary).
   Note: Do not put the universal biohazard symbol on the outside of an exempt package since the tissue is not considered a biohazardous material and is, therefore, exempt from these shipping regulations.

Sample Shipping:

NOTE: [Ship Monday through Thursday only unless prior notification is made is made with Mayo Clinic Laboratory Animal Facility. Do not ship the day before a U.S. Holiday.]

- **Complete the air bill (numbered items are labeled on the sample air bill below) for Standard Overnight shipping:**
  1. Date of shipment
  2. The sender's name and the company's address
  3. Recipient's address - ship to the following address:

     Attn: Jessica E. Schmidt/Aleksandar Sekulic
     Mayo Clinic Arizona
     13400 East Shea Boulevard
     MCCRB 3-040 Sekulic lab
     Scottsdale, AZ 85259
     Phone: (480) 301-6796
     Email: Schmidt.Jessica1@mayo.edu

  4. Check the box for "**Yes. Shippers Declaration not required**"
  5. Enter the total weight of the package (note: this cannot be pre-printed and **must** be filled in by the sender)

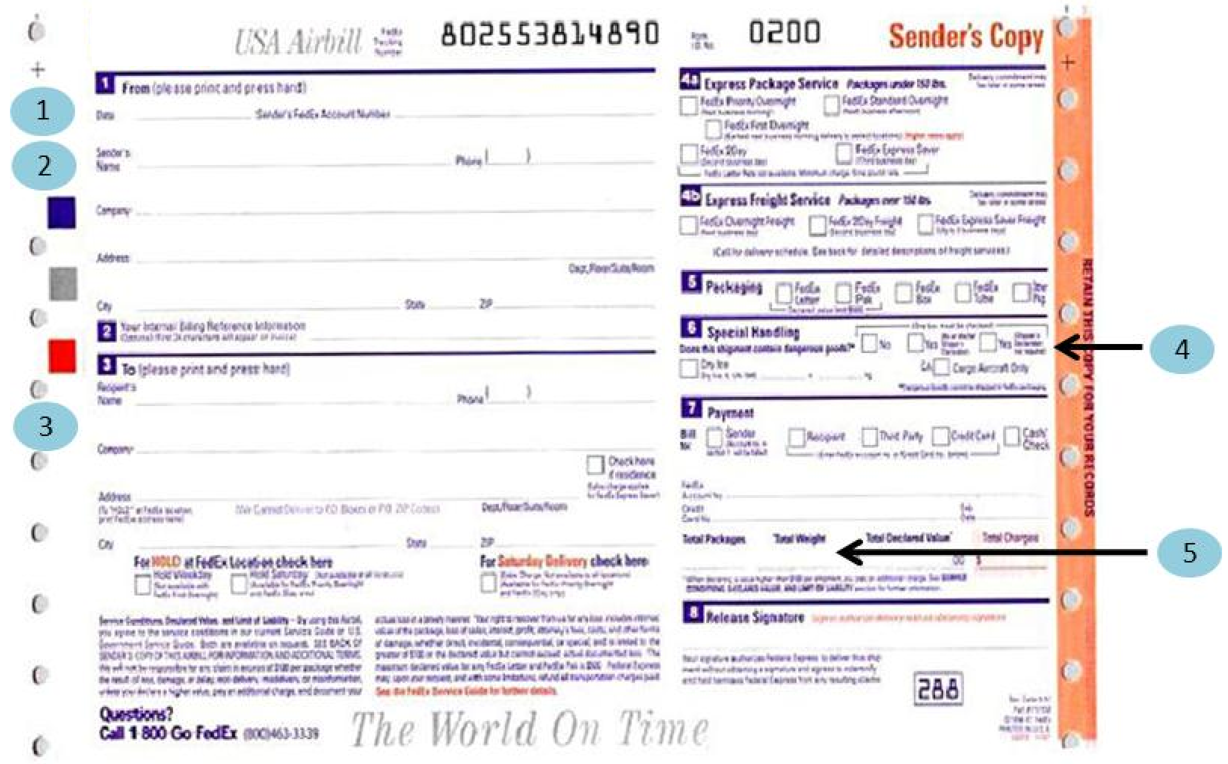

- **Affix the completed air bill to the top of the box**
- Contact the local Federal Express office to arrange for diagnostic specimen pick up.

- **Pick-up must be the same day as packaging. Do not leave the box for pick-up the next day**, as this may severely compromise tumor tissue viability and subsequent tumorgraft development.

Notify the Recipient about the Shipment

- Please email Aleksandar Sekulic (sekulic@mayo.edu) and Jessica Schmidt (Schmidt.Jessica1@mayo.edu) when the samples are shipped
- Include which samples are included in the shipment and the FedEx tracking number in the email

Fresh Tissue Tumor Manifest

This information will be filled out and shipped along with the fresh tumor tissue for tumorgraft establishment.

	**Sample #**	**Sample #**
Site		
Investigator		
Date		
Patient study ID		
Anatomical location		
Type of tissue (e.g. 18 gauge core)		
Size of cores (mm)		
Notes		

Appendix VI: Shipping of paraffin blocks and slides

Shipping FFPE and Slides to Ashion Analytics at TGen

At the clinical site, a single 1-2 centimeter 16- or 18-gauge core needle specimen will be formalin-fixed and paraffin-embedded at the clinical site and the block shipped to TGen for longitudinal analyses.

If available, we will also collect archival (diagnostic) tissue for research purposes (either archival paraffin tissue block or 10 unstained slides of a primary or metastatic cancer lesion– initial metastasis preferred. Archival tissue collected prior to immunotherapy whenever possible) prior to enrollment. Samples should be shipped within 3 months after enrollment.

Instructions

1. Verify the sample label(s) match the barcode number of the Specimen Submission Form.
2. If sending an FFPE block, place the FFPE block in a ziplock bag and wrap in bubble wrap or another type of padded material prior to shipping. The zip lock bag can be placed into a FedEx envelope or small shipping box.
3. If sending slides, pack the labeled slides into plastic slide cassette(s). Tape plastic slide cassettes shut and wrap in bubble wrap or another type of padded material prior to shipping. Place the slide cassettes into an outer shipping box.
4. Verify that the FedEx air bill is marked **Standard Overnight Shipping.**
5. Call Courier Service to pick up specimens.
6. Ship Specimens to TGen:
   Ashion Analytics, LLC
   445 North 5th St. Suite 468
   Phoenix, AZ 85004
   602-343-8796
   602-343-8545 (fax)

Appendix VII: Collection and Shipping of Plasma for Circulating Tumor Nucleic Acids (ctNA)

Cell-free nucleic acid fragments contributed by a tumor into blood can be measured using molecular assays for cancer-specific somatic mutations. Circulating tumor nucleic acids (ctNA) can be an inherently cancer-specific biomarker for solid cancers such as melanoma, allowing non-invasive, repeatable sampling of the cancer genome while potentially overcoming spatial intra-tumor heterogeneity. Within the context of the SU2C trial, ctNA analysis can complement current strategies for:

4) molecular cancer stratification at presentation and at relapse
5) measurement of systemic tumor burden to assess initial tumor response and to monitor for recurrence/progression
6) assessment of sub-clonal tumor dynamics, treatment-driven cancer evolution and acquired resistance

A key strength of ctNA analysis is the ability to acquire longitudinal samples with minimal discomfort to a cancer patient. Blood samples will be collected at the following timepoints for each patient: day of initial biopsy (prior to actual biopsy procedure); Cycle 1, Day 1 (prior to treatment); Cycle 1, Day 8 or 15 (depending on which date their scheduled visit will occur, prior to treatment); Day 1 of each subsequent cycle (prior to treatment), and at the time of second biopsy (end of study/progression, prior to actual biopsy procedure) for preparation of plasma for analysis. Patients who cross-over to molecularly guided treatment will also have blood collected at the following timepoints: Cycle 1, Day 1 (prior to molecularly guided treatment); Cycle 1, Day 8 or 15 (depending on which date their scheduled visit will occur, prior to treatment); Day 1 of each subsequent cycle (prior to treatment), and at Off-Study visit for preparation of plasma and buffy coat (peripheral blood cells) for analysis. For visits while the patient is on treatment, samples are best obtained prior to administration of drug on each visit. For visits while the patient is undergoing a biopsy, samples must be obtained prior to the biopsy procedure. **NOTE:** once processed as described below, plasma samples can be stored at -70^0 to -80^{0}C indefinitely and should be sent in a batch shipment when the patient comes off study to save shipping costs.

Procedure for plasma collection:

Blood samples should be collected in EDTA tubes. It is critical that blood samples are centrifuged twice, as described, as soon as possible and within one hour of collection to minimize the possibility of white blood cell lysis that can affect our ability to measure ctNA.

1) Collect blood in 10mL hematology EDTA tube
2) Label the tube with study/patient identifiers, stating time of collection.
3) Gently invert the tube 8-10 times to mix and leave upright prior to centrifugation.
4) As soon as possible and within one hour, centrifuge the samples at 820g for 10 mins at room temperature, with the centrifuge brake off. Record time of centrifugation.
5) Transfer 1mL aliquots of the plasma to sterile 2mL micro-tubes. Take care to avoid any buffy coat layer in this step.
6) Label each aliquot by the order in which it was isolated, 1 thru *n* for *n* aliquots.
7) Carefully transfer the buffy coat layer into a different sterile 2mL micro-tube and label appropriately.
8) Centrifuge plasma aliquots in a bench top centrifuge at maximum speed (16,000-20,000g) for 10 mins to pellet any remaining cellular debris.
9) Carefully transfer 1ml aliquots of supernatant to sterile 2mL screw-capped micro-tubes and discard the pellet.

10) Label tubes appropriate with barcodes.
11) Freeze plasma aliquots at -80^{0}C freezer in appropriate locations. **NOTE:** samples can be stored at -70^0 to -80^{0}C indefinitely and should be sent in a batch shipment when the patient comes off study to save shipping costs.
12) Record corresponding information such as collection time, centrifugation time, freezing time, duration of centrifugation at each step and aliquot number (as described in step 6 above).
13) Record any additional handling or quality information such as hemolysis etc.

Buffy coat and plasma samples are to be collected at every time point.

ctNA Shipping Instructions

NOTE: samples can be stored at -70^0 to -80^{0}C indefinitely and should be sent in a batch shipment when the patient comes off study to save shipping costs.

1. Verify the barcode label matches the barcode number of the Ashion Analytics Specimen Submission and Requisition form.
2. Place screw-capped micro-tubes containing frozen aliquots in biohazard bag.
3. Place 2-3 inches of dry ice in the bottom of a Styrofoam cooler. Place biohazard bag in the center of the cooler on top of the dry ice, and then fill the cooler the rest of the way with dry ice (preferably pelleted). Place a single paper-towel or piece of paper across top of ice, then put lid on the cooler and tape the lid tightly to the cooler, sealing all the way around the lid.
4. Place the cooler in the cardboard box, placing all paperwork associated with the case on top of the cooler, and tape shut.
5. The outermost container must be marked with the words Exempt Human Specimen (use labels or write by hand when necessary).
6. The U.S. DOT does not require these labels; however, IATA does require these labels. Therefore, include these labels on all packages in this category to streamline processes. Do not put the universal biohazard symbol on the outside of an exempt package as this may cause confusion regarding classification.
7. The outermost container must be labeled with a hazard class 9 label, UN1845, and net weight of dry ice in kilograms. The label should be affixed to a vertical side of the box (not the top or bottom) and orientated as shown in the picture in Appendix V. The maximum allowable net quantity of dry ice allowed per package is 200kg.
8. Verify the following on the FedEx air bill:
    a. Standard Overnight Shipping
    b. The Airbill must include the statement “Dry ice, 9, UN1845, number of packages X net weight in kilograms”. FedEx has a check box on their Airbill to satisfy their requirement.
9. Call Courier Service to pick up specimens
10. Ship blood to:

    Tania Contente-Cuomo,
    Translational Genomics Research Institute
    445 N. 5th Street
    Phoenix, AZ 85004
    Phone: 602-343-8673
    Email: tcontente-cuomo@tgen.org

Shipment Packing Instructions for Frozen Shipments

Watertight Primary

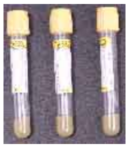

Absorbent Pouch

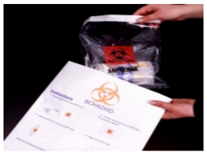

Watertight secondary pack zip lock bag

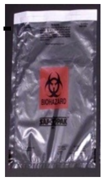

Pressure tested envelope

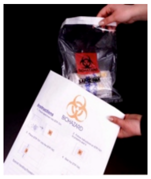

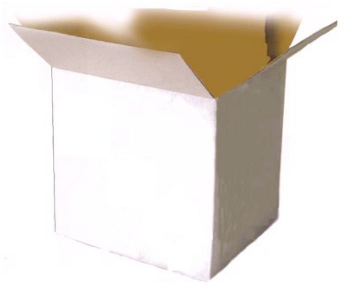

As per IATA instructions, an itemized list of all contents must be included between the secondary and outer packaging.

Place pressure tested envelope inside outer thermal box and add dry ice around samples.

Shipment Packaging Instructions for Frozen Specimen

Appendix VIII: Cryopreservation of Tissue in OCT (Proteomics)

Purpose: To describe the preservation of core needle biopsy samples for molecular analysis.

Principle: Core needle biopsies are used to sample tissue from a specific, defined location. These biopsies may consist of normal, pre-malignant and malignant tissue due to the multi-level tissue sample that is obtained. This type of sample is ideal for studying the micro-tumor environment.
Cellular RNA and proteins are extremely labile components of the cellular makeup and probably the most commonly analyzed in the molecular laboratory. Proper and expedient handling of a specimen for analysis has a great impact on the quality and quantity of the RNA and proteins that are remaining and available for extraction. While there appears to be great variation in the degradation times between tissue types, the tissue sample should always be frozen as soon as possible to provide the best results. Optimal Cutting Temperature (OCT) compound is an alcohol polymer that is liquid at room temperature and a solid at –20°C. This polymer is used to cryo-protect the tissue and provide a medium for cryo-sectioning. **NOTE:** samples can be stored at -70^{0} to -80^{0}C indefinitely and should be sent in a batch shipment to save shipping costs.

Materials:
Cryomolds (Sakura Finetek Cat. # 4728)
OCT (Sakura Finetek Cat. # 4583)
Dry ice
Ultra cold freezer (-70^{0} to -80^{0}C)
Needle: 16 or 18 gauge
Permanent marker
Sterile forceps
Sterile Glass slides
Aluminum foil or 50ml Falcon tubes

Procedure:
1. Prepare all supplies prior to the biopsy procedure to avoid delay once the specimen has been obtained.
2. Label the handle and the front surface of the cryomold with the sample or patient's identifying information.
3. Perform core needle biopsy.
4. Pick the core from the biopsy needle onto a sterile glass slide.
5. Fill cryomold about 1/3 full with OCT. Place the cryomold in dry ice to freeze the OCT. The OCT should be completely frozen.
6. Carefully lift the core biopsy by both ends with sterile forceps. Do not stretch the biopsy or it will break.
7. Lay the biopsy as straight as possible in the OCT. Once the sample touches the OCT, you cannot reposition it or the sample will break apart.
8. Quickly add OCT on top of the biopsy, completely covering the sample.
9. Ensure the sample is level and freeze immediately in dry ice.
10. Store wrapped in aluminum foil or in a 50ml Falcon tube at –70°C to -80^{0}C. If sample does not fit in 50ml Falcon tube, sample can be stored in a plastic bag.

Frozen Section Slides

1. Frozen sections for proteomic analysis should be cut at 5-8um on plain, uncoated glass microscope slides.
2. The tissue section should be placed as close as possible to the center of the slide. Do not place the frozen section at the end of the slide.
3. Two tissue sections from the same biopsy may be placed on the same glass slide if space permits.
4. Do not allow the tissue section to air on the slide. Freeze immediately on dry ice or at –80°C. **NOTE:** samples can be stored at -70^0 to -80^0C indefinitely and should be sent in a batch shipment to save shipping costs.

Shipping Slides or Frozen Tissue

1. Ship slides/tissue in OCT on dry ice Monday through Thursday only.
2. Tissue should be embedded in OCT prior to shipment. Refer to procedure steps 5-10.
3. Seal slide box in a plastic bag with desiccant (such as Drierite crystals). Seal tissue in a 50ml polypropylene Falcon tube, wrap in aluminum foil or place in a plastic bag.
4. Place dry ice on top of the plastic bag containing the slides/tissue.
5. Place any special instructions/inventory/shipping documents in the box.
6. Seal the box with tape.
7. Follow standard shipping protocols for your institution.

Send the OCT embedded core and/or cryosectioned slides (in slide box) on dry ice to:

Valerie Calvert
Research Assistant Professor
George Mason University
Center for Applied Proteomics and Molecular Medicine
10920 George Mason Circle Room 2006
Institute of Advanced BioMedical Research
Manassas, VA 20110
Phone: 703-993-3272
Fax: 703-993-9575
email: vcalvert@gmu.edu

Appendix IX: Merck Sharp & Dohme

The collection of tumor samples, along with the baseline and post-dose blood samples, is for the purposes of RNA gene expression profiling. RNA gene expression profiling requires both tumor samples and blood samples for analysis. The following information details the blood sample collection procedures and shipping requirements.

Ashion Analytics will provide Merck with tumor samples (formalin-fixed and paraffin-embedded block created from 1 core obtained at each biopsy time-point and if available archival tissue) as defined in the Laboratory Manual. Serum samples will be shipped to Merck Research Laboratories as described below. Whole blood for RNA (PAXgene blood RNA tube) will be shipped to BioProcessing Solutions Alliance as described below.

Note: Whole blood for RNA (PAXgene blood RNA tube) and serum samples can be stored at the site and should be sent in a batch shipment when the patient comes off study to save on shipping costs.

Whole blood for RNA and serum will be collected at the following time-points for patients on a 28 day cycle: day of initial biopsy (prior to actual biopsy procedure); Cycle 1, Day 15 (prior to treatment); and at the time of progression. If a patient responds to treatment during the study, samples will also be collected at the time of clinical response.

Whole blood for RNA and serum will be collected at the following time-points for patients on a 21 day cycle: day of initial biopsy (prior to actual biopsy procedure); Cycle 2, Day 1 (prior to treatment); and at the time of progression. If a patient responds to treatment during the study, samples will also be collected at the time of clinical response.

I. WHOLE BLOOD FOR CORRELATIVE SAMPLES (RNA)

Sample	Whole Blood for Correlative Study (RNA)
Time points	**All patients:** Day of initial biopsy, at the time of clinical response (if any) and at the time of progression<br>**Additionally:**<br>**Patients on 28 day cycle:** Cycle 1, Day 15 (prior to dosing)<br>**Patients on 21 day cycle:** Cycle 2, Day 1 (prior to dosing)
No. of samples per time point	**One 2.5 ml PAXgene Blood RNA tube**
Process	**Collect** blood in one labeled **2.5 ml PAXgene™ RNA** tube.<br><br>Immediately after collection**,** **invert** the tube 8-10 times. 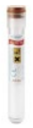

	**Place** tube in -20ºC freezer for a minimum of 24 hours then transfer to -70ºC/-80ºC for longer storage until ready to ship. 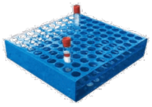
**Storage condition:**	-70ºC/-80ºC
**Shipping condition:**	Dry ice
**Ship to:**	BioProcessing Solutions Alliance<br>**Note:** Whole blood for RNA (PAXgene blood RNA tube) samples can be stored at the site and should be sent in a batch shipment when the patient comes off study to save on shipping costs.

Specimen Collection Notes

- Blood for correlative studies should be drawn prior to dosing at the specified time points. Follow this procedure when collecting, processing and shipping these samples.

Supplies and Materials

1. 2.5 mL PAXgene™ Blood RNA collection tubes
2. One 5 mL "discard tube" tube if the PAXgene™ Blood RNA collection tube is the only tube being drawn
3. Freezer for -20°C for PAXgene™ tube storage, up to 1 month.
4. Freezer for -70/80 °C for PAXgene™ tube storage, greater than 1 month, if necessary
5. A wire rack should be used (NO STYROFOAM) as the tubes may crack.

Precautions

- **SAFETY PRECAUTION:** Contents of the PAXgene™ tube are irritating to skin. Wear disposable gloves, safety glasses or goggles and a laboratory coat and follow standard laboratory safety procedures while working with these tubes. If inhaled, supply fresh air; consult doctor in case of complaints. If skin contact, immediately wash with water and soap, and rinse thoroughly. In case of eye contact, rinse opened eye for 15 minutes under running water, then consult a doctor. If swallowed, immediately call a doctor.

Labeling

Complete label with appropriate information and place one label on the PAXgene™ Blood RNA collection tube. The label must include:

- Protocol Number
- Merck Protocol Number: OTSP 53701
- Site Number
- Patient ID Number

- Visit (e.g. Cycle 1, Day 15)
- Collection Date

Preparation

NOTE: Do not use tube after the expiration date printed on the label.
Ensure the PAXgene™ Blood RNA collection tube is at room temperature prior to collecting blood.

Specimen Collection

NOTE: Collection of specimens from vascular access devices and heparin or saline locks is not recommended due to the potential for specimen contamination. This specimen should be collected as a peripheral blood draw.

1. The PAXgene™ Blood RNA collection tube should not be the first tube drawn during venipuncture. If no other sample collection tube is to be drawn from the same venipuncture, a blank discard tube should be collected first to reduce the possibility of backflow. If additional collection tubes are drawn at the same time point, the PAXgene™ Blood RNA collection tube should be the last tube drawn in the phlebotomy procedure. If compatible, the 12 inch tubing can be used for the additional blood collection tubes (refer to following diagrams).
2. Collect blood into the PAXgene™ Blood RNA collection tube via your institution's recommended standard procedure for venipuncture.
3. The following techniques should be used to prevent possible backflow during the venipuncture:
    a. Place the donor's arm in a downward position.
    b. Hold the PAXgene™ Blood RNA collection tube in a vertical position, below the donor's arm during blood collection.
    c. Release tourniquet as soon as blood starts to flow into tube.
    d. Make sure the tube additives do not touch stopper or end of needle during venipuncture.
4. Collect one tube for total blood collection per time point. Allow at least 10 seconds for a complete blood draw to take place.

    NOTE: After the tube is collected, it is **CRITICAL** to gently invert PAXgene™ 8 to 10 times to ensure proper mixing of blood & PAXgene™ proprietary reagent.

    1. Ensure that the blood has stopped flowing into the tube before removing the tube from the holder.
    2. The tube holds approximately 2.5 ml of blood.
    3. Under-filling of the tube will result in an incorrect blood-to-additive ratio and may lead to poor performance (e.g. poor RNA quality).

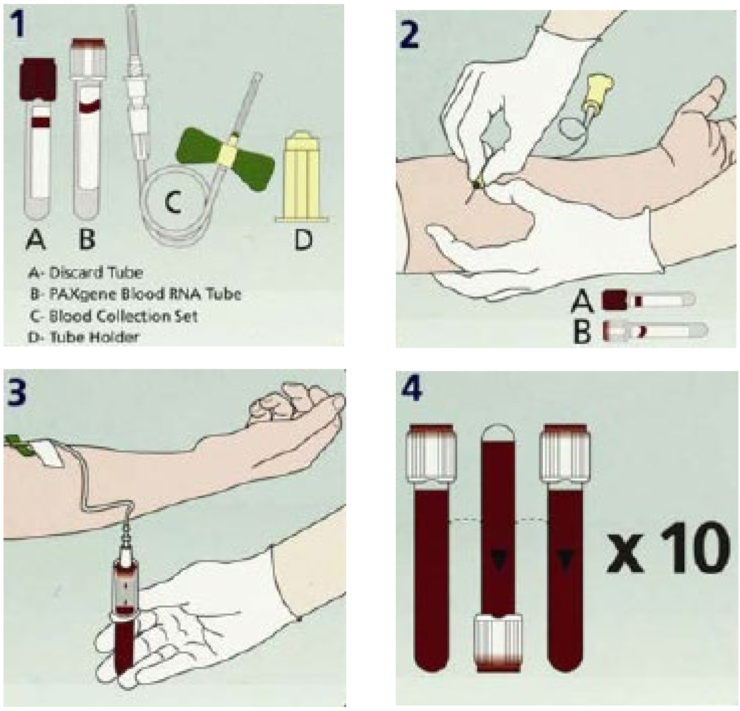

Specimen Processing and Handling

- Immediately after blood collection, gently invert the PAXgene Blood RNA Tube 8–10 times.
- Within 5-10 minutes of the blood draw, place tube upright in a -20°C freezer in a wire rack for a minimum of 24 hours. (do not place in a foam holder as this may cause the tubes to crack).

Storage

NOTE: Frozen PAXgene™ Blood RNA collection tub is subject to breakage on impact. To reduce the risk of breakage during handling and shipment, frozen tube should be treated in the same manner as glass tubes.

- Specimens can remain at -20°C for up to 1 month. For longer term storage, the tubes must be shipped to the Laboratory or transferred to -70/80°C.
- Any temperature excursions should be documented and communicated upon specimen shipment within the shipment inventory documents.

Whole blood for RNA (PAXgene blood RNA tube) samples can be stored at the site and should be sent in a batch shipment when the patient comes off study to save on shipping costs.

Packaging and Shipping

NOTE: Ship Monday through Wednesday only. Contact BioProcessing Solutions Alliance prior to shipping around holidays to confirm their availability.

- Follow packing and shipping instructions for DRY ICE shipments

1. It is the responsibility of the primary investigator/shipper to ensure that all staff personnel who will be handling, packaging, and/or shipping clinical specimens are trained and certified as required by National and International regulations and they ship materials in accordance with all current regulations relating to the handling and shipping of hazardous goods.
2. All samples must be shipped frozen on dry ice (20kg of dry ice).
3. Prepare samples for shipment only on the day of transit.
4. Always use a Merck approved courier (especially international). FedEx will be used for shipping domestically. International shipments should use World Courier.
5. **Shipping schedule** – Select overnight or priority delivery and ensure that shipments are received at the destination Vendor Monday through Thursday, except on U.S. holidays. **Contact the Vendor if you are uncertain about the shipping or receiving schedule.**

   NOTE: International shipments may require additional shipping paperwork. Consult the appropriate shipping company.

- Complete and submit shipping manifest in Excel format to BioProcessing Solutions Alliance **ONLY** on the day of shipment.
  Email to Bioprocessing.Solutions@Biostorage.com
- Ship frozen samples with a copy of the shipping manifest to:

  BioProcessing Solutions Alliance
  Attn: CommStaff
  Nelson Biological Laboratories
  604 Allison Road, C120
  Piscataway, NJ 08854
  Phone: +1 (848) 445 – 1498
  Fax: +1 (848) 445 – 1149
  Email: Bioprocessing.Solutions@biostorage.com

General Precautions: Sample Preparation, Collection, and Processing

- Wear gloves at all times
- Ensure all labeling is completed prior to collecting the sample
- Labels must not overlap
- Do not write over or modify the patient identifiers on the labels
- Samples must be processed immediately after they are collected from the patient

II. SERUM SAMPLE COLLECTION PROCEDURE

Specimen Collection Notes

- Serum will be collected at the following time-points for patients on a 28 day cycle: day of initial biopsy (prior to actual biopsy procedure); Cycle 1, Day 15 (prior to treatment); and at the time of progression. If a patient responds to treatment during the study, samples will also be collected at the time of clinical response.
- Serum will be collected at the following time-points for patients on a 21 day cycle: day of initial biopsy (prior to actual biopsy procedure); Cycle 2, Day 1 (prior to treatment); and at the time of progression. If a patient responds to treatment during the study, samples will also be collected at the time of clinical response.
- **NOTE:** Collection of specimens from a vascular access device or heparin / saline locks is not recommended due to the potential for specimen contamination. This specimen should be collected as a peripheral blood draw.

Supplies and Materials

- One (1) 3mL Serum red top Vacutainer collection tube (BD #366668)
- One(1) appropriate phlebotomy needle
- Two (2) 2.0mL Sarstedt tubes (Sarstedt #72.694.106)
- One (1) 3.6 mL NUNC collection tube (Thermo Scientific #366524)
- Transfer Pipettes
- Boxes for storage of the 2.0 mL Sarstedt tubes
- Serum Biomarker labels
- Inventory Form

Required Equipment

- Centrifuge for Vacutainer tubes capable of rotating at 1500 x g
- -70/-80°C for sample storage (preferred), or -20°C (alternate)

Labeling

1. Complete label with appropriate information and place one on each of the two (2) 2.0mL Sarstedt and the 3.6 mL NUNC collection tube The label must include:
    - Protocol Number
    - Merck Protocol Number: OTSP 53701
    - Site Number
    - Patient ID Number
    - Visit (e.g. Cycle 1, Day 15)
    - Collection Date

    a. **SERUM BIOMARKER 1 – 2 (2.0mL Sarstedt tubes)**

    b. **Collection Tube (3.6mL NUNC tubes)**
2. Fill out Inventory Form appropriately

Specimen Collection

1. Collect **3mL** of whole blood into the serum red top Vacutainer tube. Fill the collection tube completely or until blood flow stops.

2. After the whole blood collection, gently and completely invert the serum red top Vacutainer tube **5** times to mix uniformly. Allow the sample to stand at room temperature for **30-60 minutes** until the whole blood has been allowed to clot.

Specimen Processing & Handling

***NOTE: Begin processing immediately after clot formation. Freeze samples immediately after processing.**

1. Centrifuge the Vacutainer tube at 1500 x g for 15 minutes.

   ***NOTE:** The RCF varies according to the centrifuge rotor radius. The formula for computing RCF from rotation speed and centrifuge radius is RCF = 11.2r $(RPM/1000)^2$, where r is rotor radius, in cm, and RPM is the rotations per minute setting of the centrifuge.

2. Without disturbing the bottom red blood cell pellet, slowly and carefully collect the serum from the top layer of the tube (***~1.5mL***) using a transfer pipette and transfer into the pre-labelled 3.6mL NUNC tube . Discard the remaining red blood cell pellet appropriately.

   ***NOTE: Do not try to remove all the possible serum. Stay about 5 mm away from the red blood pellet to avoid contamination of serum with the red blood cell pellet at the bottom of the tube.**

3. Mix the serum by inversion 5-6 times, then prepare the pre-defined (see below) aliquots in pre-labeled 2.0mL Sarstedt tubes.

   Aliquot priority by the following list:

   1. **SERUM BIOMARKER 1 1mL in 2.0mL Sarstedt tube**
   2. **SERUM BIOMARKER 2 1mL (or remaining volume) in 2.0mL Sarstedt tube**

4. Transfer the samples in upright position to -70/-80°C freezer (preferred) or -20°C freezer (alternate) for storage.

Specimen Storage

1. Store the samples in at -70/-80ºC (preferred) or -20°C (alternate) until shipment to Merck on DRY ICE.

2. Any temperature excursions should be documented and communicated upon specimen shipment within the shipment inventory documents.

Serum samples can be stored at the site and should be sent in a batch shipment when the patient comes off study to save on shipping costs.

Packaging and Shipping

1. It is the responsibility of the primary investigator to ensure that all staff personnel who will be handling, packaging, and/or shipping clinical specimens are trained and certified as required by National and International regulations and they ship materials in

accordance with all current regulations relating to the handling and shipping of hazardous goods.

2. Contact shipping courier to obtain any required documentation/forms required for shipment.
3. Follow packing and shipping instructions for **DRY ICE** shipments.
4. All shipments should be made in freezer boxes containing at least 20 kg DRY ICE, and labeled as HUMAN SAMPLES: NONINFECTIOUS

***NOTE**: The packaging and shipping instructions provided are not considered nor are they intended to be formal Dangerous Goods training.

5. Include a sample inventory form with each shipment.
6. Ship **Two (2)** 2.0mL Sarstedt tubes to Merck Research Laboratories.
7. Shipments should be sent on a **MONDAY** or **TUESDAY** only to assure receipt by Friday.
8. Ship to the following address:

Merck Research Laboratories
Tissue Accessioning
c/o Kate Bohrer
901 S. California Ave
Palo Alto CA 94304
Phone: 650-496-1158
Email: katherine.bohrer@merck.com

Appendix X: Sample Distribution from Clinical Sites

Requestor	Type	Timepoint(s)	# of Patients	Quantity	Sample Preparation	Shipping
TGen	Blood (plasma and buffy coat) for ctNA studies	1. Pre-treatment biopsy<br>2. Cycle 1, Day 1ǂ<br>3. Cycle 1, Day 8 or 15ǂ<br>4. Day 1 of each subsequent cycleǂ<br>5.. End of study visitǂ	All	10 mL	Plasma – blood will be collected in 1x10 lavender top (EDTA) tube and centrifuged at at 820g for 10 mins at room temperature	Dry Ice (overnight)
Ashion Analytics	Blood (whole blood)	Pre-treatment (at time of pre-treatment biopsy),	All	10-20 mL	lavender top (EDTA) tube	Cool Pack (overnight)
	Tissue (DNA)	1. Pre-treatment biopsy<br>2. End of study visit	All	1 core	Flash frozen	Dry Ice (overnight)
	Tissue (RNA)	1. Pre-treatment biopsy<br>2. End of study visit	All	1 core	Flash frozen	Dry Ice (overnight)
	Tissue (FFPE block)	1. Pre-treatment biopsy<br>2. End of study visit	All	1 core	Formalin-fixed and paraffin-embedded at the clinical site	Cool Pack (overnight)
	*Tissue (Bank)	1. Pre-treatment biopsy<br>2. End of study visit	~66 evaluable patients*	1 core	Flash frozen	Dry Ice (overnight)
	Extra tissue (banked)	Pre-treatment biopsy, end of study visit , or both as available	as available	as available	Flash frozen	Dry Ice (overnight)
	Tissue (archival)	primary or metastatic cancer lesion (initial metastasis preferred, archival tissue collected prior to immunotherapy whenever possible)	All	1 block or 10 unstained slides	Not applicable. Ship within 3 months after enrollment.	Cool pack (block) or room temp (slides)
Mayo Clinic	*Tissue (tumorgraft)	Pre-treatment biopsy	~30 evaluable patients*	1 core	15 mL conical tube with 8 mL transport media.	Frozen and Refrigerated Cool Pack (overnight)
George Mason	Tissue (Proteomics)	1. Pre-treatment biopsy<br>2. End of study visit	All	1 core	OCT embedded and stored at -80ºC	Dry Ice (overnight)
Merck	Blood (Whole blood)	1. Pre-treatment biopsy<br>2. Cycle 1, Day 15 or Cycle 2, Day 1‡<br>3. At Clinical Response<br>4. At Progression	All	2.5 mL	Blood will be collected in PAXgene RNA tube, immediately inverted 8-10 times and placed in freezer within 5-10 minutes of blood draw	Frozen on dry ice (20 kg of dry ice) (overnight or priority delivery)
Merck	Blood (serum)	1. Pre-treatment biopsy<br>2. Cycle 1, Day 15 or Cycle 2, Day 1‡<br>3. At Clinical Response<br>4. At Progression	All	3 mL	Blood will be collected in a serum red top Vacutainer tube, and centrifuged at 1500 x g for 15 minutes	Dry Ice

* One of each patient's core biopsies will be used for one of two purposes: generation of a tumorgrat model at the Mayo Clinic or banking for future research at TGen. If the core is slated for tumorgraft generation, it will be sent to the Mayo Clinic for processing; we plan to generate approximately 30 viable tumorgraft models. For the remaining patients, for whom tumorgraft models will not be generated, the tissue will be sent to TGen and banked for future research.

ǂ Patients who cross-over to molecularly guided treatment will also have blood collected at the following timepoints: Cycle 1, Day 1 (prior to molecularly guided treatment); Cycle 1, Day 8 or 15 (depending on which date their scheduled visit will occur, prior to treatment); Day 1 of each subsequent cycle (prior to treatment), and at End of Study visit

‡ Blood will be drawn as the following timepoints: Patients on 28 day cycle: Cycle 1, Day 15 (prior to dosing); Patients on 21 day cycle: Cycle 2, Day 1 (prior to dosing).

Appendix XI: Acknowledgment of the Investigators

PROTOCOL TITLE: Stand Up To Cancer Consortium: Genomics Enabled Medicine for Melanoma (G.E.M.M.): Using Molecularly-Guided Therapy for Patients with BRAF wild-type (BRAFwt) Metastatic Melanoma

Version Date: ___________________________

Acknowledgement of the Investigator:

1.) I have read this protocol and agree that the study is ethical

2.) I agree to conduct the study as outlined and in accordance with all applicable regulations and guidelines

3.) I agree to maintain the confidentiality of all information received or developed in connection with this protocol

____ ________________________

Signature of Investigator: Date:

____

Name of Investigator (Printed or Typed)

Appendix XII: Description of Tumor Boards

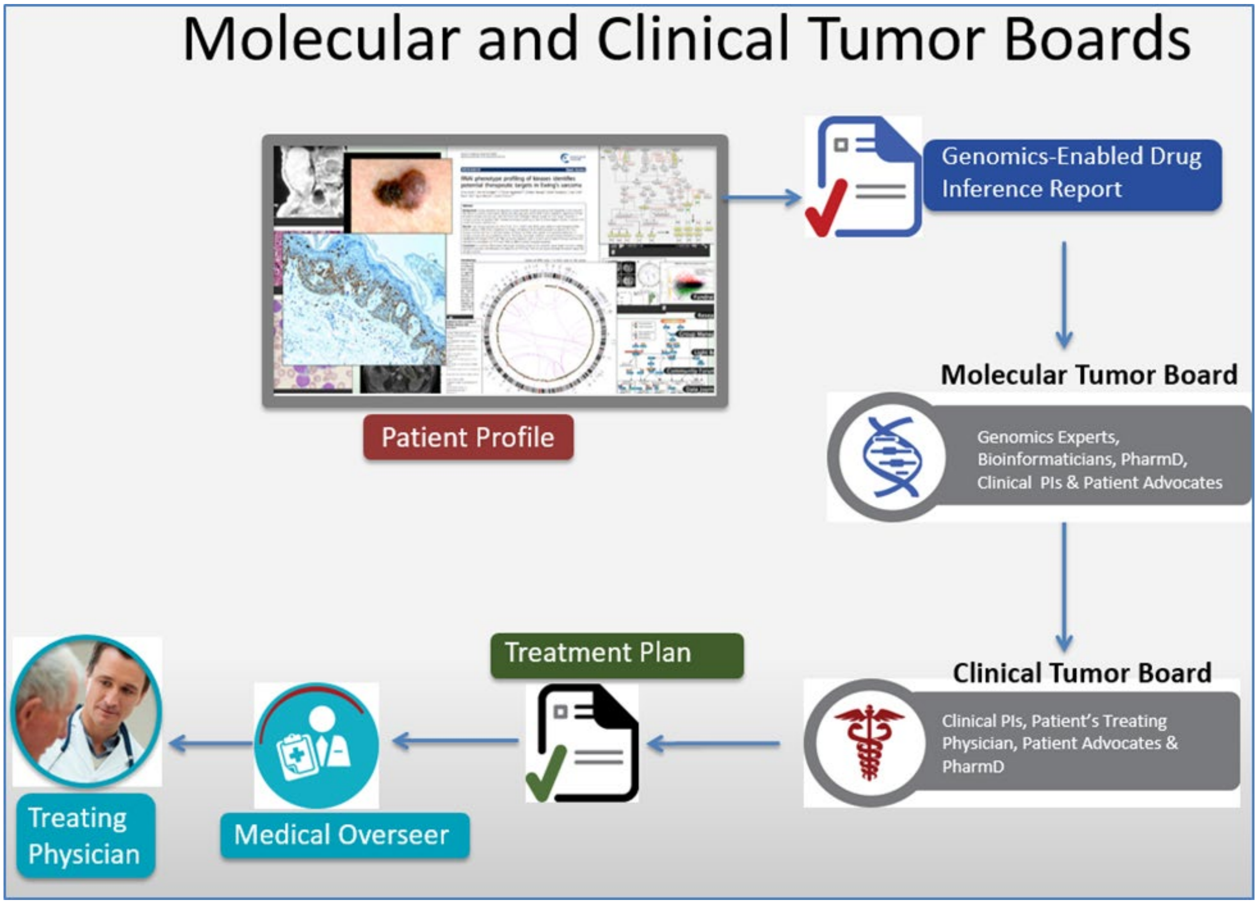

Figure 1. Schematic showing flow of information to and from the Molecular and Clinical Tumor Boards.

Patient Profile

The next generation sequencing approaches employed in the Genomics-Enabled Medicine for Melanoma study will identify somatic events from normal/tumor pairs at the genomic (DNA) level, including coding point mutations and small insertions/deletions, copy number changes, and structural events (intra-chromosomal rearrangements and translocations), and at the transcriptomic (RNA) level including differential gene expression and RNA fusions. These aggregate data sets will be curated and annotated by the Genome Processing and Knowledge Recovery Team at TGen, and a molecular profile will be generated for each patient. This molecular profile will be collected along with the patient's histopathology information and clinical history to form a patient profile. As described in more detail below, this patient profile will be used to generate a Genomics-Enabled Medicine for Melanoma (GEMM) report, which will be shared with the Molecular Tumor Board who will assess the weight of genomic evidence in support of particular target/drug matches.

The Study Pharmacopeia

Investigational agents in the study pharmacopeia were selected under the auspices of the assembled

SU2C team under the direction of Dr. Patricia LoRusso. Selection criteria included: mechanisms of action relevant to non-BRAF mutated melanoma biology; previous knowledge of drug and possible preclinical and clinical efficacy; prior determination of a recommended Phase 2 dose (RP2D) and/or maximally tolerated dose (MTD); and drug availability. Approved oncology agents were selected from broad therapeutic classes based on information available from the Melanoma Disease Model initiative from Cancer Commons and the Melanoma Molecular Map Project. Target/drug matching is performed through curation of PubMed biomedical database and publications to identify those molecular events that alter drug response. The gene/drug annotation framework is based on the Targeted Therapeutic Database maintained by the Melanoma Molecular Map Project. The supporting evidence comes from a variety of sources including PubMed, clinicaltrials.gov, and DrugDex (Thomson Reuters). While the total drug pool available to this study using this approach is currently 326 FDA approved drugs, only those that overlap with the drug list being evaluated in this clinical trial will be considered by the Molecular and Clinical Tumor Boards.

THE Drug KNOWLEDGE Database

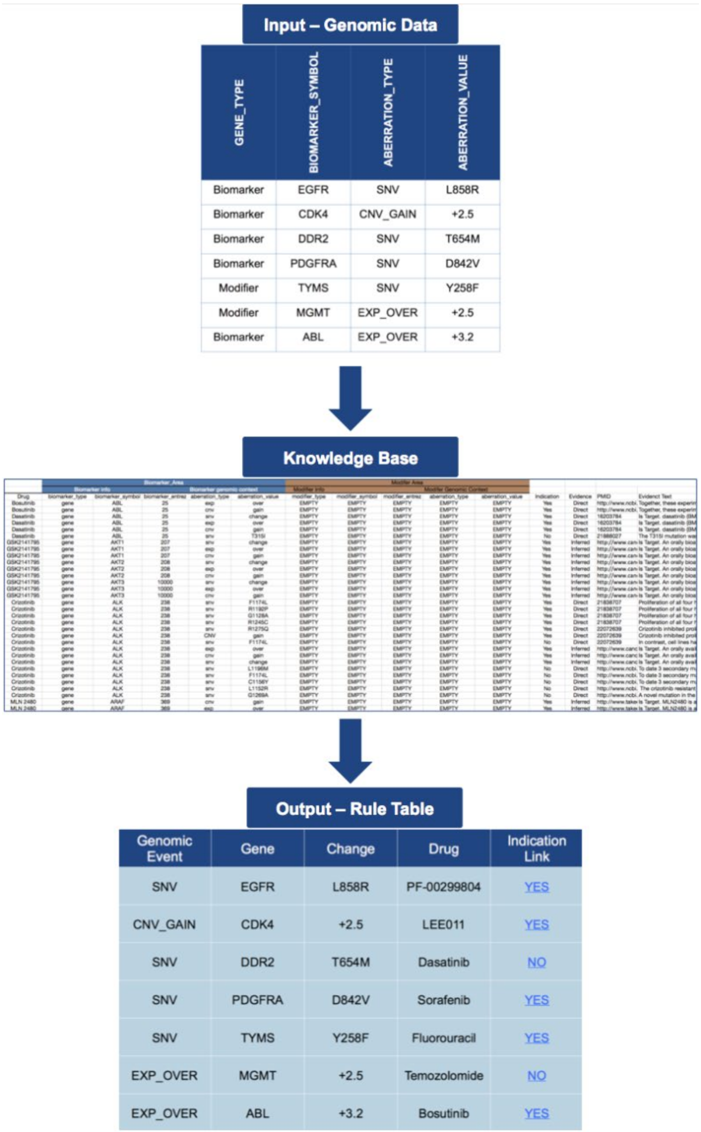

Figure 2. Drug knowledge base and report table. This figure illustrates data types, annotation tables and report outputs. The top panel is an example of genomic data format to be matched against the rules base. The middle panel is an example of a rule annotation table that holds drug to genomic aberration evidence. The bottom panel is an example of rule table to present in report to be presented to tumor board.

Figure 2 illustrates the knowledge library that will be used in the study to match targets to drugs within our pharmacopeia. A target/drug knowledge database was constructed based on a novel conceptual framework and contains defined rules that match specific genomic aberrations to selected therapeutic options contained within the pharmacopeia. The knowledge base is structured with a unique data format and controlled vocabulary. The knowledge base allows for the systematic capture of drug to gene response relationships or "rules". The primary annotation source is the PubMed database, and publications within; however, other sources of information are leveraged when appropriate. The evidence statement behind the annotated relationships is recorded and unique URL referencing the source is also captured.

The conceptual framework, developed to annotate genomic aberrations to drug response, is unique in that it allows for capturing of contextual information other than simple binary gene to drug response relationships. Development of rule structure begins with base statement: 'gene A is target of drug A'. This base relationship is then appended with a **modifier** term that can influence the **state** of the drug target or **biomarker**. The state of the biomarker implies either activation or inactivation of the target gene product. Activation is assumed to suggest efficacy of drug and inactivation would suggest the opposite. There are two forms of modifiers, either **intrinsic or extrinsic**. The **intrinsic modifier** is an inherent property of the biomarker and is defined as the genomic context that influences drug response. The **genomic context** relates to the aberration of the drug target that encompasses DNA amplification/deletion, mutation, mRNA under/overexpression or gene fusion. The **extrinsic modifier** is a defined as a gene, with a distinct genomic context, that influences the state of the target gene and

drug response. The extrinsic modifier may also directly influence drug response in the absence of the drug target.

Operating within the above defined framework relationships between genes, either biomarkers or modifiers, and therapeutic agents, the evidence of the relationship is marked with the **indication** and **evidence** type. The indication of the relationship is recorded as either 'yes' or 'no'. If the relationship between biomarker, extrinsic modifier and drug indicates positive response to the drug, or a sensitive context, it is recorded as a 'yes'. However, if the relationship indicates a negative response to the drug, or a resistant context, it is recorded as 'no'. The evidence type can be of two forms, **direct** or **inferred**. Direct evidence is recorded when the exact relationship between the biomarker, and if applicable the extrinsic modifier, is linked to drug response in the evidence text. Inferred is used when the relationship between the biomarker, and if applicable the extrinsic modifier, is not explicitly linked to drug response in the evidence text. This captures instances where a specific genomic event may not be explicitly linked to drug response but the gene in question is altered. For example, gene 'A' may have a mutation labeled 'B' that is activating; however, only activating mutation 'C' in gene 'A' has published evidence that it is associated with response to drug 'D'. Mutation 'B' in gene 'A' will be annotated, but instead of being labeled as "direct", the label of "inferred" will be applied to that rule statement. Additionally, inferred may be used in cases where the drug may be of the same class and have the same target but is not directly linked to the evidence, but there is evidence that links a drug with the same target and genomic aberrations to a drug response. For example, consider developmental drug 'X' that targets gene 'E'. There is sparse evidence in the literature concerning genomic events that alter response to that drug. However, there exists another drug, 'drug Y', that also targets gene 'E' by a similar mechanism and has multiple observations relating genomic aberrations to response to the drug. This knowledge will be annotated in the knowledge base linked to drug 'X' with evidence type marked as inferred.

> *For example, if PIK3CA is a target, not only might an activating mutation (intrinsic modifier) of PIK3CA represent a sensitivity to a PIK3CA targeted drug, but also, PTEN deletion (extrinsic modifier), by virtue of its normal interference with PIK3CA's activity, may represent an activating event modifying the net result of PIK3CA's activity. Another qualifier for building this rule class will be based on inferred drug class relations to capture relationships based on similarities of drug mechanism. For example, consider drug 'X' that targets PIK3CA kinase activity but does not have specific gene information related to altered response in cancer (as in the case of some experimental agents on the list). In such a case we apply common knowledge of the PIK3CA kinase targeted drug's response performance for use in regards to this agent. The target of the drug, PI3KCA kinase, will serve as the defining drug class definition. These relationships will be explicitly marked as based on common knowledge of this mechanistic class of drugs when conveyed to the Molecular and Clinical Tumor Boards.*

1.) Implementation of Knowledge Library

The drug-rule match algorithm is a mechanistic process operated on the GEMM database that takes as inputs genomic aberrations and their values and identifies the drug candidates from the drug rule match knowledge base described above. The output consists of records containing both the matched biomarkers and drugs with available information in the knowledge base. In particular, this construct

does not restrict the application, and any specific phenotype can be used to match drugs to genomic aberrations in any general setting.

The genomic aberrations are matched to drug rules in the knowledge base using the following criteria:

- Match based on the type of genomic aberration of a biomarker with or without exact match of the biomarker value
- Match based on the type of genomic aberration of a modifier with or without exact match of the modifier value

Genomics-Enabled Medicine for Melanoma (GEMM) Report

Upon execution of the different genomics analytical methodologies and creation of a patient profile, a knowledge mining analysis and translational report (termed a "Genomics-Enabled Medicine for Melanoma Report") will be generated for each patient. Each report will provide an interpretation of the genomic aberration, an evidence-supported mechanistic explanation to describe the contextual vulnerabilities that were found, and a list of recommended FDA-approved drugs and investigational therapies in our study drug pharmacopeia relevant to the aberrations. Aberrations are classified according to the following criteria:

4.) Level 1: Published literature links a particular drug to the molecular aberration
5.) Level 2: Available information (via TCGA, Sanger Cosmic, etc.) indicates a particular aberration in molecular profile is thought to be involved in cancer, but published literature does not link this aberration to a particular drug
6.) Level 3: All other molecular aberrations

Level 1 and Level 2 aberrations will be included in the Genomics-Enabled Medicine for Melanoma Report listed in alphabetical order and presented in an unbiased, non-prioritized fashion. The interactive Genomics-Enabled Medicine for Melanoma report allows the reviewers to quickly navigate to the underlying knowledge and evidence at multiple levels (for example: to attain more information on the specific drug, the drug's putative target(s), clinical trials assessing the predicted drug's efficacy, and other levels of supporting evidence). The report conveys the predicted efficacy of the drugs identified by each of the analytical methods and also highlights evidence that supports or refutes the use of the predicted drug in the context of the patient's disease state (e.g., BRAF wild type metastatic melanoma).

As a result of molecular profiling, there will be three possible scenarios:

4) The patient is found to have a BRAF V600 mutation in contrast to the prior negative results obtained via testing. If this event occurs, the patient will be removed from the study;
5) The profiling results in no potential match with any drug available in our pharmacopeia. In this event, the patient will be removed from the study;
6) The data defines a potential match (or multiple matches) with a targeted therapy that exists in our pharmacopeia. In this scenario, the Genomics-Enabled Medicine for Melanoma report will be completed and distributed to the Molecular Tumor Board.

The Molecular Tumor Board

The Molecular Tumor Board will assess the molecular profile generated for the individual patient and analyze the weight of evidence in support of a proposed target/drug match. The Molecular Tumor Board will meet by conference call/WebEx weekly (or as needed) to review the Genomics-Enabled Medicine for Melanoma reports. Molecular profiling data will be given to the Molecular Tumor Board members a minimum of 48 hours in advance of the meeting. The Molecular Tumor Board will consist of expert members of the team as follows:

- Available clinical PIs
- Genomics experts involved in sequencing and analysis, knowledge mining, and computational and systems biology
- Bioinformaticians
- Pharmacy representation
- Available molecular biologists
- Patient advocates

At least one Genomics expert/bioinformatician, one pharmacy representative, one patient advocate, and three clinical investigators will be needed for a proper quorum to hold a Molecular Tumor Board meeting. Each Molecular Tumor Board conference call will be recorded in its entirety. A summary of the meeting will be written and presented to the Medical Overseer.

The Clinical Tumor Board

A final recommended treatment plan will be generated by the Clinical Tumor Board utilizing the information contained in the knowledge mining analysis, information contained in the translational Genomics-Enabled Medicine for Melanoma report, and relevant patient clinical characteristics (e.g., history & physical, prior treatment history, comorbidities, etc.). Specific treatment details will consist of a regimen chosen from a guided list of agents implicated in critical molecular signaling pathways and/or from signature-based predictions of drug efficacy summarized in the Genomics-Enabled Medicine for Melanoma report. All agents are listed in the current pharmacopoeia we are planning to use in the study, but the selected therapy may differ amongst individual patients depending on several factors, including results of their unique molecular profiling. Potential drug interactions between the targeted agents and the subject's routine medications and supplements will be considered by the Clinical Tumor Board, as well as patient's clinical characteristics and prior treatment history. The decision making process, including prioritization of treatments, will be based on the following:

4.) Depth of knowledge for each individual agent and its response in tumors with similar molecular signatures including (but not limited to)peer-reviewed published *in vivo* data, *in vitro* data, and response profile differential;
5.) Safety considerations, including (but not limited to) review of the patient's history & physical, prior treatments, concurrent medications, and potential drug/drug interactions;
6.) Expertise of team in evaluating each target

The Clinical Tumor Board will meet by conference call/WebEx bi-weekly or more frequently as needed following each Molecular Tumor Board. The Clinical Tumor Board will consist of expert members of the team as follows:

- At least three study clinical investigators
- Additional clinical investigators, as available
- At least one patient advocates (non-voting)
- At least one pharmacy representative (non-voting)

The final treatment recommendation will be made through a majority vote of all clinical investigators in attendance at the Clinical Tumor Board meeting. If a majority vote in favor of a specific treatment is not reached, discussion will be made whether more information is needed, including the possible reconvening of the Molecular Tumor Board, or whether the patient should come off study. In the event of an even number of clinical investigators being present on the call, one will be excluded at random prior to the vote so that a tie will not occur.

Representative members of the Clinical Tumor Board also will participate in the Molecular Tumor Board and thus be familiar with the discussions that have occurred. Each Clinical Tumor Board conference call will be recorded in its entirety. A summary of the meeting will be written and presented to the Medical Overseer. If a particular therapy is recommended, a Treatment Plan will be generated and sent to the Medical Overseer for review.

Treatment Plan

Following discussions by the Clinical Tumor Board, the final Treatment Plan will be prepared for the review of the Medical Overseer. This Treatment plan will include (but will not be limited to) the following:

- Information regarding the drug selected
- Summarized patient information as was presented to the Clinical Tumor Board, including pertinent relevant clinical characteristics including history & physical, comorbidities, prior treatment history, etc.
- Summaries from Molecular and Clinical Tumor Board meetings

Medical Overseer

The final Clinical & Molecular Tumor Board Treatment Plan will be presented to the independent Medical Overseer, who will have the opportunity to reject the purposed regimen requiring reconvening of the Clinical Tumor Board. The independent Medical Overseer will be a board-certified medical oncologist with extensive experience in early clinical trials. He/she will review the Tumor Board treatment recommendations and supervise trial conduct. The Medical Overseer will ensure that the study protocol and procedures were followed in the acquisition and analysis of the patient samples, that the drug suggested by the Genomics-Enabled Medicine Inference Report and recommended by the Clinical Tumor Board is anticipated to be safe for the patient, and that dose and drug administration scheduling are appropriate. The final treatment regimen, decision of the Medical Overseer, and the

summaries of the Molecular and Clinical Tumor Board meetings will be documented. If deemed acceptable, the lead clinical site will be notified.

Treating Physician

The patient will undergo treatment according to the results of the drug assignment, using the appropriate dose and schedule defined by the Clinical Tumor Board and approved by the Medical Overseer. The patient will be given the option to proceed with therapy. He/she, must sign a treatment-specific Consent Memo, which includes a list of known side effects and any additional clinical monitoring that may be required or recommended by the FDA, the pharmaceutical company, and/or the Clinical Tumor Board.

Typical for any patient preparing to undergo cancer treatment, during the five-week window when data is being generated and analyzed, pretreatment evaluation will occur (e.g., CT scans, blood work, etc). If the assigned therapy requires the patient to undergo additional testing specific to a particular drug, it will be performed prior to treatment. We fully recognize several scenarios will exist in which a specific therapy is assigned but the patient will not be able to undergo that particular treatment, including (but not limited to): unique drug-specific inclusion/exclusion restrictions, refusal, physician discretion, co-morbidities, prior treatments, and unexpected events occurring during the five-week analysis period of their tumor. If a patient is unable to receive the assigned treatment due to issues related to eligibility, or refuses to receive the recommended treatment, the patient will come off study.
